# Extensive variation in synonymous substitution rates in mitochondrial genes of seed plants

**DOI:** 10.1186/1471-2148-7-135

**Published:** 2007-08-09

**Authors:** Jeffrey P Mower, Pascal Touzet, Julie S Gummow, Lynda F Delph, Jeffrey D Palmer

**Affiliations:** 1 Department of Biology, Indiana University, Bloomington, IN, 47405, USA; 2 Laboratoire de Genetique et Evolution des Populations Vegetales, UMR CNRS 8016, Universite des Sciences et Technologies de Lille – Lille1, France; 3 Smurfit Institute of Genetics, Trinity College Dublin, Dublin 2, Ireland

## Abstract

**Background:**

It has long been known that rates of synonymous substitutions are unusually low in mitochondrial genes of flowering and other land plants. Although two dramatic exceptions to this pattern have recently been reported, it is unclear how often major increases in substitution rates occur during plant mitochondrial evolution and what the overall magnitude of substitution rate variation is across plants.

**Results:**

A broad survey was undertaken to evaluate synonymous substitution rates in mitochondrial genes of angiosperms and gymnosperms. Although most taxa conform to the generality that plant mitochondrial sequences evolve slowly, additional cases of highly accelerated rates were found. We explore in detail one of these new cases, within the genus *Silene*. A roughly 100-fold increase in synonymous substitution rate is estimated to have taken place within the last 5 million years and involves only one of ten species of *Silene *sampled in this study. Examples of unusually slow sequence evolution were also identified. Comparison of the fastest and slowest lineages shows that synonymous substitution rates vary by four orders of magnitude across seed plants. In other words, some plant mitochondrial lineages accumulate more synonymous change in 10,000 years than do others in 100 million years. Several perplexing cases of gene-to-gene variation in sequence divergence within a plant were uncovered. Some of these probably reflect interesting biological phenomena, such as horizontal gene transfer, mitochondrial-to-nucleus transfer, and intragenomic variation in mitochondrial substitution rates, whereas others are likely the result of various kinds of errors.

**Conclusion:**

The extremes of synonymous substitution rates measured here constitute by far the largest known range of rate variation for any group of organisms. These results highlight the utility of examining absolute substitution rates in a phylogenetic context rather than by traditional pairwise methods. Why substitution rates are generally so low in plant mitochondrial genomes yet occasionally increase dramatically remains mysterious.

## Background

A synonymous site substitution is defined as a change in a protein-coding gene that does not alter the amino acid sequence encoded by the gene. Thus, synonymous substitutions are often assumed to be free of selection at the protein level, and the rates at which they accumulate are widely used as an approximation of the neutral mutation rate. The first study on rates of synonymous substitutions in flowering plants found that mitochondrial genes evolve a few times more slowly than chloroplast genes, about ten times more slowly than plant and mammal nuclear genes, and 50–100 times more slowly than mammalian mitochondrial genes [1]. Later studies have confirmed the low rate of synonymous changes in angiosperm mitochondrial genes [2-4] and extended the observation to the entire mitochondrial genome [5]. However, recent studies identified two genera of flowering plants, *Plantago *and *Pelargonium*, that have experienced a dramatic increase in the mitochondrial rate of synonymous substitution [6-8]. Some of these rate increases were temporary, with rates approaching or returning to normally low levels in certain descendent lineages [6,7].

Phylogenetic analyses have suggested additional cases of rate acceleration for several plant lineages [9-14], but it is unclear whether this is a widespread phenomenon in plants. Here we test the generality of slow synonymous sequence evolution in plant mitochondrial genes across a large number (between 306 and 578 species, depending on the gene) and wide diversity of seed plants (i.e., gymnosperms and angiosperms). Although genes from most species evolve slowly, as expected, additional cases of highly accelerated rates were found, as were examples of exceptionally slow sequence evolution. Surprisingly, a few plants were also identified that contain a mixture of both quickly and slowly evolving mitochondrial genes. Overall, these results demonstrate that the synonymous substitution rate in plant mitochondria is a more variable character than previously appreciated.

## Results

### Levels of sequence divergence in mitochondrial genes of seed plants

**Figure 1:**
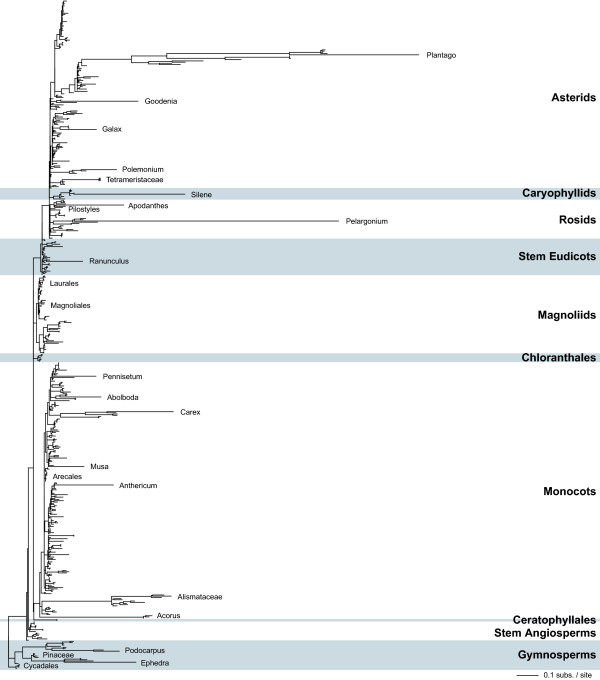
**Synonymous sequence divergence for mitochondrial *atp1***. Shown is the *d*_S _tree resulting from a codon-based likelihood analysis of the mitochondrial *atp1 *gene. Branch lengths are in units of synonymous substitutions per synonymous site. All unique species available in GenBank (546) were included in the analysis. Topological constraints were enforced during the analysis (see methods). Names are shown only for taxa with unusually long or short branches. Figures 1, 2, and 3 are drawn to the same scale.

**Figure 2:**
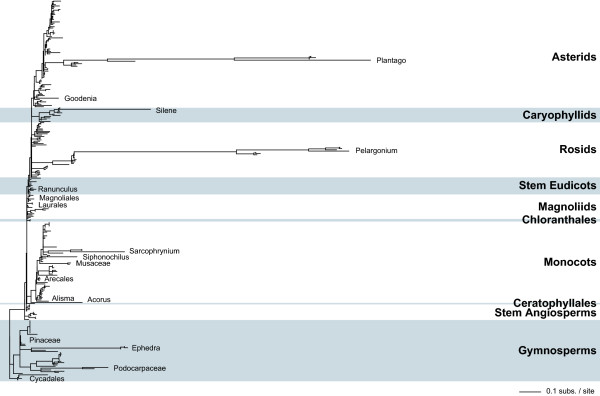
**Synonymous sequence divergence for mitochondrial *cox1***. Shown is the *d*_S _tree resulting from a likelihood analysis of 306 mitochondrial *cox1 *sequences. Details are as in Figure 1.

**Figure 3:**
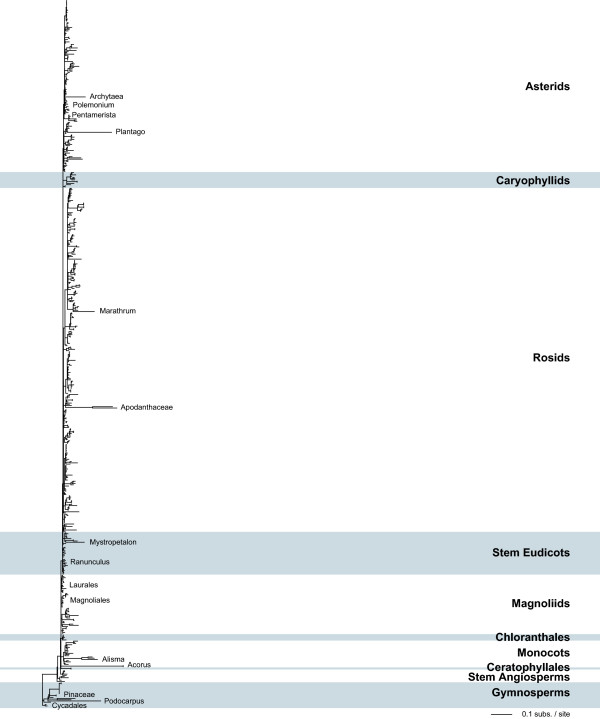
**Synonymous sequence divergence for mitochondrial *matR***. Shown is the *d*_S _tree resulting from a likelihood analysis of 578 mitochondrial *matR *sequences. Details are as in Figure 1. Note that the *matR *gene has not been isolated from *Pelargonium *or the fastest members of *Plantago*.

Previous studies identified two plant lineages, *Plantago *and the Geraniaceae (especially *Pelargonium*), that have independently experienced periods of accelerated substitution rates in their mitochondrial genes [6-8]. To determine whether additional cases of rate acceleration could be found in mitochondrial genes of other seed plants (gymnosperms and angiosperms), blast searches were undertaken in order to collect all available *atp1*, *cox1*, and *matR *homologues from GenBank. These three genes were chosen because they have been used in a number of broad phylogenetic studies and their sequences are available for hundreds of different species of plants. After removing multiple sequences from the same species, pseudogenes, cDNAs, and short gene fragments, a total of 546, 306, and 578 sequences were available for *atp1*, *cox1*, and *matR*, respectively. For each gene, homologous sequences were aligned and maximum likelihood (ML) analyses were performed using the Muse-Gaut codon model [15] to obtain estimates of synonymous site divergence (*d*_S_) per branch (Figs. 1, 2, 3).

A surprising feature of these trees is the amount of branch-length variation seen (Figs. 1, 2, 3). Although the great majority of plants show conventionally low levels of synonymous site divergence, a number of species have substantially shorter or longer branches for one or more genes. The most striking examples of increased sequence divergence are from *Plantago *and *Pelargonium*, whose *atp1 *and *cox1 *branch lengths are exceptionally long (note that the *matR *gene has not been isolated from *Pelargonium *or from the fastest members of *Plantago*), as previously reported [6,7]. Although less extreme, one or more species from *Silene*, *Apodanthes*, *Acorus*, *Ephedra*, and *Podocarpus *are also very divergent across multiple genes. Patterns are not as clear for other taxa. For instance, *Goodenia *and *Musa *are highly divergent for *atp1 *but only moderately divergent for *cox1*. For a number of species, such as *Carex *and *Anthericum*, a sequence from one gene is very divergent but data are not available for the other two genes.

In contrast to the examples of increased levels of mitochondrial sequence divergence, there are several groups that consistently show an unusually low level of divergence, even in the context of the general conservation expected for plants (Figs. 1, 2, 3). Within the angiosperms, these groups include the Arecales (also see [16]), the Chloranthales, the sister orders Laurales and Magnoliales, and most orders within the basal eudicots. Several gymnosperm lineages also have short branches, including the Cycadales, the Pinaceae, and *Ginkgo*.

Unexpectedly, these analyses have also identified several lineages with high levels of divergence for some genes but low levels for others (Figs. 1, 2, 3). For example, divergence levels for *Alisma *are high for *atp1 *and *matR *but unexceptional for *cox1*. The *cox1 *and *matR *genes from *Ranunculus *are remarkable in that they are unusually conserved, similar to the very slow rates found in most basal eudicots, while its *atp1 *gene is moderately divergent. For *Polemonium *and *Pentamerista *(in the Tetrameristaceae), *atp1 *is divergent but *matR *is conserved. Conversely, *matR *is divergent but *atp1 *is conserved for *Pilostyles *(in the Apodanthaceae).

**Figure 4:**
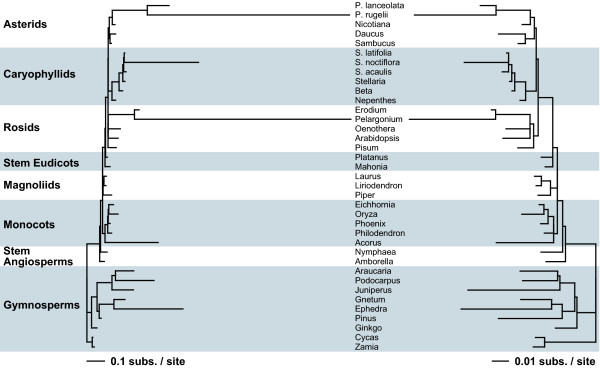
**Multigene analysis of synonymous and nonsynonymous sequence divergence**. Shown are the *d*_S _(left) and *d*_N _(right) trees resulting from a codon-based likelihood analysis of a combined data set of five mitochondrial genes (*atp1*, *cob*, *cox1*, *cox2*, and *cox3*). Branch lengths are in units of synonymous substitutions per synonymous site for the *d*_S _tree and nonsynonymous substitutions per nonsynonymous site for the *d*_N _tree; note the 10-fold difference in scaling between the two trees. Topological constraints were enforced during the analysis (see methods). Sequences for two to five of the genes analyzed were available for any one plant [see Supp. Table 5 in Additional file 2]. S. = *Silene*; P. = *Plantago*.

**Figure 5:**
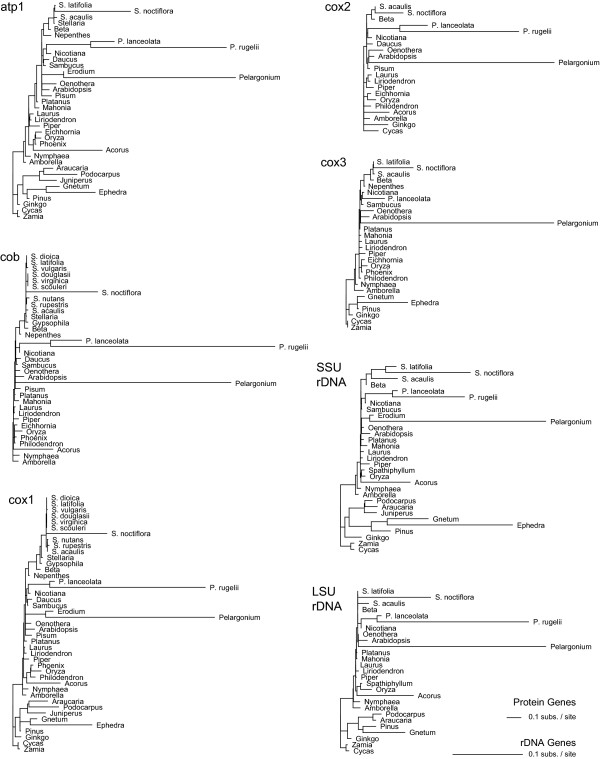
**Individual gene analyses of synonymous sequence divergence**. Shown are the *d*_S _trees resulting from individual, codon-based likelihood analyses of five mitochondrial protein-coding genes (*atp1*, *cob*, *cox1*, *cox2*, and *cox3*) and overall divergence from individual, nucleotide-based likelihood analyses of SSU and LSU rDNA. Branch lengths are in units of synonymous substitutions per synonymous site for the protein-coding genes and in units of substitutions per site for rDNA genes. Topological constraints were enforced during the analysis (see methods). S. = *Silene*; P. = *Plantago*.

To more accurately assess levels of sequence divergence across seed plants, synonymous (*d*_S_) and nonsynonymous (*d*_N_) divergence values were estimated for a subset of taxa from a combined analysis of five mitochondrial protein genes (Fig. 4). Patterns of divergence in the *d*_S _tree from the combined analysis (Fig. 4) are similar to those observed for the trees based on analyses of individual protein-coding genes and rDNA (Fig. 5). In all cases, branch lengths are substantially longer for *Silene noctiflora*, *Acorus*, *Ephedra*, and *Podocarpus *than for most seed plants, but not as long as for *Plantago rugelii *or *Pelargonium hortorum *(Figs. 4, 5). *d*_N _values are also high for these six species, but they are less pronounced than the increases observed for *d*_S _(Fig. 4; note the 10-fold difference in *d*_S _and *d*_N_scales).

### Absolute substitution rates in mitochondrial genes of seed plants

The length of a branch in a phylogenetic tree, which represents an estimate of the amount of sequence divergence for that lineage, equals the product of the absolute substitution rate(s) and time. Because of this confluence, the rate and time components must be separated in order to make direct comparisons of substitution rates among taxa. We previously described a method to calculate absolute rates of synonymous (*R*_S_) and nonsynonymous (*R*_N_) substitution along each branch in a phylogenetic context [6,7], and we employ essentially the same strategy here. However, in this case *d*_S _and *d*_N _values were estimated from a combined analysis of five mitochondrial protein genes (Fig. 4).

There is a wide range of variation in *R*_S _across seed plants (Table 1). At one extreme, the synonymous rate in *Cycas *is only 0.015 substitutions per site per billion years (SSB). Slow rates are also seen for several other gymnosperms, including *Pinus*, *Ginkgo*, and *Zamia*. The *Cycas *value is more than 5-fold lower than the lowest angiosperm rates, from *Platanus*, *Laurus*, *Liriodendron*, *Phoenix*, and *Sambucus*. At the other extreme, *R*_S _is equal to 90 SSB for *Silene noctiflora*. The high rate of synonymous substitution for *S. noctiflora *was recently discovered by a second group as well (DB Sloan, CM Barr, MS Olson, SR Keller, and DR Taylor, personal communication). *R*_S _for *S. noctiflora *is even faster than for those species of *Plantago *and *Pelargonium *sampled here, but lower than the fastest previously reported angiosperm rate of 166–244 SSB for *Plantago media *[6]. Between *Cycas *and *P. media*, synonymous substitutions vary by a factor of 11,000 to 16,000 across seed plants.

**Table 1:** Divergence times, absolute substitution rates, and synonymous to nonsynonymous rate ratios for all terminal branches

**Branch**	**Time (MYA)**	***R*_S _(SSB)**	***R*_N _(SSB)**	***R*_N_/*R***_S_
*P. lanceolata*	17 ± 1	7.71 ± 2.08	0.53 ± 0.13	0.07
*P. rugelii*	17 ± 1	69.62 ± 6.33	2.76 ± 0.30	0.04
*Nicotiana*	82 ± 4	0.19 ± 0.06	0.06 ± 0.02	0.30
*Daucus*	89 ± 5	0.38 ± 0.06	0.17 ± 0.03	0.45
*Sambucus*	89 ± 5	0.10 ± 0.04	0.05 ± 0.01	0.50
*S. latifolia*	5 ± 1	0.54 ± 0.38	0.22 ± 0.12	0.40
*S. noctiflora*	5 ± 1	90.12 ± 13.94	5.37 ± 1.00	0.06
*S. acaulis*	8 ± 1	1.55 ± 0.52	0.45 ± 0.15	0.29
*Stellaria*	19 ± 4	0.59 ± 0.24	0.07 ± 0.06	0.11
*Beta*	38 ± 2	0.56 ± 0.12	0.04 ± 0.02	0.07
*Nepenthes*	76 ± 4	0.29 ± 0.07	0.05 ± 0.02	0.16
*Erodium*	41 ± 3	0.79 ± 0.72	0.06 ± 0.03	0.08
*Pelargonium*	41 ± 3	30.70 ± 3.07	1.22 ± 0.17	0.04
*Arabidopsis*	100 ± 4	0.56 ± 0.07	0.16 ± 0.02	0.28
*Oenothera*	100 ± 4	0.70 ± 0.08	0.14 ± 0.03	0.20
*Pisum*	100 ± 4	0.45 ± 0.07	0.09 ± 0.02	0.20
*Platanus*	130 ± 6	0.08 ± 0.03	0.05 ± 0.01	0.57
*Mahonia*	131 ± 6	0.23 ± 0.05	0.03 ± 0.01	0.14
*Laurus*	133 ± 6	0.11 ± 0.03	0.03 ± 0.01	0.30
*Liriodendron*	133 ± 6	0.09 ± 0.03	0.02 ± 0.01	0.24
*Piper*	137 ± 6	0.36 ± 0.05	0.05 ± 0.01	0.14
*Eichhornia*	86 ± 4	0.33 ± 0.08	0.03 ± 0.01	0.09
*Oryza*	86 ± 4	0.61 ± 0.08	0.15 ± 0.03	0.24
*Phoenix*	92 ± 5	0.11 ± 0.04	0.05 ± 0.02	0.43
*Philodendron*	118 ± 6	0.19 ± 0.05	0.04 ± 0.01	0.23
*Acorus*	127 ± 6	2.45 ± 0.21	0.26 ± 0.03	0.11
*Nymphaea*	153 ± 8	0.26 ± 0.05	0.08 ± 0.02	0.31
*Amborella*	158 ± 8	0.18 ± 0.04	0.07 ± 0.01	0.42
*Araucaria*	245 ± 0	0.43 ± 0.07	0.09 ± 0.01	0.20
*Podocarpus*	245 ± 0	0.93 ± 0.10	0.08 ± 0.02	0.09
*Juniperus*	267 ± 8	0.47 ± 0.06	0.20 ± 0.02	0.43
*Pinus*	286 ± 13	0.04 ± 0.02	0.13 ± 0.02	3.27
*Gnetum*	169 ± 27	0.38 ± 0.10	0.11 ± 0.03	0.28
*Ephedra*	169 ± 27	2.36 ± 0.43	0.31 ± 0.06	0.13
*Ginkgo*	315 ± 5	0.10 ± 0.02	0.04 ± 0.01	0.39
*Cycas*	179 ± 9	0.02 ± 0.01	0.03 ± 0.01	1.94
*Zamia*	179 ± 9	0.06 ± 0.02	0.03 ± 0.01	0.59

**Figure 6:**
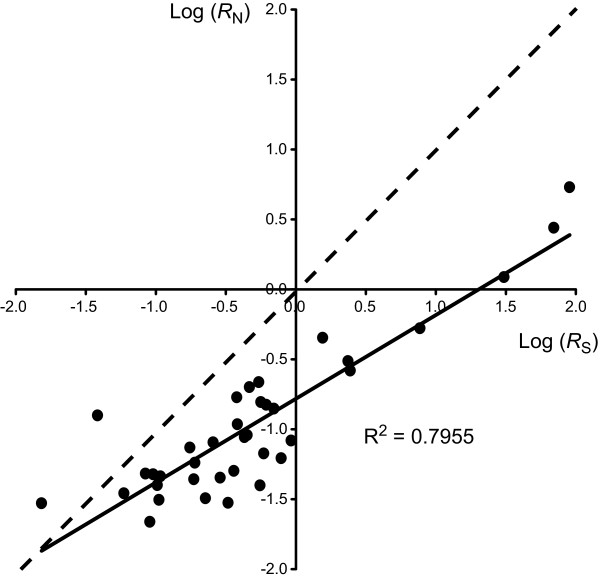
**Correlation between absolute synonymous and nonsynonymous rates**. Absolute rates of nonsynonymous (*R*_N_) and synonymous (*R*_S_) substitution from Table 4 were log-transformed and then plotted for each terminal branch. Log(*R*_S_) values are on the horizontal axis and Log(*R*_N_) values are on the vertical axis. The solid black line is a linear regression of the data. The broken line signifies an *R*_N _to *R*_S _ratio of 1.

*R*_N _is also variable across seed plants, ranging by a factor of 250 between *Liriodendron *and *S. noctiflora *(Table 1). There is a general correlation between *R*_N _and *R*_S _(R^2 ^= 0.80); however, *R*_N _values are muted relative to their *R*_S _counterparts (Figs. 4 and 6). Consequently, *R*_N_/*R*_S _ratios (Table 1) are generally lower for species with high *R*_S _(e.g., *P. rugelii*, *S. noctiflora*, and *Pelargonium*) and higher for species with low *R*_S _(e.g., *Cycas*, *Pinus*, and *Platanus*). These ratios suggest very different evolutionary environments at the extremes. For *P. rugelii *and *Pelargonium*, substitutions at synonymous sites occur 25 times more frequently than at nonsynonymous sites, whereas for *Pinus *and *Cycas*, nonsynonymous substitutions are actually estimated to occur 2–4 times more often than synonymous substitutions.

It must be noted here that sites of RNA editing were not excluded in the analyses of this study. In plants, RNA editing usually occurs at multiple positions for any mitochondrial transcript [17,18]. The retention of edited sites in rate analyses can sometimes bias estimates of nonsynonymous substitution rates, although their presence seems to have little to no effect on synonymous rate estimates [19,20]. Indeed, the exclusion of edited sites in gymnosperm *cox1 *sequences resulted in a 30-fold reduction in the nonsynonymous rate [19]. Thus, the high *R*_N_/*R*_S _ratios observed for *Pinus *and *Cycas *are probably an artifact of the large number of edited sites present in their genes.

**Figure 7:**
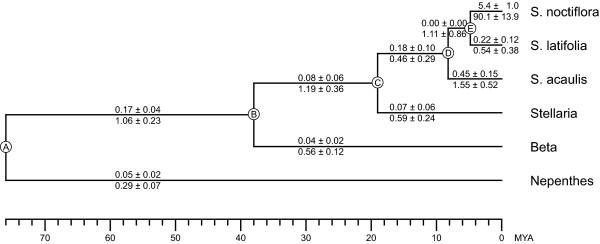
**Absolute substitution rates in Caryophyllales**. Absolute rates of nonsynonymous (*R*_N_) and synonymous (*R*_S_) substitution are plotted above and below, respectively, each branch of the chronogram. *R*_N _and *R*_S _values were calculated as in Table 1, using branch lengths from Figure 4 and divergence times for nodes A and B from Wikstrom et al [45] and for nodes C, D, and E from Supplementary Figure 1 [see Additional file 1]. MYA = million years ago.

To further explore the dynamics of the rate acceleration within *Silene*, *R*_N _and *R*_S _for all internal and terminal branches in the Caryophyllales were plotted phylogenetically (Fig. 7). Only the branch leading to *S. noctiflora *shows a substantial increase in rate, indicating that the rate acceleration occurred within the last five million years, subsequent to the divergence of *S. noctiflora *from the other two species of *Silene *included in this analysis. To determine whether any other species of *Silene *show elevated substitution levels, seven additional species were sampled for the genes *cob *and *cox1*. However, none of the additional taxa appear to be any more closely related to *S. noctiflora *than is *S. latifolia *[see Supp. Fig. 1 in Additional file 1], and none of their sequences are unusually divergent (Fig. 5). Because these additional species did not provide further information on the timing of the rate acceleration, they were not included in the absolute rates analyses in Figure 7.

The frequency of mitochondrial RNA editing was shown to be highly reduced in *Pelargonium *and other Geraniaceae, and this observation was postulated to be potentially related to the increase in their mitochondrial synonymous substitution rate [7]. To discover whether the frequency of editing is also reduced in *S. noctiflora*, edit sites were predicted using the online resource PREP-Mt [21] for the six taxa shown in Figure 7 (Table 2). Contrary to the situation in the Geraniaceae, there appears to be little correlation between substitution rate and editing frequency in *Silene*. The number of predicted edit sites in *S. noctiflora *is quite similar to the other two species of *Silene *and to *Stellaria*, despite the roughly 100-fold increase in the synonymous substitution rate specific to *S. noctiflora *mitochondrial genes.

**Table 2:** Number of RNA editing sites predicted by PREP-Mt (C = 0.5)

**Species**	***atp1***	***cob***	***cox1***	***cox2***	***cox3***
*S. noctiflora*	0	5	1	2	1
*S. acaulis*	2	6	0	2	1
*S. latifolia*	2	7	0	-	1
*Stellaria*	1	7	1	-	-
*Beta*	3	9	0	8	2
*Nepenthes*	4	12	1	-	4

## Discussion

### Absolute substitution rates

Previous studies on synonymous substitution rates found that plant mitochondrial sequences evolve very slowly [1-5]. For the most part, the results presented in Figures 1, 2, 3 confirm these earlier findings. Roughly 80–90% of the sampled species have normally low levels of sequence divergence at synonymous sites. However, a surprising number of species depart from the "normal" mitochondrial pattern. At one end of the spectrum, there are several angiosperm (e.g., Arecales, Chloranthales, and Laurales) and gymnosperm (e.g., Cycadales and Pinaceae) groups whose genes are unusually conserved at synonymous sites. At the opposite end are taxa such as *Plantago*, *Pelargonium*, and *S. noctiflora *with long branches for multiple genes. Many additional species have long branches for a single gene, but independent sequences are needed to confirm that these long branches are replicable and not artifactual (also see next section). On the whole, these results suggest that while most lineages of plant mitochondria do indeed possess generally low synonymous substitution rates, it's increasingly naive and misleading to categorize all plants in this way, and that synonymous substitution rates are, for reasons still unclear, exceptionally fluid and variable in plant mitochondria.

Because the analyses in Figures 1, 2, 3 used almost all species available in GenBank (some species were lost during data filtration; see methods), they provide a generally unbiased representation of rate variation across seed plants. However, for comparisons among species, branch lengths alone are inadequate because they are a function of absolute substitution rate(s) and time. In other words, equally long branches do not necessarily equate to equally fast absolute substitution rates. For example, *d*_S _for *S. noctiflora, Acorus*, and *Ephedra *are quite similar, but *R*_S _for *S. noctiflora *is almost 40 times higher than for *Acorus *and *Ephedra *(Table 1). Indeed, the synonymous rate for *S. noctiflora *is even faster than for the species of *Plantago *and *Pelargonium *examined here (Table 1), despite the much shorter branch length for *S. noctiflora *(Fig. 4).

These findings highlight the importance of taxon sampling in these analyses. The increased sampling of *Silene *species allowed us to pinpoint the rate acceleration in *S. noctiflora *to a 5 million year period. Similarly, sampling of multiple taxa within *Plantago *in a previous study on synonymous rates identified one species, *P. media*, with *R*_S _a few times higher than for *S. noctiflora *over a similar time span [6]. The finding of extensive variation in synonymous substitution rates within these two genera suggests that there is likely to be much more rate variation in plant mitochondrial genomes than is already apparent. Increasingly dense sampling of species is likely to uncover additional examples of rate variation. Calculation of absolute substitution rates may identify further examples, because some increases may be so recent that sequence divergence is still rather low and not readily apparent in phylogenetic trees.

Unfortunately, for *Acorus *and *Ephedra *there is little chance that increased sampling will provide a better estimate of the timing and magnitude of the rate acceleration, despite the fact that their increased divergence levels are spread over more than 100 million years. For *Acorus*, the genus encompasses only three or four species and they all appear to be very similar at the molecular level (see three species in Figure 1 and two species in Figure 3). Furthermore, there are no other genera in the Acorales to break up the time period from the base of monocots to the diversification of *Acorus *[22]. With over 60 species in *Ephedra*, it may be possible to more finely dissect the dynamics of rate evolution in this genus. However, like the situation in *Acorus*, the species included here show very little molecular divergence (see three species in Figure 2) and there are no other genera to break up the long time interval between the crown and stem group nodes of *Ephedra *[22].

Among-species comparisons reveal that synonymous substitution rates vary by four orders of magnitude across seed plants, from a low of 0.015 SSB in *Cycas *(Table 1) to a high of 166–244 SSB in *Plantago media *[6]. Excluding RNA viruses [23], this is, to our knowledge, by far the largest range of synonymous rates known for any phylogenetic group and genome, as well as both the lowest and highest estimated rates. To put this range in perspective, the amount of mitochondrial sequence divergence found in *Cycas *after almost 180 million years of evolution would have taken *P. media *on average only about 15 thousand years to accumulate. These results highlight the advantage of estimating rates in a phylogenetic context. Traditional pairwise methods average rates between two lineages, thereby masking any lineage-specific rate differences. A pairwise comparison between *S. noctiflora *at 90 SSB and *S. latifolia *at 0.54 SSB would result in an averaging of their very different rates to ~45 SSB, which doesn't reflect either of their actual rates very well. Similarly, the low rate found in *Cycas *(0.015 SSB) wouldn't be quite as low in a pairwise comparison with *Zamia *(0.059 SSB) leading to a pairwise rate of 0.037 SSB. The phylogenetic estimates of substitution rates indicate a 6000-fold range of variation between *Cycas *and *S. noctiflora *versus a 1000-fold range based on the pairwise estimates.

Although substitution rates are high for mitochondrial genes of *S. noctiflora *and *Acorus*, chloroplast and nuclear genes from these species do not show any increase in sequence divergence [see Supp. Fig. 1 in Additional file 1] [24-27]. Thus, the rate acceleration in these species is confined to the mitochondrial genome, as seen also in *Plantago *and *Pelargonium *[6,7]. In those studies, defects in mitochondrial DNA replication or repair were speculated to be, among a wide range of processes potentially affecting the mitochondrial mutation rate, the most likely causes of such severe, mitochondrial-specific increases in substitution rates. Similar factors may also be at work in *S. noctiflora *and *Acorus*. In contrast, in *Ephedra*, chloroplast and nuclear sequences are also divergent [27,28]. This points to a factor acting at the organismal level, such as generation time, that would affect rates in all three genomes, although mitochondrial-specific forces may be at work as well. Some studies [16,29-31] have detected moderate, correlated differences in synonymous substitution rates across all three plant genomes and have attributed these to generation time effects [16,29,30], paternal transmission of organelles [31], or correlated substitution and speciation rates [32,33].

### Within-plant variation in sequence divergence

One surprising finding from the broad-scale analyses is the number of plant lineages with levels of synonymous site divergence that appear to be relatively high for some genes and low for others (Figs. 1, 2, 3). In most cases, the basis for this apparent within-plant rate heterogeneity is unknown, and as emphasized below, some of these cases are likely to be or potentially are the result of error of various kinds. Others, however, have a possible biological underpinning. One likely biological explanation is horizontal gene transfer, which occurs surprisingly frequently between mitochondrial genomes of unrelated plants [34,35]. Transfers between genomes with highly different substitution rates and accumulated sequence divergence could easily account for some of the within-plant, gene-to-gene differences shown in Figs. 1, 2, 3 and indeed is suspected to account for one particular case. Two members of the Apodanthaceae, *Pilostyles *and *Apodanthes*, have high levels of divergence for *matR*. The *Apodanthes atp1 *gene is similarly divergent, but *atp1 *from *Pilostyles *is not. Phylogenetic analysis of the *atp1 *gene from *Pilostyles *suggests the possibility that this gene may have been acquired via horizontal transfer from a plant with normally low mitochondrial rates [12].

Another potential biological explanation for differences in synonymous rates between genes from the same plant is that they are located in different genetic compartments that possess different substitution rates. With the exception of *Plantago *and *Pelargonium *[6,7] and probably some of the new cases of high-rate mitochondrial genomes reported in this study, synonymous rates are much lower in mitochondrial than nuclear genomes in plants [1-4]. Functional transfer of a mitochondrial gene to the nucleus will thus usually lead to much higher rates of sequence divergence. This possibility is exciting because there has been no report of functional nuclear transfer for *atp1*, *matR*, or *cox1 *in any plant. This includes no evidence for loss of these three genes from the mitochondrial genomes of 280 diverse angiosperms examined by Southern blot hybridization, whereas 16 other genes were inferred to be frequently lost from the mitochondrial genome and, equally frequently, functionally transferred to the nucleus [36].

A third intriguing possibility is that synonymous substitution rates vary across regions of the mitochondrial genome. In the chloroplast genome of angiosperms, synonymous rates are known to be a few-fold higher in single-copy regions than in a large inverted-repeat region [1,37], and of perhaps greater relevance, a small region with higher accelerated synonymous rates has recently been discovered in the chloroplast of one lineage of legumes (KH Wolfe, personal communication). Gene-to-gene variation in the mitochondrial mutation rate was also recently observed in populations of *Silene vulgaris *[38,39].

There are also several non-biological reasons why divergence levels for a particular species vary between genes. Sequencing errors will artificially inflate sequence divergence. The *Pennisetum atp1 *sequence is a likely example. Including the *atp1 *sequence, eight mitochondrial genes were generated for *Pennisetum *from the same unpublished study (GenBank: AF511559–AF511569), and all show similar characteristics including frameshifting indels and a large number of nucleotide substitutions (data not shown). Most likely, these unpublished sequences are full of errors, and this is the cause of the apparently anomalous divergence of *atp1 *in *Pennisetum*.

Misidentification of the correct phylogenetic position of a sequence/organism could also lead to artifactually long branches (recall that topological constraints were enforced for *d*_S _branch length estimation). For example, two cases were found where the taxonomic sources of sequences were swapped. In the first case, a *cox1 *sequence whose source is listed as *Austrobaileya *(GenBank: AF193954) is more similar to an independent *Ceratophyllum *sequence than to other Austrobaileyales sequences, while a sequence annotated as *Ceratophyllum *(GenBank: AF193945) is actually more *Austrobaileya*-like. These two GenBank files have now been corrected. A similar mix-up occurred between a *Ginkgo*-like sequence annotated as *Cabomba *(GenBank: X94585) and a *Cabomba*-like sequence identified as *Ginkgo *(GenBank: X94587). In each example, the mislabeled sequences were generated from the same study and probably reflect a mix-up that occurred during the database submission process. They were easily identifiable (and were excluded from the final analyses) because they appeared as uncharacteristically long branches relative to independent sequences of the same gene from the same genus.

The use of topological constraints in the analyses may similarly lead to spuriously long branches. To evaluate this possibility, unconstrained analyses were performed on the four data sets used for Figures 1, 2, 3, 4 [see Supp. Figs. 2-3 in Additional file 1]. As can be seen, almost all long branches in the constrained analyses remain long in the unconstrained analyses. However, the use of topological constraints apparently led to an erroneously long branch for *Archytaea matR *and for *Pennisetum atp1*. *Archytaea *was constrained as an asterid in the Ericales (based on GenBank taxonomy), but it is actually a rosid in the Malpighiales [22]. In the unconstrained analysis for *matR*, *Archytaea *groups properly within the Malpighiales and exhibits a more normal branch length [see Supp. Fig. 2 in Additional file 1]. The long branch for *Pennisetum atp1 *also greatly diminishes in the unconstrained analyses [see Supp. Fig. 2 in Additional file 1]. As mentioned above, the long *Pennisetum *branch may result from a poor-quality sequence read. Alternatively, the sequence may have been acquired via horizontal gene transfer, or the DNA sample was derived from an organism mistakenly identified as *Pennisetum*.

These examples underscore the need for independent sequencing to verify that any and all cases of putative intragenomic variation in synonymous substitution rates are real and not the result of human error. For those cases that are validated, it will be interesting to see whether any turn out to reflect noteworthy events in mitochondrial evolution, such as gene- or region-specific differences in substitution rates within a mitochondrial genome, horizontal gene transfer, or the functional transfer to the nucleus of a gene that has never known to have been so-transferred in plants (*atp1 *or *matR*) or even among all eukaryotes (*cox1*).

## Conclusion

In this study, we have measured synonymous substitution rates in a phylogenetic context and uncovered numerous independent examples of rate increase and decrease. These results demonstrate that the synonymous substitution rate in plant mitochondria is a more variable character than previously appreciated. The extremes of synonymous substitution rates measured here constitute by far the largest known range of rate variation for any group of organisms, yet there is no obvious explanation for these divergent patterns. Future studies are required to understand the evolutionary processes driving these patterns.

## Methods

### Molecular techniques

Total genomic DNA was extracted and purified from fresh leaves using a CTAB protocol [40] or using the DNeasy Plant Mini Kit (QIAGEN) according to manufacturer's instructions. Genes were amplified by polymerase chain reaction using a PTC-200 thermocycler (MJ Research) and gene-specific primers [see Supp. Table 1 in Additional file 2]. Each reaction was performed using 35 cycles of 30 sec at 94°C, 30 sec at 50°C, and 2.0–2.5 min at 72°C, with an initial step of 3 min at 94°C and a final step of 10 min at 72°C. Polymerase chain reaction products were purified using ExoSAP-IT (United States Biochemical) and then sequenced on both strands using an ABI 3730 (Applied Biosystems) at the Indiana Molecular Biology Institute. Sequences newly determined in this study were deposited in GenBank under accessions EF547202–EF547251. Additional nucleotide sequences used in this study were obtained from GenBank [see Supp. Tables 2-7 in Additional file 2].

### Survey of mitochondrial sequence divergence

Similarity searches were performed with blastn against the nonredundant GenBank database using the *atp1*, *cox1*, and *matR *genes from *Arabidopsis thaliana *as queries (GenBank: Y08501). Limitations were enforced during the searches such that only hits from the Spermatophyta (gymnosperms and angiosperms) with an e-value less than 1e^-10 ^were included. Hits less than 200 bp in length, multiple hits to the same species, pseudogenes, and cDNA sequences were removed from the blast results, while sequences generated in this study were added. Sequences were aligned using ClustalX and manually adjusted when necessary. Poorly alignable regions were excluded from the data sets, as were regions with gaps in the majority of taxa. These data sets (and all others used in the paper) are available [see Additional file 3].

For each surveyed gene, a ML topology was determined with PAUP*. To ensure completion of the ML analysis, several time-saving steps were taken. First, a partially-constrained topology was enforced. The topology was constrained such that all sequences within a family (as defined by GenBank taxonomy) were forced to be monophyletic. Relationships among families were constrained according to information from the Angiosperm Phylogeny Website [22]. Relationships among the major seed plant groups were constrained according to the 9-gene ML analysis of Qiu et al [41]. Second, ML parameters were fixed to values estimated from a neighbor-joining prior to the ML analysis. Third, the ML analysis used the HKY+G+I model with four rate categories rather than the more parameter-rich GTR+G+I model. The ML topology was identified by a heuristic search starting from the aforementioned neighbor-joining tree and using the TBR branch-swapping algorithm and the MULTREES option.

To determine whether the PAUP* time-saving strategies had an effect on the results of this study, a ML topology was also determined for each gene using RAxML version 2.2.3 [42]. In contrast to the PAUP* analysis, no topological constraints were enforced, the GTR+G model was used (GTR+G+I is not available in RAxML), the search started from a maximum parsimony tree, and the ML parameters were estimated during the ML analysis. For each gene, 10 replicates were performed starting from 10 different randomized parsimony trees. The initial rearrangement setting was fixed to 10 for all analyses.

Using the ML tree topologies generated above for each gene, *d*_S_branch lengths were estimated in HyPhy version 0.99 for UNIX [43] using the MG94W9 codon model [15] and allowing for independent *d*_N _and *d*_S _values for each branch (the local parameters option). The *d*_S _trees using the PAUP* topologies are shown in Figures 1, 2, 3, and the *d*_S _trees based on RAxML topologies are shown in Supplementary Figure 2 [see Additional file 1].

### Estimation of absolute substitution rates

Absolute substitution rates were calculated for a subset of the taxa in the rate survey using methods that have been described previously [6,7]. Briefly, divergence times within Caryophyllaceae [see Supp. Fig. 1B in Additional file 1] were estimated by penalized likelihood with the program r8s [44], using an ML tree from an analysis of the chloroplast gene *matK *[see Supp. Fig. 1A in Additional file 1] and 38 million years for the split between Caryophyllaceae and Amaranthaceae as a calibration point [45]. Standard errors for Caryophyllaceae divergence times were estimated from 100 bootstrap replicates. Divergence times and associated errors for *Plantago*, for the remaining angiosperms, and for gymnosperms were taken from the analyses of Cho et al [6], Wikstrom et al [45], and Magallon and Sanderson [27], respectively. The *d*_S _and *d*_N _trees in Figures 4 and 5 were estimated from individual and combined data sets of five mitochondrial protein-coding genes (*atp1, cob, cox1, cox2, cox3*) using HyPhy as described in the previous section on a topology constrained according to the Angiosperm Phylogeny Website [22]. Standard errors for *d*_S _and *d*_N _were estimated from 100 bootstrap replicates. *R*_S _and *R*_N _values for each branch were calculated by dividing the mitochondrial sequence divergences from the combined data set by the elapsed time along that branch (Table 1).

## Authors' contributions

JPM, PT, and JSG generated sequence data. JPM and JDP designed the seed plant rate survey and interpreted its results. JPM, PT, LFD, and JDP designed the *Silene *rates study and interpreted its results. JPM ran all analyses and prepared all figures and tables. JPM and JDP wrote the manuscript. All authors read and approved the final manuscript.

## Supplementary Material

Additional file 1 Supplementary figures. Supplementary Figure 1 shows the phylogenetic and divergence time analysis for Caryophyllales. Supplementary Figure 2 shows unconstrained analyses of the data sets used for Figures 1, 2, 3. Supplementary Figure 3 shows an unconstrained analysis of the data set used for Figure 4. Supplementary Figures 4-6 show the *d*_S _trees in Figures 1, 2, 3 at an expanded scale and with all taxon names included. Click here for file

Additional file 2 Supplementary tables. Supplementary Table 1 lists the primers used in this study. Supplementary Tables 2-7 list the taxon names and GenBank accession numbers of all sequence used in this study. Click here for file

Additional file 3 Data sets. Data sets 1–7 are in nexus format and contain sequences used in the analyses of *atp1*, *cox1*, *matR*, 5-Gene combined, SSU rDNA, LSU rDNA, and *matK*, respectively. These seven data sets are provided as a tar archive compressed with gzip. Click here for file
