# Supplementary material for: Extensive variation in synonymous substitution rates in mitochondrial genes of seed plants
Source: BMC Evol Biol. 2007 Aug 9;7:135. doi: 10.1186/1471-2148-7-135 (PMC1973135; doi:10.1186/1471-2148-7-135)
Supplement: Additional file 1 — Supplementary figures. Supplementary Figure 1 shows the phylogenetic and divergence time analysis for Caryophyllales. Supplementary Figure 2 shows unconstrained analyses of the data sets used for Figures 1, 2, 3. Supplementary Figure 3 shows an unconstrained analysis of the data set used for Figure 4. Supplementary Figures 4-6 show the dS trees in Figures 1, 2, 3 at an expanded scale and with all taxon names included. [file 1471-2148-7-135-S1.pdf]

Supplementary Figure 1 - Caryophyllaceae phylogeny and divergence times. (a) Phylogeny of Caryophyllaceae and sister families (Amaranthaceae, Achatocarpaceae) resulting from a maximum likelihood analysis of the chloroplast *matK* gene using a GTR+G+I model in PAUP*. The five taxa at bottom were defined as the outgroup. Numbers above each node represent bootstrap values from 100 replicates. Only values above 50% are shown. (b) Chronogram of Caryophyllaceae resulting from a penalized likelihood analysis of the *matK* ML tree shown in (a) using the program r8s. To calibrate the tree, a divergence time of 38 Myr was used for the split between Caryophyllaceae and the sister families Amaranthaceae and Achatocarpaceae (Wikstrom et al., 2001). S. = *Silene*.

Supplementary Figure 2 - Unconstrained survey of synonymous sequence divergence. Shown are the d_S trees from unconstrained analyses of the *atp1* data set used in Figure 1, the *cox1* data set used in Figure 2, and the *matR* data set used in Figure 3. Taxa with long branches that were labeled in Figures 1–3 are also labeled here for comparison.

Supplementary Figure 3 - Unconstrained multigene analysis of synonymous and nonsynonymous sequence divergence. Shown are the d_S (left) and d_N (right) trees from an unconstrained analysis of the combined 5-gene data set used in Figure 4. P. = *Plantago*; S. = *Silene*.

Supplementary Figure 4 - d_S tree for mitochondrial *atp1* at expanded scale. Shown is the d_S tree from Figure 1 of the main text, except that all taxa are labeled and all non-*Plantago* and non-*Pelargonium* branch lengths are drawn at an expanded scale. Compressed branches for *Plantago* and *Pelargonium* are shown as thickened lines.

Supplementary Figure 5 - d_S tree for mitochondrial *cox1* at expanded scale. Shown is the d_S tree from Figure 2 of the main text, except that all taxa are labeled and all non-*Plantago* and non-*Pelargonium* branch lengths are drawn at an expanded scale. Compressed branches for *Plantago* and *Pelargonium* are shown as thickened lines.

Supplementary Figure 6 - d_S tree for mitochondrial *matR* at expanded scale. Shown is the d_S tree from Figure 3 of the main text, except that all taxa are labeled and all branch lengths are drawn at an expanded scale.

Supp. Fig. 1a

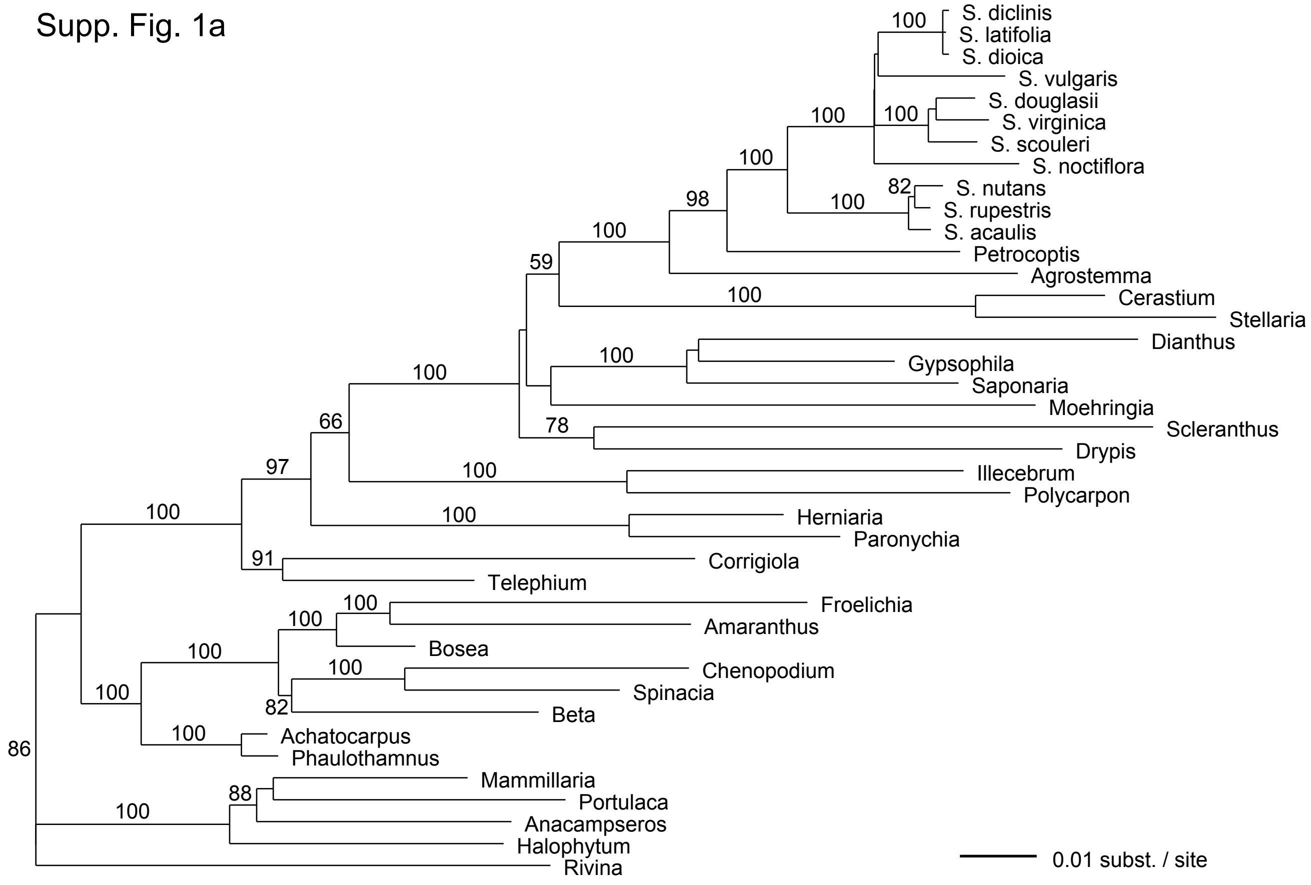

Supp. Fig. 1b

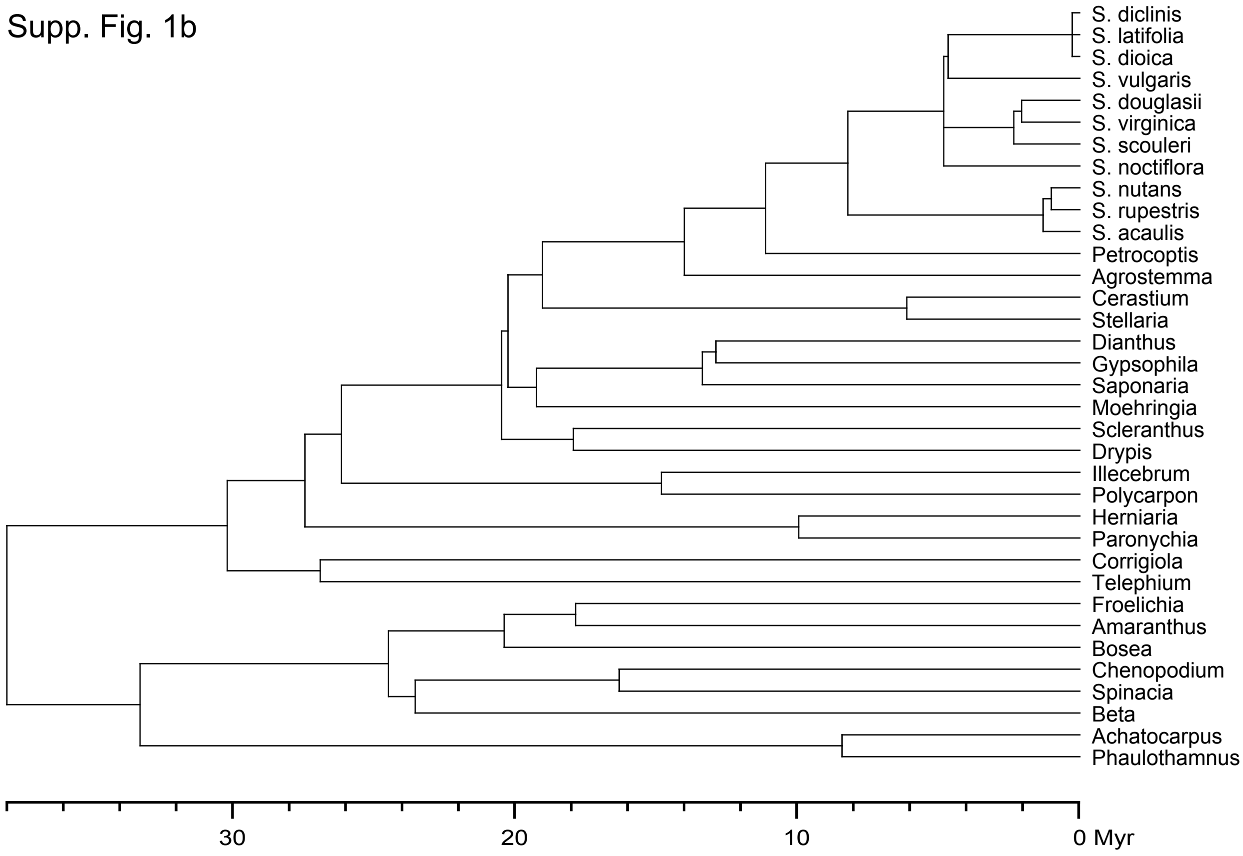

Supp. Fig. 2

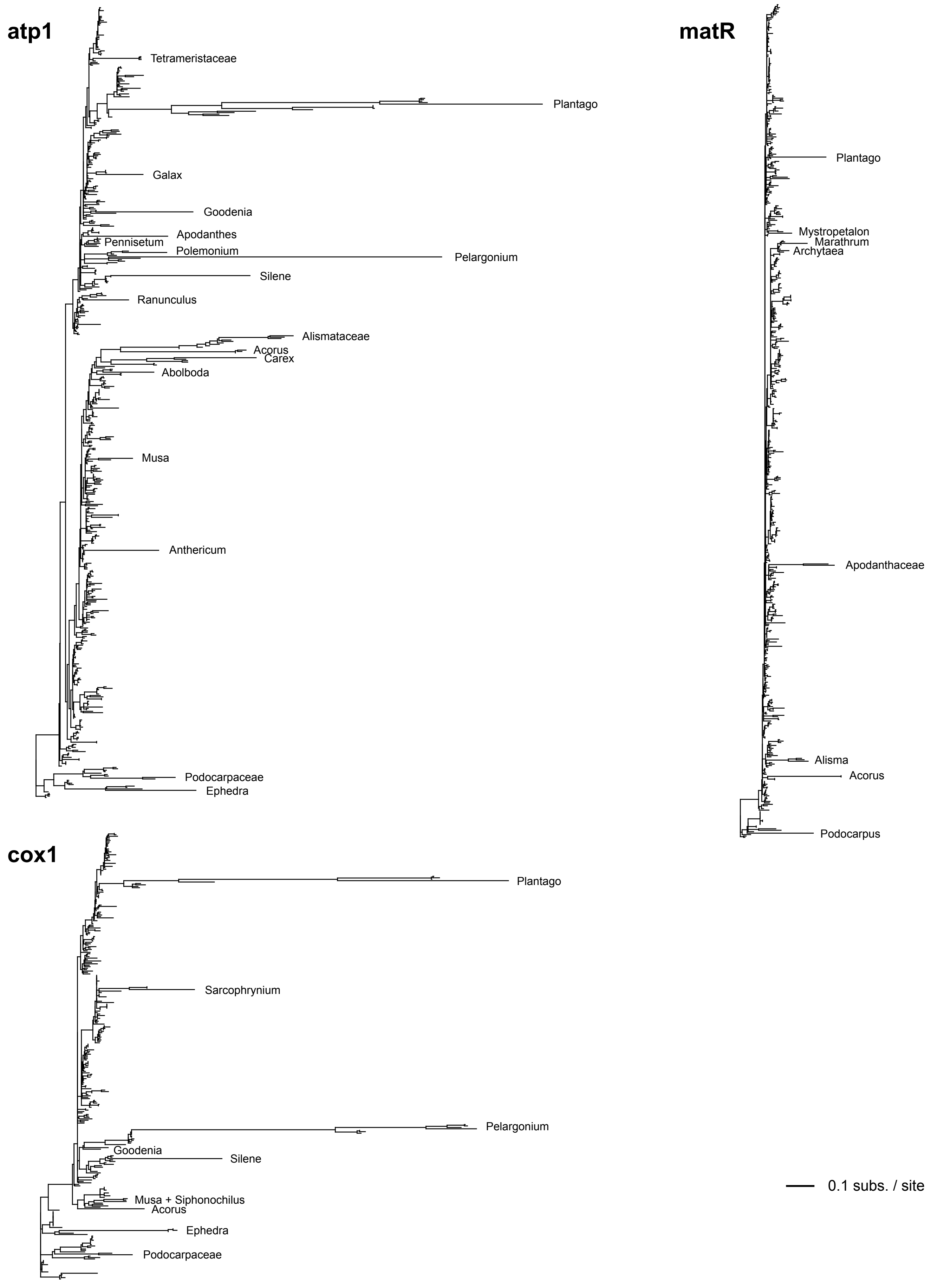
atp1
Tetrameristaceae
Plantago
Galax
Goodenia
Apodanthes
Pennisetum
Polemonium
Pelargonium
Silene
Ranunculus
Alismataceae
Acorus
Carex
Abolboda
Musa
Anthericum
Podocarpaceae
Ephedra
cox1
Plantago
Sarcophrynium
Pelargonium
Goodenia
Silene
Musa + Siphonochilus
Acorus
Ephedra
Podocarpaceae
matR
Plantago
Mystropetalon
Marathrum
Archytaea
Apodanthaceae
Alisma
Acorus
Podocarpus
0.1 subs. / site

Supp. Fig. 3

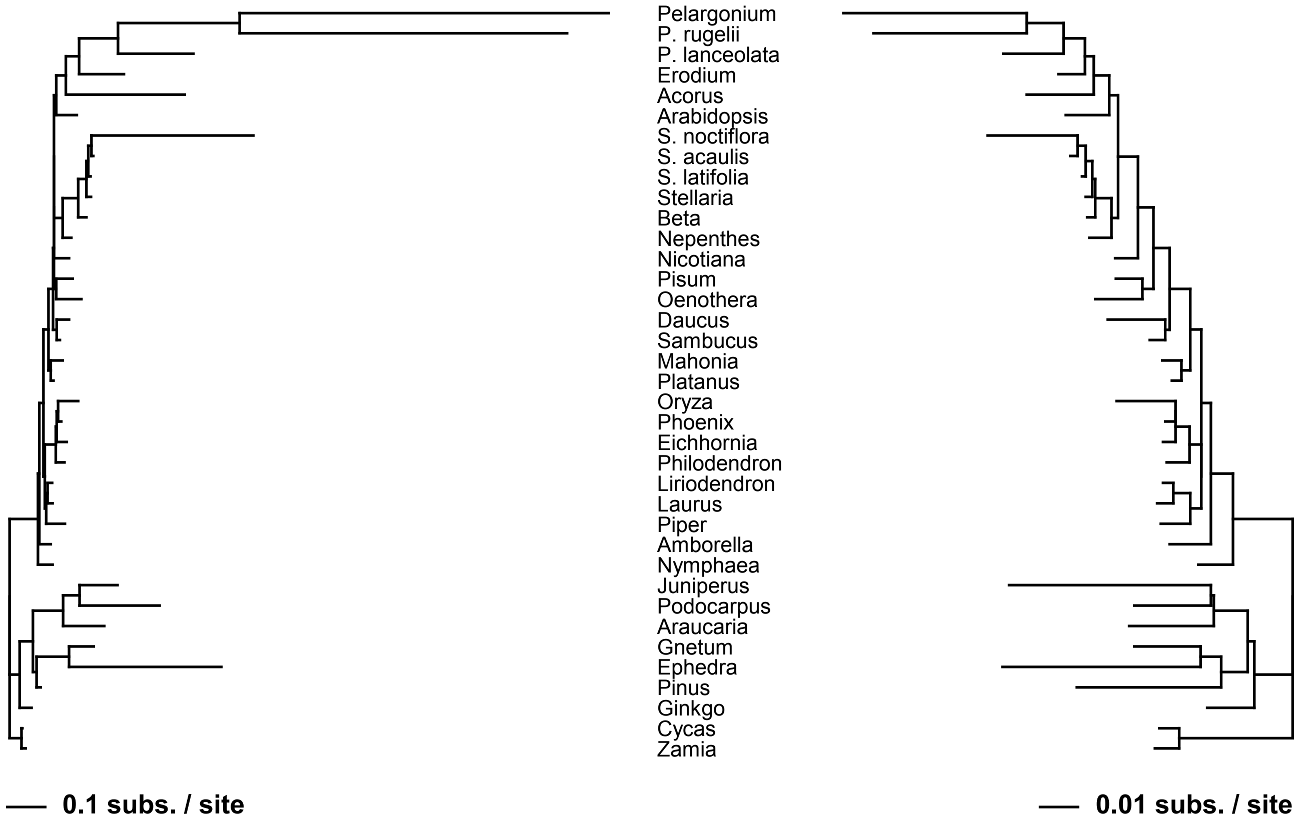

Pelargonium
P. rugelii
P. lanceolata
Erodium
Acorus
Arabidopsis
S. noctiflora
S. acaulis
S. latifolia
Stellaria
Beta
Nepenthes
Nicotiana
Pisum
Oenothera
Daucus
Sambucus
Mahonia
Platanus
Oryza
Phoenix
Eichhornia
Philodendron
Liriodendron
Laurus
Piper
Amborella
Nymphaea
Juniperus
Podocarpus
Araucaria
Gnetum
Ephedra
Pinus
Ginkgo
Cycas
Zamia
0.1 subs. / site
0.01 subs. / site

Supp. Fig. 4a

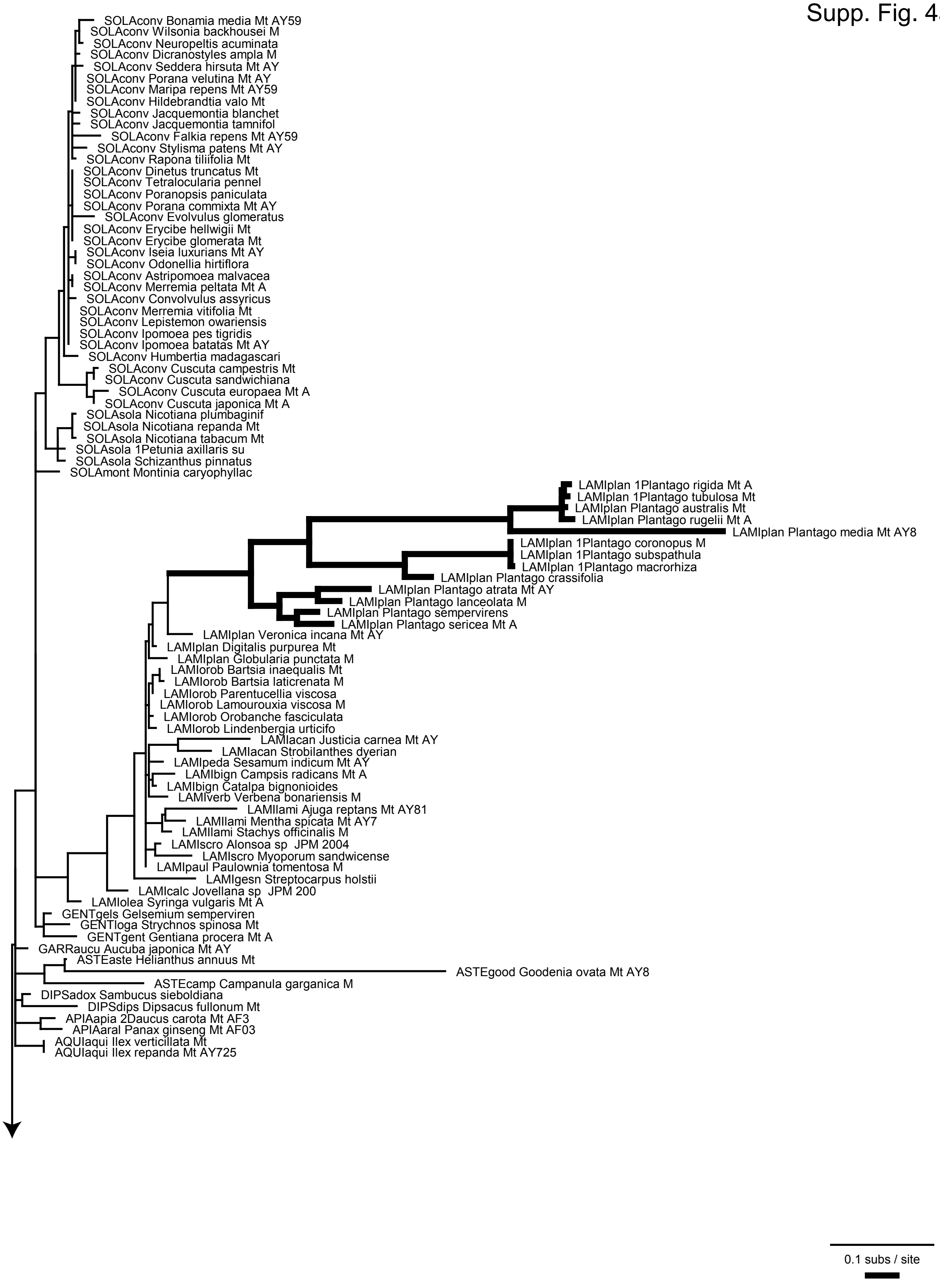

SOLAconv Bonamia media Mt AY59
SOLAconv Wilsonia backhousei M
SOLAconv Neuropeltis acuminata
SOLAconv Dicranostyles ampla M
SOLAconv Seddera hirsuta Mt AY
SOLAconv Porana velutina Mt AY
SOLAconv Maripa repens Mt AY59
SOLAconv Hildebrandtia valo Mt
SOLAconv Jacquemontia blanchet
SOLAconv Jacquemontia tamnifol
SOLAconv Falkia repens Mt AY59
SOLAconv Stylisma patens Mt AY
SOLAconv Rapona tiliifolia Mt
SOLAconv Dinetus truncatus Mt
SOLAconv Tetralocularia pennel
SOLAconv Poranopsis paniculata
SOLAconv Porana commixta Mt AY
SOLAconv Evolvulus glomeratus
SOLAconv Erycibe hellwigii Mt
SOLAconv Erycibe glomerata Mt
SOLAconv Iseia luxurians Mt AY
SOLAconv Odonellia hirtiflora
SOLAconv Astripomoea malvacea
SOLAconv Merremia peltata Mt A
SOLAconv Convolvulus assyricus
SOLAconv Merremia vitifolia Mt
SOLAconv Lepistemon owariensis
SOLAconv Ipomoea pes tigridis
SOLAconv Ipomoea batatas Mt AY
SOLAconv Humbertia madagascari
SOLAconv Cuscuta campestris Mt
SOLAconv Cuscuta sandwichiana
SOLAconv Cuscuta europaea Mt A
SOLAconv Cuscuta japonica Mt A
SOLAsola Nicotiana plumbaginif
SOLAsola Nicotiana repanda Mt
SOLAsola Nicotiana tabacum Mt
SOLAsola 1Petunia axillaris su
SOLAsola Schizanthus pinnatus
SOLAmont Montinia caryophyllac
LAMIplan 1Plantago rigida Mt A
LAMIplan 1Plantago tubulosa Mt
LAMIplan Plantago australis Mt
LAMIplan Plantago rugelii Mt A
LAMIplan Plantago media Mt AY8
LAMIplan 1Plantago coronopus M
LAMIplan 1Plantago subspathula
LAMIplan 1Plantago macrorhiza
LAMIplan Plantago crassifolia
LAMIplan Plantago atrata Mt AY
LAMIplan Plantago lanceolata M
LAMIplan Plantago sempervirens
LAMIplan Plantago sericea Mt A
LAMIplan Veronica incana Mt AY
LAMIplan Digitalis purpurea Mt
LAMIplan Globularia punctata M
LAMIorob Bartsia inaequalis Mt
LAMIorob Bartsia laticrenata M
LAMIorob Parentucellia viscosa
LAMIorob Lamourouxia viscosa M
LAMIorob Orobanche fasciculata
LAMIorob Lindenbergia urticifo
LAMIacan Justicia carnea Mt AY
LAMIacan Strobilanthes dyerian
LAMIpeda Sesamum indicum Mt AY
LAMIbign Campsis radicans Mt A
LAMIbign Catalpa bignonioides
LAMIverb Verbena bonariensis M
LAMIlami Ajuga reptans Mt AY81
LAMIlami Mentha spicata Mt AY7
LAMIlami Stachys officinalis M
LAMIscro Alonsoa sp JPM 2004
LAMIscro Myoporum sandwicense
LAMIpaul Paulownia tomentosa M
LAMIgesn Streptocarpus holstii
LAMIcalc Jovellana sp JPM 200
LAMIolea Syringa vulgaris Mt A
GENTgels Gelsemium semperviren
GENTloga Strychnos spinosa Mt
GENTgent Gentiana procera Mt A
GARRaucu Aucuba japonica Mt AY
ASTEaste Helianthus annuus Mt
ASTEgood Goodenia ovata Mt AY8
ASTEcamp Campanula garganica M
DIPSadox Sambucus sieboldiana
DIPSdips Dipsacus fullonum Mt
APIAapia 2Daucus carota Mt AF3
APIAaral Panax ginseng Mt AF03
AQUIaqui Ilex verticillata Mt
AQUIaqui Ilex repanda Mt AY725
0.1 subs / site

Supp. Fig. 4b

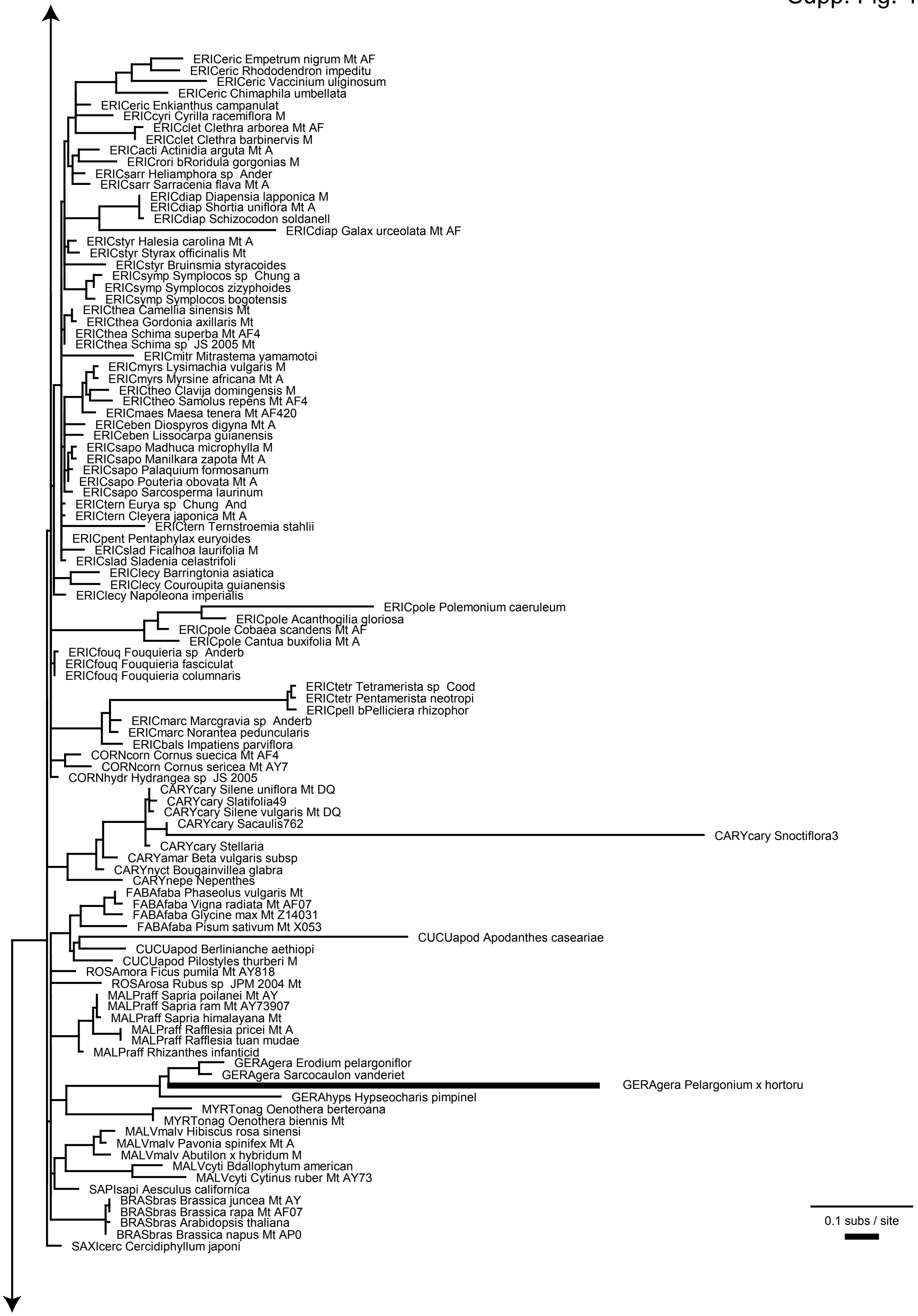
ERICeric Empetrum nigrum Mt AF
ERICeric Rhododendron impeditu
ERICeric Vaccinium uliginosum
ERICeric Chimaphila umbellata
ERICeric Enkianthus campanulat
ERICcyri Cyrilla racemiflora M
ERICclet Clethra arborea Mt AF
ERICclet Clethra barbinervis M
ERICacti Actinidia arguta Mt A
ERICrori bRoridula gorgonias M
ERICsarr Heliamphora sp Ander
ERICsarr Sarracenia flava Mt A
ERICdiap Diapensia lapponica M
ERICdiap Shortia uniflora Mt A
ERICdiap Schizocodon soldanell
ERICdiap Galax urceolata Mt AF
ERICstyr Halesia carolina Mt A
ERICstyr Styrax officinalis Mt
ERICstyr Bruinsmia styracoides
ERICsymp Symplocos sp Chung a
ERICsymp Symplocos zizyphoides
ERICsymp Symplocos bogotensis
ERICthea Camellia sinensis Mt
ERICthea Gordonia axillaris Mt
ERICthea Schima superba Mt AF4
ERICthea Schima sp JS 2005 Mt
ERICmitr Mitrastema yamamotoi
ERICmyrs Lysimachia vulgaris M
ERICmyrs Myrsine africana Mt A
ERICtheo Clavija domingensis M
ERICtheo Samolus repens Mt AF4
ERICmaes Maesa tenera Mt AF420
ERICeben Diospyros digyna Mt A
ERICeben Lissocarpa guianensis
ERICsapo Madhuca microphylla M
ERICsapo Manilkara zapota Mt A
ERICsapo Palaquium formosanum
ERICsapo Pouteria obovata Mt A
ERICsapo Sarcosperma laurinum
ERICtern Eurya sp Chung And
ERICtern Cleyera japonica Mt A
ERICtern Ternstroemia stahlii
ERICpent Pentaphylax euryoides
ERICslad Ficalhoa laurifolia M
ERICslad Sladenia celastrifoli
ERIClecy Barringtonia asiatica
ERIClecy Couroupita guianensis
ERIClecy Napoleona imperialis
ERICpole Polemonium caeruleum
ERICpole Acanthogilia gloriosa
ERICpole Cobaea scandens Mt AF
ERICpole Cantua buxifolia Mt A
ERICfouq Fouquieria sp Anderb
ERICfouq Fouquieria fasciculat
ERICfouq Fouquieria columnaris
ERICtetr Tetramerista sp Cood
ERICtetr Pentamerista neotropi
ERICpell bPelliciera rhizophor
ERICmarc Marcgravia sp Anderb
ERICmarc Norantea peduncularis
ERICbals Impatiens parviflora
CORNcorn Cornus suecica Mt AF4
CORNcorn Cornus sericea Mt AY7
CORNhydr Hydrangea sp JS 2005
CARYcary Silene uniflora Mt DQ
CARYcary Slatifolia49
CARYcary Silene vulgaris Mt DQ
CARYcary Sacaulis762
CARYcary Snoctiflora3
CARYcary Stellaria
CARYamar Beta vulgaris subsp
CARYnyct Bougainvillea glabra
CARYnepe Nepenthes
FABAfaba Phaseolus vulgaris Mt
FABAfaba Vigna radiata Mt AF07
FABAfaba Glycine max Mt Z14031
FABAfaba Pisum sativum Mt X053
CUCUapod Apodanthes caseariae
CUCUapod Berlinianche aethiopi
CUCUapod Pilostyles thurberi M
ROSAmora Ficus pumila Mt AY818
ROSArosa Rubus sp JPM 2004 Mt
MALPraff Sapria poilanei Mt AY
MALPraff Sapria ram Mt AY73907
MALPraff Sapria himalayana Mt
MALPraff Rafflesia pricei Mt A
MALPraff Rafflesia tuan mudae
MALPraff Rhizanthes infanticid
GERAgera Erodium pelargoniflor
GERAgera Sarcocaulon vanderiet
GERAgera Pelargonium x hortoru
GERAhyps Hypseocharis pimpinel
MYRTonag Oenothera berteroana
MYRTonag Oenothera biennis Mt
MALVmalv Hibiscus rosa sinensi
MALVmalv Pavonia spinifex Mt A
MALVmalv Abutilon x hybridum M
MALVcyti Bdallophytum american
MALVcyti Cytinus ruber Mt AY73
SAPIsapi Aesculus californica
BRASbras Brassica juncea Mt AY
BRASbras Brassica rapa Mt AF07
BRASbras Arabidopsis thaliana
BRASbras Brassica napus Mt AP0
SAXIcerc Cercidiphyllum japoni
0.1 subs / site

Supp. Fig. 4c

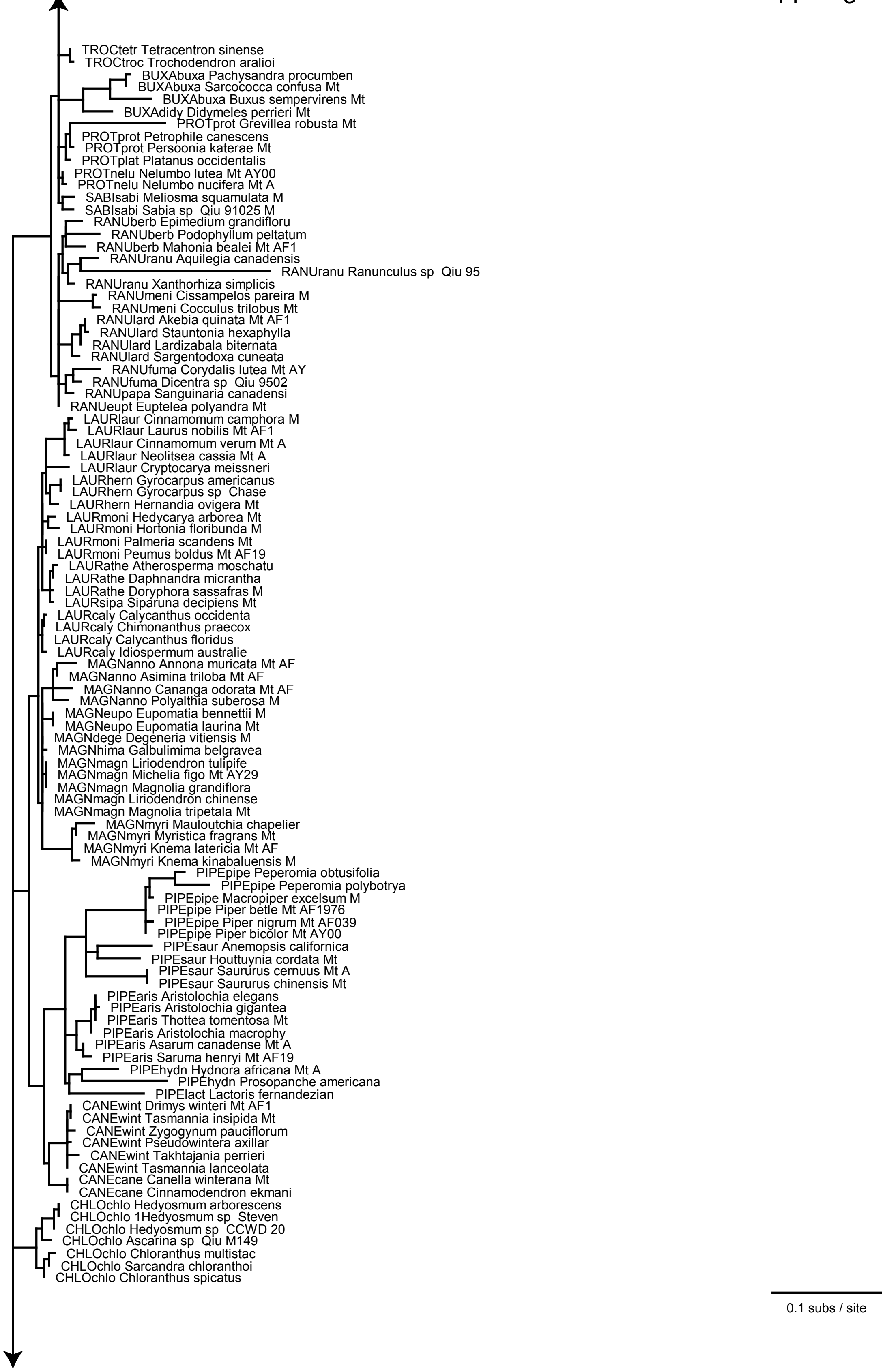
TROCtetr Tetracentron sinense
TROCtroc Trochodendron aralioi
BUXAbuxa Pachysandra procumben
BUXAbuxa Sarcococca confusa Mt
BUXAbuxa Buxus sempervirens Mt
BUXAdidy Didymeles perrieri Mt
PROTprot Grevillea robusta Mt
PROTprot Petrophile canescens
PROTprot Persoonia katerae Mt
PROTplat Platanus occidentalis
PROTnelu Nelumbo lutea Mt AY00
PROTnelu Nelumbo nucifera Mt A
SABIsabi Meliosma squamulata M
SABIsabi Sabia sp Qiu 91025 M
RANUberb Epimedium grandifloru
RANUberb Podophyllum peltatum
RANUberb Mahonia bealei Mt AF1
RANUranu Aquilegia canadensis
RANUranu Ranunculus sp Qiu 95
RANUranu Xanthorhiza simplicis
RANUmeni Cissampelos pareira M
RANUmeni Cocculus trilobus Mt
RANUlard Akebia quinata Mt AF1
RANUlard Stauntonia hexaphylla
RANUlard Lardizabala biternata
RANUlard Sargentodoxa cuneata
RANUfuma Corydalis lutea Mt AY
RANUfuma Dicentra sp Qiu 9502
RANUpapa Sanguinaria canadensi
RANUeupt Euptelea polyandra Mt
LAURlaur Cinnamomum camphora M
LAURlaur Laurus nobilis Mt AF1
LAURlaur Cinnamomum verum Mt A
LAURlaur Neolitsea cassia Mt A
LAURlaur Cryptocarya meissneri
LAURhern Gyrocarpus americanus
LAURhern Gyrocarpus sp Chase
LAURhern Hernandia ovigera Mt
LAURmoni Hedycarya arborea Mt
LAURmoni Hortonia floribunda M
LAURmoni Palmeria scandens Mt
LAURmoni Peumus boldus Mt AF19
LAURathe Atherosperma moschatu
LAURathe Daphnandra micrantha
LAURathe Doryphora sassafras M
LAURsipa Siparuna decipiens Mt
LAURcaly Calycanthus occidenta
LAURcaly Chimonanthus praecox
LAURcaly Calycanthus floridus
LAURcaly Idiospermum australie
MAGNanno Annona muricata Mt AF
MAGNanno Asimina triloba Mt AF
MAGNanno Cananga odorata Mt AF
MAGNanno Polyalthia suberosa M
MAGNeupo Eupomatia bennettii M
MAGNeupo Eupomatia laurina Mt
MAGNdege Degeneria vitiensis M
MAGNhima Galbulimima belgravea
MAGNmagn Liriodendron tulipife
MAGNmagn Michelia figo Mt AY29
MAGNmagn Magnolia grandiflora
MAGNmagn Liriodendron chinense
MAGNmagn Magnolia tripetala Mt
MAGNmyri Mauloutchia chapelier
MAGNmyri Myristica fragrans Mt
MAGNmyri Knema latericia Mt AF
MAGNmyri Knema kinabaluensis M
PIPEpipe Peperomia obtusifolia
PIPEpipe Peperomia polybotrya
PIPEpipe Macropiper excelsum M
PIPEpipe Piper betle Mt AF1976
PIPEpipe Piper nigrum Mt AF039
PIPEpipe Piper bicolor Mt AY00
PIPEsaur Anemopsis californica
PIPEsaur Houttuynia cordata Mt
PIPEsaur Saururus cernuus Mt A
PIPEsaur Saururus chinensis Mt
PIPEaris Aristolochia elegans
PIPEaris Aristolochia gigantea
PIPEaris Thottea tomentosa Mt
PIPEaris Aristolochia macrophy
PIPEaris Asarum canadense Mt A
PIPEaris Saruma henryi Mt AF19
PIPEhydn Hydnora africana Mt A
PIPEhydn Prosopanche americana
PIPElact Lactoris fernandezian
CANEwint Drimys winteri Mt AF1
CANEwint Tasmannia insipida Mt
CANEwint Zygogynum pauciflorum
CANEwint Pseudowintera axillar
CANEwint Takhtajania perrieri
CANEwint Tasmannia lanceolata
CANEcane Canella winterana Mt
CANEcane Cinnamodendron ekmani
CHLOchlo Hedyosmum arborescens
CHLOchlo 1Hedyosmum sp Steven
CHLOchlo Hedyosmum sp CCWD 20
CHLOchlo Ascarina sp Qiu M149
CHLOchlo Chloranthus multistac
CHLOchlo Sarcandra chloranthoi
CHLOchlo Chloranthus spicatus
0.1 subs / site

Supp. Fig. 4d

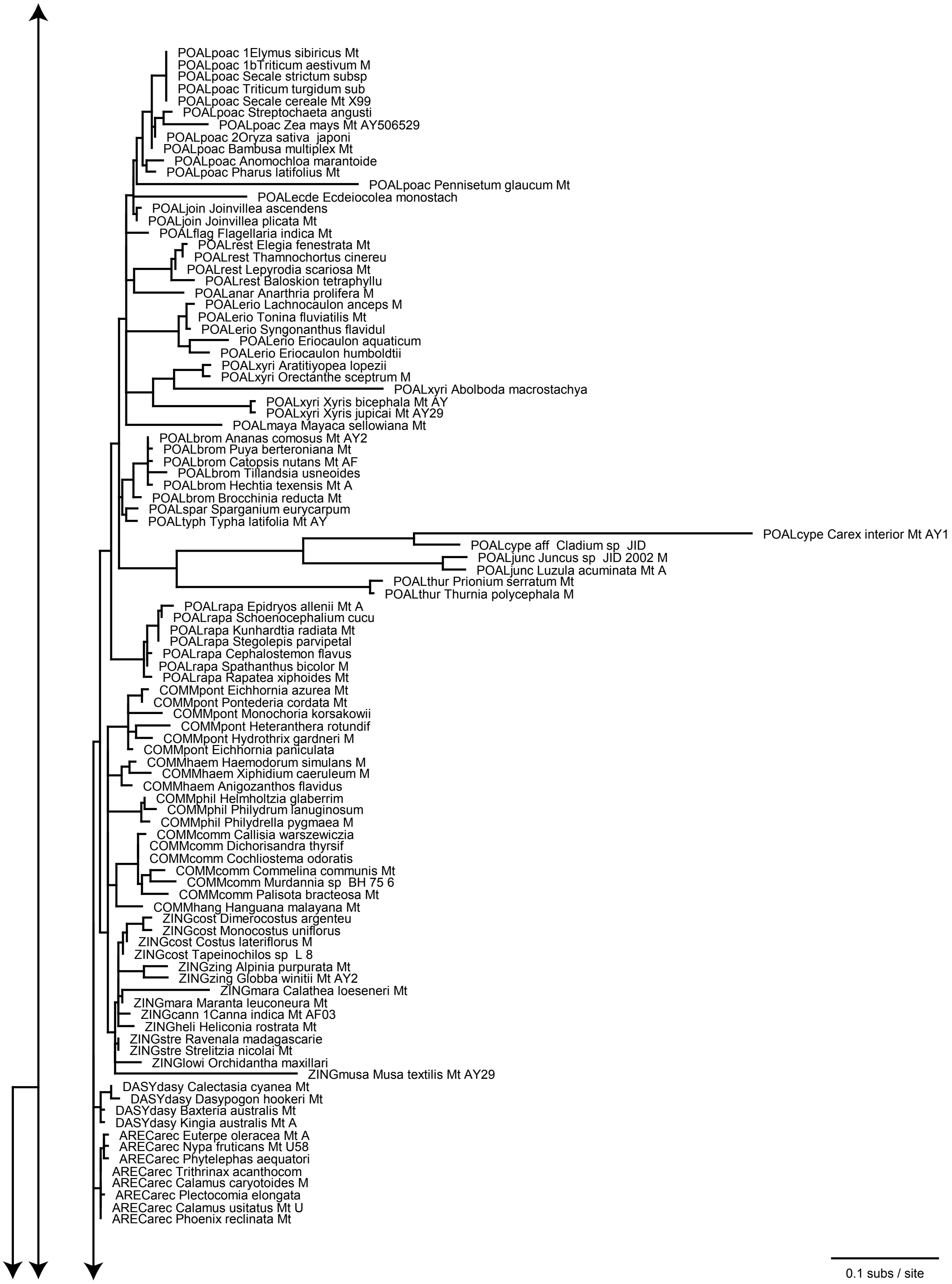
POALpoac 1Elymus sibiricus Mt
POALpoac 1bTriticum aestivum M
POALpoac Secale strictum subsp
POALpoac Triticum turgidum sub
POALpoac Secale cereale Mt X99
POALpoac Streptochaeta angusti
POALpoac Zea mays Mt AY506529
POALpoac 2Oryza sativa japoni
POALpoac Bambusa multiplex Mt
POALpoac Anomochloa marantoide
POALpoac Pharus latifolius Mt
POALpoac Pennisetum glaucum Mt
POALecde Ecdeiocolea monostach
POALjoin Joinvillea ascendens
POALjoin Joinvillea plicata Mt
POALflag Flagellaria indica Mt
POALrest Elegia fenestrata Mt
POALrest Thamnochortus cinereu
POALrest Lepyrodia scariosa Mt
POALrest Baloskion tetraphyllu
POALanar Anarthria prolifera M
POALerio Lachnocaulon anceps M
POALerio Tonina fluviatilis Mt
POALerio Syngonanthus flavidul
POALerio Eriocaulon aquaticum
POALerio Eriocaulon humboldtii
POALxyri Aratitiyopea lopezii
POALxyri Orectanthe sceptrum M
POALxyri Abolboda macrostachya
POALxyri Xyris bicephala Mt AY
POALxyri Xyris jupicai Mt AY29
POALmaya Mayaca sellowiana Mt
POALbrom Ananas comosus Mt AY2
POALbrom Puya berteroniana Mt
POALbrom Catopsis nutans Mt AF
POALbrom Tillandsia usneoides
POALbrom Hechtia texensis Mt A
POALbrom Brocchinia reducta Mt
POALspar Sparganium eurycarpum
POALtyph Typha latifolia Mt AY
POALcype Carex interior Mt AY1
POALcype aff Cladium sp JID
POALjunc Juncus sp JID 2002 M
POALjunc Luzula acuminata Mt A
POALthur Prionium serratum Mt
POALthur Thurnia polycephala M
POALrapa Epidryos allenii Mt A
POALrapa Schoenocephalium cucu
POALrapa Kunhardtia radiata Mt
POALrapa Stegolepis parvipetal
POALrapa Cephalostemon flavus
POALrapa Spathanthus bicolor M
POALrapa Rapatea xiphoides Mt
COMMpont Eichhornia azurea Mt
COMMpont Pontederia cordata Mt
COMMpont Monochoria korsakowii
COMMpont Heteranthera rotundif
COMMpont Hydrothrix gardneri M
COMMpont Eichhornia paniculata
COMMhaem Haemodorum simulans M
COMMhaem Xiphidium caeruleum M
COMMhaem Anigozanthos flavidus
COMMphil Helmholtzia glaberrim
COMMphil Philydrum lanuginosum
COMMphil Philydrella pygmaea M
COMMcomm Callisia warszewiczia
COMMcomm Dichorisandra thyrsif
COMMcomm Cochliostema odoratis
COMMcomm Commelina communis Mt
COMMcomm Murdannia sp BH 75 6
COMMcomm Palisota bracteosa Mt
COMMhang Hanguana malayana Mt
ZINGcost Dimerocostus argenteu
ZINGcost Monocostus uniflorus
ZINGcost Costus lateriflorus M
ZINGcost Tapeinochilos sp L 8
ZINGzing Alpinia purpurata Mt
ZINGzing Globba winitii Mt AY2
ZINGmara Calathea loeseneri Mt
ZINGmara Maranta leuconeura Mt
ZINGcann 1Canna indica Mt AF03
ZINGheli Heliconia rostrata Mt
ZINGstre Ravenala madagascarie
ZINGstre Strelitzia nicolai Mt
ZINGlowi Orchidantha maxillari
ZINGmusa Musa textilis Mt AY29
DASYdasy Calectasia cyanea Mt
DASYdasy Dasypogon hookeri Mt
DASYdasy Baxteria australis Mt
DASYdasy Kingia australis Mt A
ARECarec Euterpe oleracea Mt A
ARECarec Nypa fruticans Mt U58
ARECarec Phytelephas aequatori
ARECarec Trithrinax acanthocom
ARECarec Calamus caryotoides M
ARECarec Plectocomia elongata
ARECarec Calamus usitatus Mt U
ARECarec Phoenix reclinata Mt
0.1 subs / site

Supp. Fig. 4e

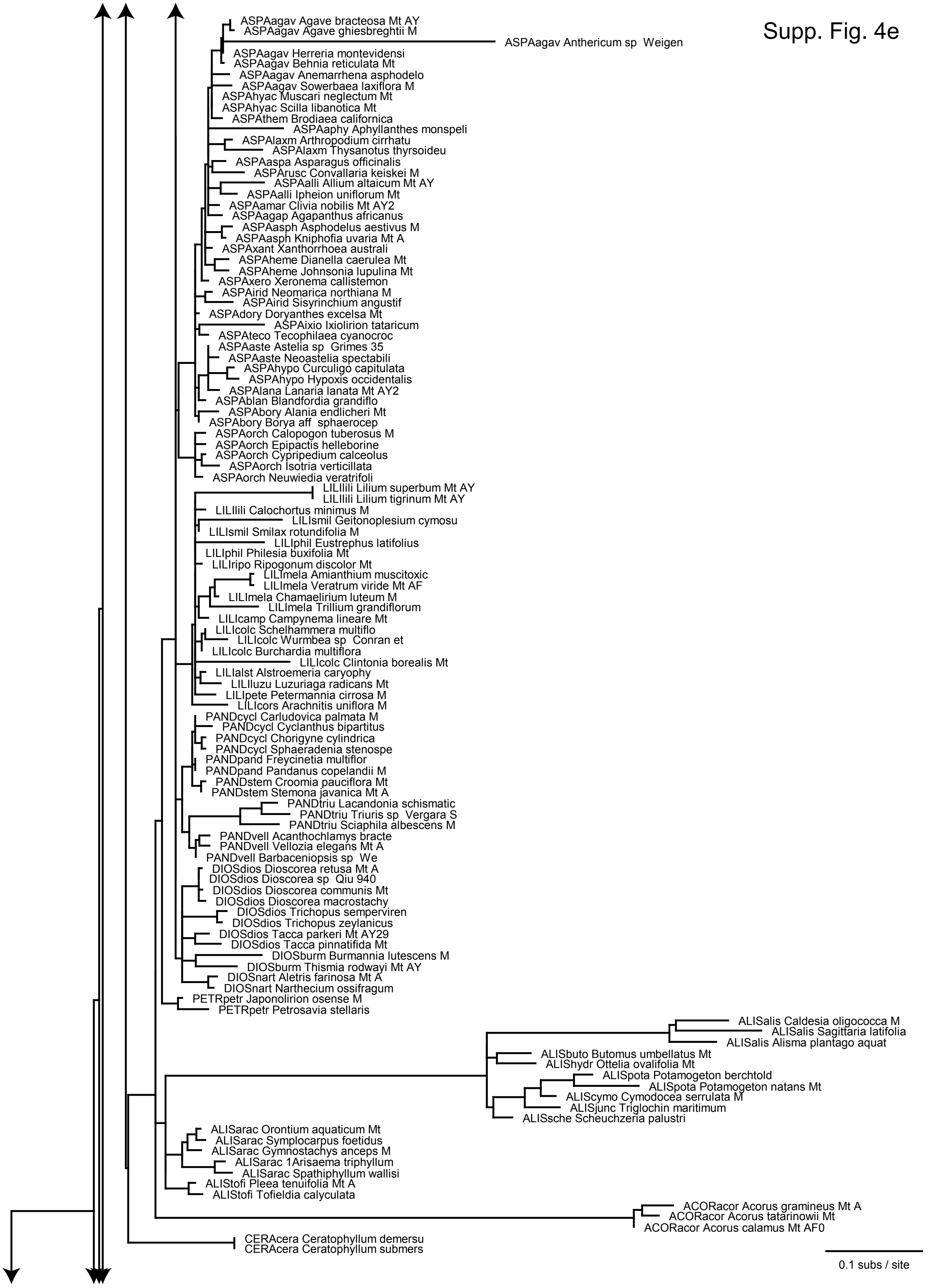

ASPAagav Agave bracteosa Mt AY
ASPAagav Agave ghiesbreghtii M
ASPAagav Anthericum sp Weigen
ASPAagav Herreria montevidensi
ASPAagav Behnia reticulata Mt
ASPAagav Anemarrhena asphodelo
ASPAagav Sowerbaea laxiflora M
ASPAhyac Muscari neglectum Mt
ASPAhyac Scilla libanotica Mt
ASPAthem Brodiaea californica
ASPAaphy Aphyllanthes monspeli
ASPAlaxm Arthropodium cirrhatu
ASPAlaxm Thysanotus thyrsoideu
ASPAaspa Asparagus officinalis
ASPArusc Convallaria keiskei M
ASPAalli Allium altaicum Mt AY
ASPAalli Ipheion uniflorum Mt
ASPAamar Clivia nobilis Mt AY2
ASPAagap Agapanthus africanus
ASPAasph Asphodelus aestivus M
ASPAasph Kniphofia uvaria Mt A
ASPAxant Xanthorrhoea australi
ASPAheme Dianella caerulea Mt
ASPAheme Johnsonia lupulina Mt
ASPAxero Xeronema callistemon
ASPAirid Neomarica northiana M
ASPAirid Sisyrinchium angustif
ASPAdory Doryanthes excelsa Mt
ASPAixio Ixiolirion tataricum
ASPAteco Tecophilaea cyanocroc
ASPAaste Astelia sp Grimes 35
ASPAaste Neoastelia spectabili
ASPAhypo Curculigo capitulata
ASPAhypo Hypoxis occidentalis
ASPAlana Lanaria lanata Mt AY2
ASPAblan Blandfordia grandiflo
ASPAbory Alania endlicheri Mt
ASPAbory Borya aff sphaerocep
ASPAorch Calopogon tuberosus M
ASPAorch Epipactis helleborine
ASPAorch Cypripedium calceolus
ASPAorch Isotria verticillata
ASPAorch Neuwiedia veratrifoli
LILIlili Lilium superbum Mt AY
LILIlili Lilium tigrinum Mt AY
LILIlili Calochortus minimus M
LILIsmil Geitonoplesium cymosu
LILIsmil Smilax rotundifolia M
LILIphil Eustrephus latifolius
LILIphil Philesia buxifolia Mt
LILIripo Ripogonum discolor Mt
LILImela Amianthium muscitoxic
LILImela Veratrum viride Mt AF
LILImela Chamaelirium luteum M
LILImela Trillium grandiflorum
LILIcamp Campynema lineare Mt
LILIcolc Schelhammera multiflo
LILIcolc Wurmbea sp Conran et
LILIcolc Burchardia multiflora
LILIcolc Clintonia borealis Mt
LILIalst Alstroemeria caryophy
LILIluzu Luzuriaga radicans Mt
LILIpete Petermannia cirrosa M
LILIcors Arachnitis uniflora M
PANDcycl Carludovica palmata M
PANDcycl Cyclanthus bipartitus
PANDcycl Chorigyne cylindrica
PANDcycl Sphaeradenia stenospe
PANDpand Freycinetia multiflor
PANDpand Pandanus copelandii M
PANDstem Croomia pauciflora Mt
PANDstem Stemona javanica Mt A
PANDtriu Lacandonia schismatic
PANDtriu Triuris sp Vergara S
PANDtriu Sciaphila albescens M
PANDvell Acanthochlamys bracte
PANDvell Vellozia elegans Mt A
PANDvell Barbaceniopsis sp We
DIOSdios Dioscorea retusa Mt A
DIOSdios Dioscorea sp Qiu 940
DIOSdios Dioscorea communis Mt
DIOSdios Dioscorea macrostachy
DIOSdios Trichopus semperviren
DIOSdios Trichopus zeylanicus
DIOSdios Tacca parkeri Mt AY29
DIOSdios Tacca pinnatifida Mt
DIOSburm Burmannia lutescens M
DIOSburm Thismia rodwayi Mt AY
DIOSnart Aletris farinosa Mt A
DIOSnart Narthecium ossifragum
PETRpetr Japonolirion osense M
PETRpetr Petrosavia stellaris
ALISalis Caldesia oligococca M
ALISalis Sagittaria latifolia
ALISalis Alisma plantago aquat
ALISbuto Butomus umbellatus Mt
ALIShydr Ottelia ovalifolia Mt
ALISpota Potamogeton berchtold
ALISpota Potamogeton natans Mt
ALIScymo Cymodocea serrulata M
ALISjunc Triglochin maritimum
ALISsche Scheuchzeria palustri
ALISarac Orontium aquaticum Mt
ALISarac Symplocarpus foetidus
ALISarac Gymnostachys anceps M
ALISarac 1Arisaema triphyllum
ALISarac Spathiphyllum wallisi
ALIStofi Pleea tenuifolia Mt A
ALIStofi Tofieldia calyculata
ACORacor Acorus gramineus Mt A
ACORacor Acorus tatarinowii Mt
ACORacor Acorus calamus Mt AF0
CERAcera Ceratophyllum demersu
CERAcera Ceratophyllum submers
0.1 subs / site

Supp. Fig. 4f

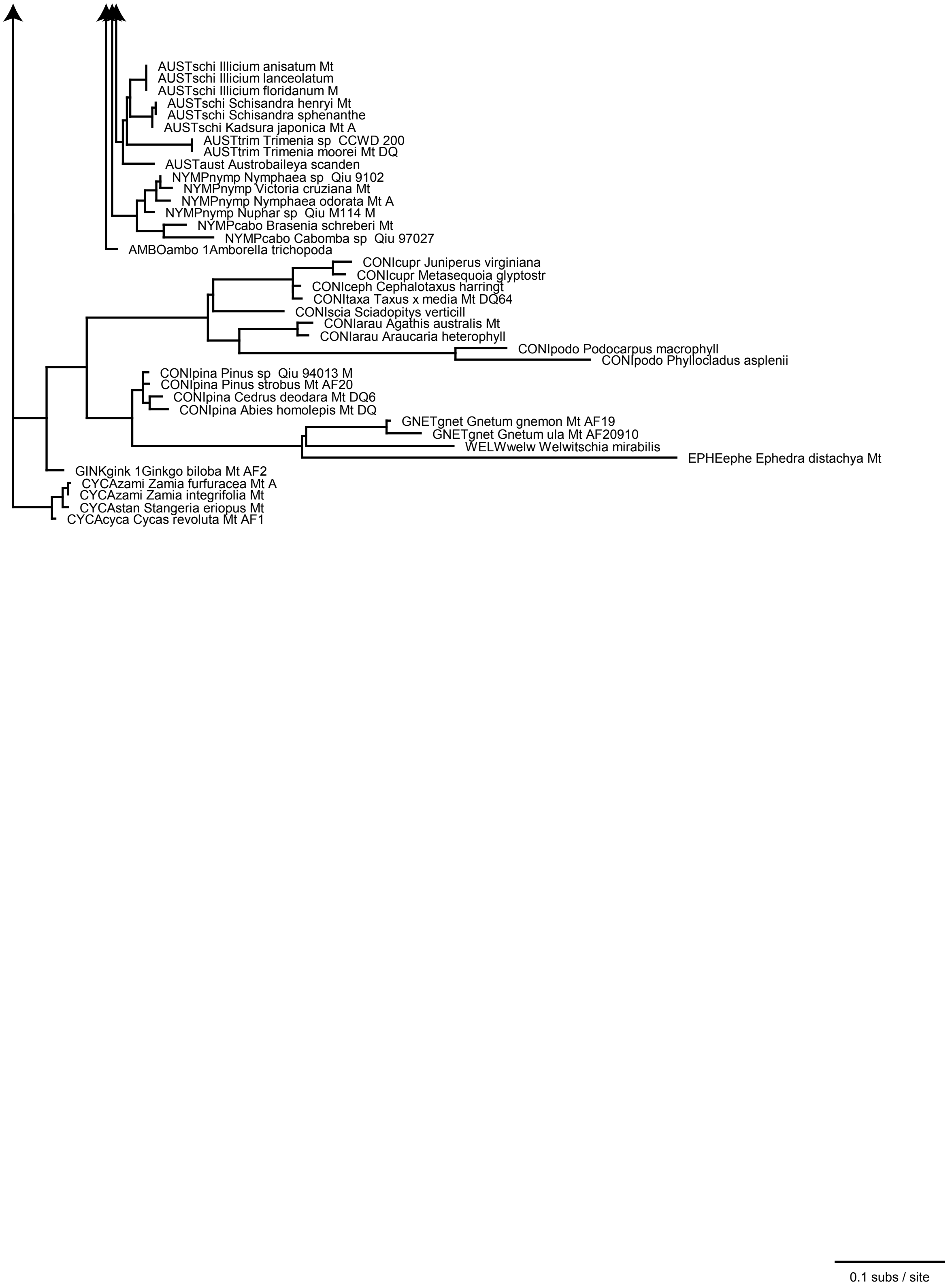
AUSTschi Illicium anisatum Mt
AUSTschi Illicium lanceolatum
AUSTschi Illicium floridanum M
AUSTschi Schisandra henryi Mt
AUSTschi Schisandra sphenanthe
AUSTschi Kadsura japonica Mt A
AUSTtrim Trimenia sp CCWD 200
AUSTtrim Trimenia moorei Mt DQ
AUSTaust Austrobaileya scanden
NYMPnymp Nymphaea sp Qiu 9102
NYMPnymp Victoria cruziana Mt
NYMPnymp Nymphaea odorata Mt A
NYMPnymp Nuphar sp Qiu M114 M
NYMPcabo Brasenia schreberi Mt
NYMPcabo Cabomba sp Qiu 97027
AMBOambo 1Amborella trichopoda
CONIcupr Juniperus virginiana
CONIcupr Metasequoia glyptostr
CONIceph Cephalotaxus harringt
CONItaxa Taxus x media Mt DQ64
CONIscia Sciadopitys verticill
CONIarau Agathis australis Mt
CONIarau Araucaria heterophyll
CONIpodo Podocarpus macrophyll
CONIpodo Phyllocladus asplenii
CONIpina Pinus sp Qiu 94013 M
CONIpina Pinus strobus Mt AF20
CONIpina Cedrus deodara Mt DQ6
CONIpina Abies homolepis Mt DQ
GNETgnet Gnetum gnemon Mt AF19
GNETgnet Gnetum ula Mt AF20910
WELWwelw Welwitschia mirabilis
EPHEephe Ephedra distachya Mt
GINKgink 1Ginkgo biloba Mt AF2
CYCAzami Zamia furfuracea Mt A
CYCAzami Zamia integrifolia Mt
CYCAstan Stangeria eriopus Mt
CYCAcyca Cycas revoluta Mt AF1
0.1 subs / site

Supp. Fig. 5a

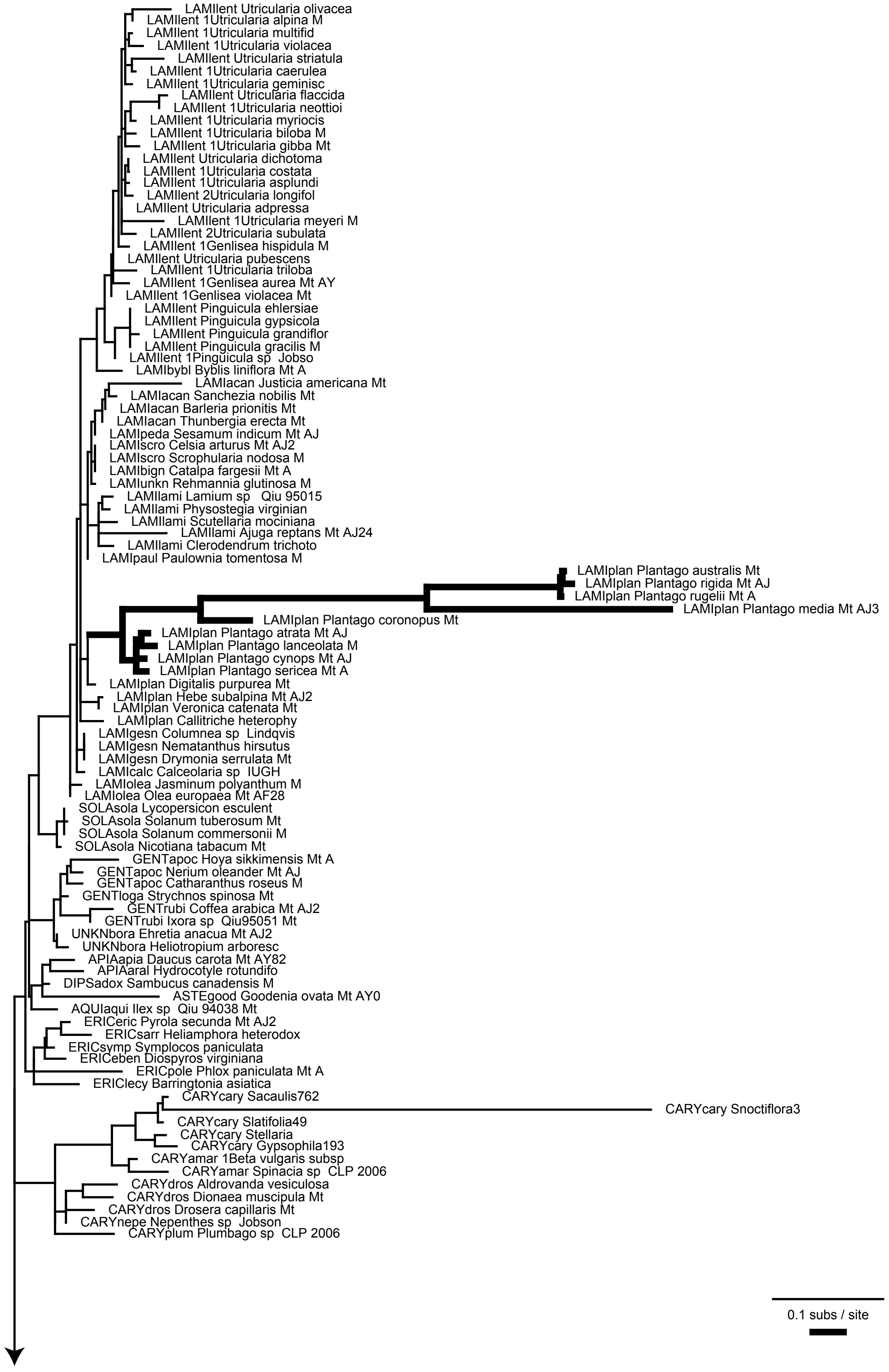

LAMIlent Utricularia olivacea
LAMIlent 1Utricularia alpina M
LAMIlent 1Utricularia multifid
LAMIlent 1Utricularia violacea
LAMIlent Utricularia striatula
LAMIlent 1Utricularia caerulea
LAMIlent 1Utricularia geminisc
LAMIlent Utricularia flaccida
LAMIlent 1Utricularia neottioi
LAMIlent 1Utricularia myriocis
LAMIlent 1Utricularia biloba M
LAMIlent 1Utricularia gibba Mt
LAMIlent Utricularia dichotoma
LAMIlent 1Utricularia costata
LAMIlent 1Utricularia asplundi
LAMIlent 2Utricularia longifol
LAMIlent Utricularia adpressa
LAMIlent 1Utricularia meyeri M
LAMIlent 2Utricularia subulata
LAMIlent 1Genlisea hispidula M
LAMIlent Utricularia pubescens
LAMIlent 1Utricularia triloba
LAMIlent 1Genlisea aurea Mt AY
LAMIlent 1Genlisea violacea Mt
LAMIlent Pinguicula ehlersiae
LAMIlent Pinguicula gypsicola
LAMIlent Pinguicula grandiflor
LAMIlent Pinguicula gracilis M
LAMIlent 1Pinguicula sp Jobso
LAMIbybl Byblis liniflora Mt A
LAMIacan Justicia americana Mt
LAMIacan Sanchezia nobilis Mt
LAMIacan Barleria prionitis Mt
LAMIacan Thunbergia erecta Mt
LAMIpeda Sesamum indicum Mt AJ
LAMIscro Celsia arturus Mt AJ2
LAMIscro Scrophularia nodosa M
LAMIbign Catalpa fargesii Mt A
LAMIunkn Rehmannia glutinosa M
LAMIlami Lamium sp Qiu 95015
LAMIlami Physostegia virginian
LAMIlami Scutellaria mociniana
LAMIlami Ajuga reptans Mt AJ24
LAMIlami Clerodendrum trichoto
LAMIpaul Paulownia tomentosa M
LAMIplan Plantago australis Mt
LAMIplan Plantago rigida Mt AJ
LAMIplan Plantago rugelii Mt A
LAMIplan Plantago media Mt AJ3
LAMIplan Plantago coronopus Mt
LAMIplan Plantago atrata Mt AJ
LAMIplan Plantago lanceolata M
LAMIplan Plantago cynops Mt AJ
LAMIplan Plantago sericea Mt A
LAMIplan Digitalis purpurea Mt
LAMIplan Hebe subalpina Mt AJ2
LAMIplan Veronica catenata Mt
LAMIplan Callitriche heterophy
LAMIgesn Columnea sp Lindqvis
LAMIgesn Nematanthus hirsutus
LAMIgesn Drymonia serrulata Mt
LAMIcalc Calceolaria sp IUGH
LAMIolea Jasminum polyanthum M
LAMIolea Olea europaea Mt AF28
SOLAsola Lycopersicon esculent
SOLAsola Solanum tuberosum Mt
SOLAsola Solanum commersonii M
SOLAsola Nicotiana tabacum Mt
GENTapoc Hoya sikkimensis Mt A
GENTapoc Nerium oleander Mt AJ
GENTapoc Catharanthus roseus M
GENTloga Strychnos spinosa Mt
GENTrubi Coffea arabica Mt AJ2
GENTrubi Ixora sp Qiu95051 Mt
UNKNbora Ehretia anacua Mt AJ2
UNKNbora Heliotropium arboresc
APIAapia Daucus carota Mt AY82
APIAaral Hydrocotyle rotundifo
DIPSadox Sambucus canadensis M
ASTEgood Goodenia ovata Mt AY0
AQUIaqui Ilex sp Qiu 94038 Mt
ERICeric Pyrola secunda Mt AJ2
ERICsarr Heliamphora heterodox
ERICsymp Symplocos paniculata
ERICeben Diospyros virginiana
ERICpole Phlox paniculata Mt A
ERIClecy Barringtonia asiatica
CARYcary Sacaulis762
CARYcary Snoctiflora3
CARYcary Slatifolia49
CARYcary Stellaria
CARYcary Gypsophila193
CARYamar 1Beta vulgaris subsp
CARYamar Spinacia sp CLP 2006
CARYdros Aldrovanda vesiculosa
CARYdros Dionaea muscipula Mt
CARYdros Drosera capillaris Mt
CARYnepe Nepenthes sp Jobson
CARYplum Plumbago sp CLP 2006
0.1 subs / site

Supp. Fig. 5b

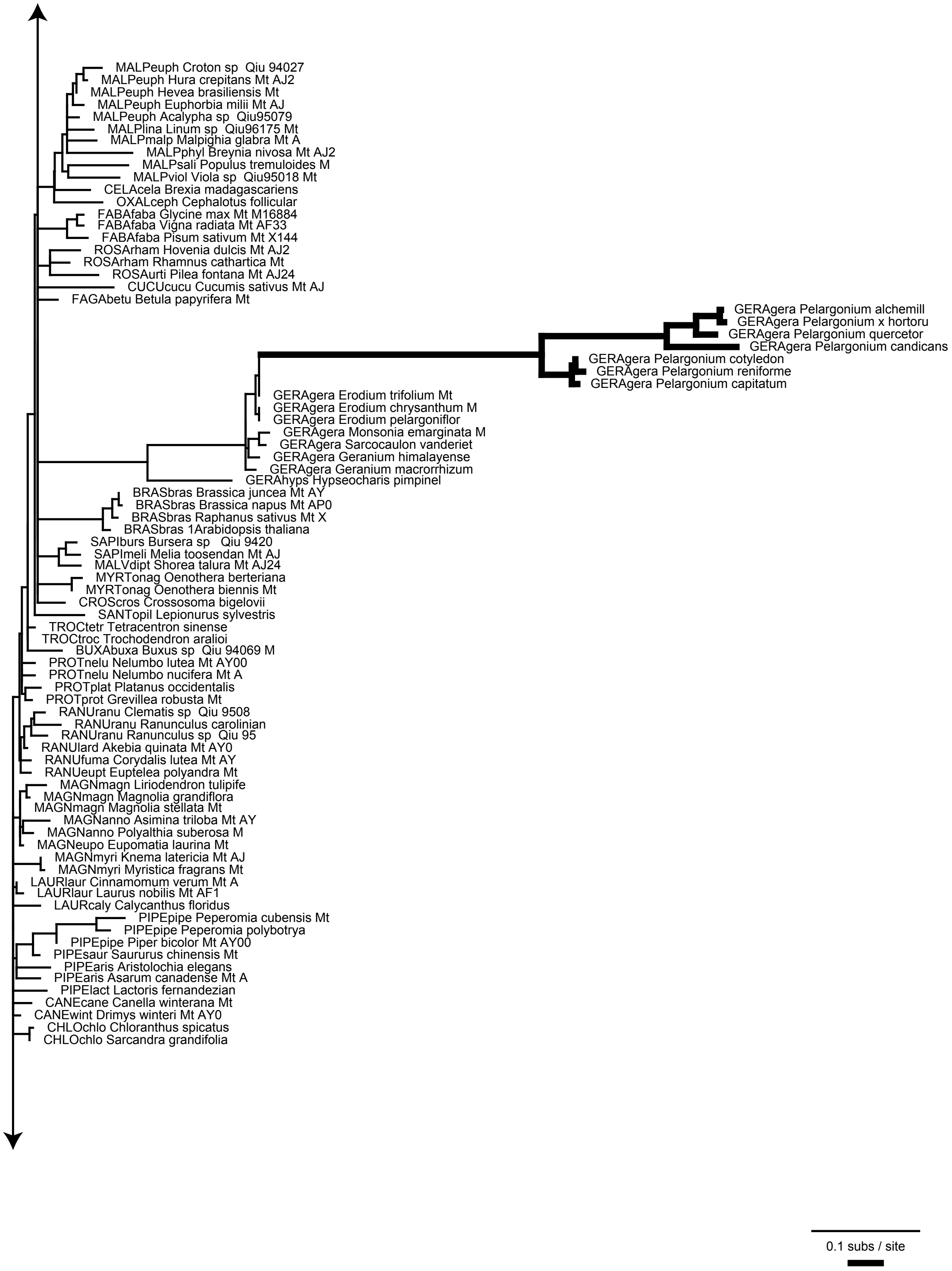
MALPeuph Croton sp Qiu 94027
MALPeuph Hura crepitans Mt AJ2
MALPeuph Hevea brasiliensis Mt
MALPeuph Euphorbia milii Mt AJ
MALPeuph Acalypha sp Qiu95079
MALPlina Linum sp Qiu96175 Mt
MALPmalp Malpighia glabra Mt A
MALPphyl Breynia nivosa Mt AJ2
MALPsali Populus tremuloides M
MALPviol Viola sp Qiu95018 Mt
CELAcela Brexia madagascariens
OXALceph Cephalotus follicular
FABAfaba Glycine max Mt M16884
FABAfaba Vigna radiata Mt AF33
FABAfaba Pisum sativum Mt X144
ROSArham Hovenia dulcis Mt AJ2
ROSArham Rhamnus cathartica Mt
ROSAurti Pilea fontana Mt AJ24
CUCUcucu Cucumis sativus Mt AJ
FAGAbetu Betula papyrifera Mt
GERAgera Pelargonium alchemill
GERAgera Pelargonium x hortoru
GERAgera Pelargonium quercetor
GERAgera Pelargonium candicans
GERAgera Pelargonium cotyledon
GERAgera Pelargonium reniforme
GERAgera Pelargonium capitatum
GERAgera Erodium trifolium Mt
GERAgera Erodium chrysanthum M
GERAgera Erodium pelargoniflor
GERAgera Monsonia emarginata M
GERAgera Sarcocaulon vanderiet
GERAgera Geranium himalayense
GERAgera Geranium macrorrhizum
GERAhyps Hypseocharis pimpinel
BRASbras Brassica juncea Mt AY
BRASbras Brassica napus Mt AP0
BRASbras Raphanus sativus Mt X
BRASbras 1Arabidopsis thaliana
SAPIburs Bursera sp Qiu 9420
SAPImeli Melia toosendan Mt AJ
MALVdipt Shorea talura Mt AJ24
MYRTonag Oenothera berteriana
MYRTonag Oenothera biennis Mt
CROScros Crossosoma bigelovii
SANTopil Lepionurus sylvestris
TROCtetr Tetracentron sinense
TROCtroc Trochodendron aralioi
BUXAbuxa Buxus sp Qiu 94069 M
PROTnelu Nelumbo lutea Mt AY00
PROTnelu Nelumbo nucifera Mt A
PROTplat Platanus occidentalis
PROTprot Grevillea robusta Mt
RANUranu Clematis sp Qiu 9508
RANUranu Ranunculus carolinian
RANUranu Ranunculus sp Qiu 95
RANUlard Akebia quinata Mt AY0
RANUfuma Corydalis lutea Mt AY
RANUeupt Euptelea polyandra Mt
MAGNmagn Liriodendron tulipife
MAGNmagn Magnolia grandiflora
MAGNmagn Magnolia stellata Mt
MAGNanno Asimina triloba Mt AY
MAGNanno Polyalthia suberosa M
MAGNeupo Eupomatia laurina Mt
MAGNmyri Knema latericia Mt AJ
MAGNmyri Myristica fragrans Mt
LAURlaur Cinnamomum verum Mt A
LAURlaur Laurus nobilis Mt AF1
LAURcaly Calycanthus floridus
PIPEpipe Peperomia cubensis Mt
PIPEpipe Peperomia polybotrya
PIPEpipe Piper bicolor Mt AY00
PIPEsaur Saururus chinensis Mt
PIPEaris Aristolochia elegans
PIPEaris Asarum canadense Mt A
PIPElact Lactoris fernandezian
CANEcane Canella winterana Mt
CANEwint Drimys winteri Mt AY0
CHLOchlo Chloranthus spicatus
CHLOchlo Sarcandra grandifolia
0.1 subs / site

Supp. Fig. 5c

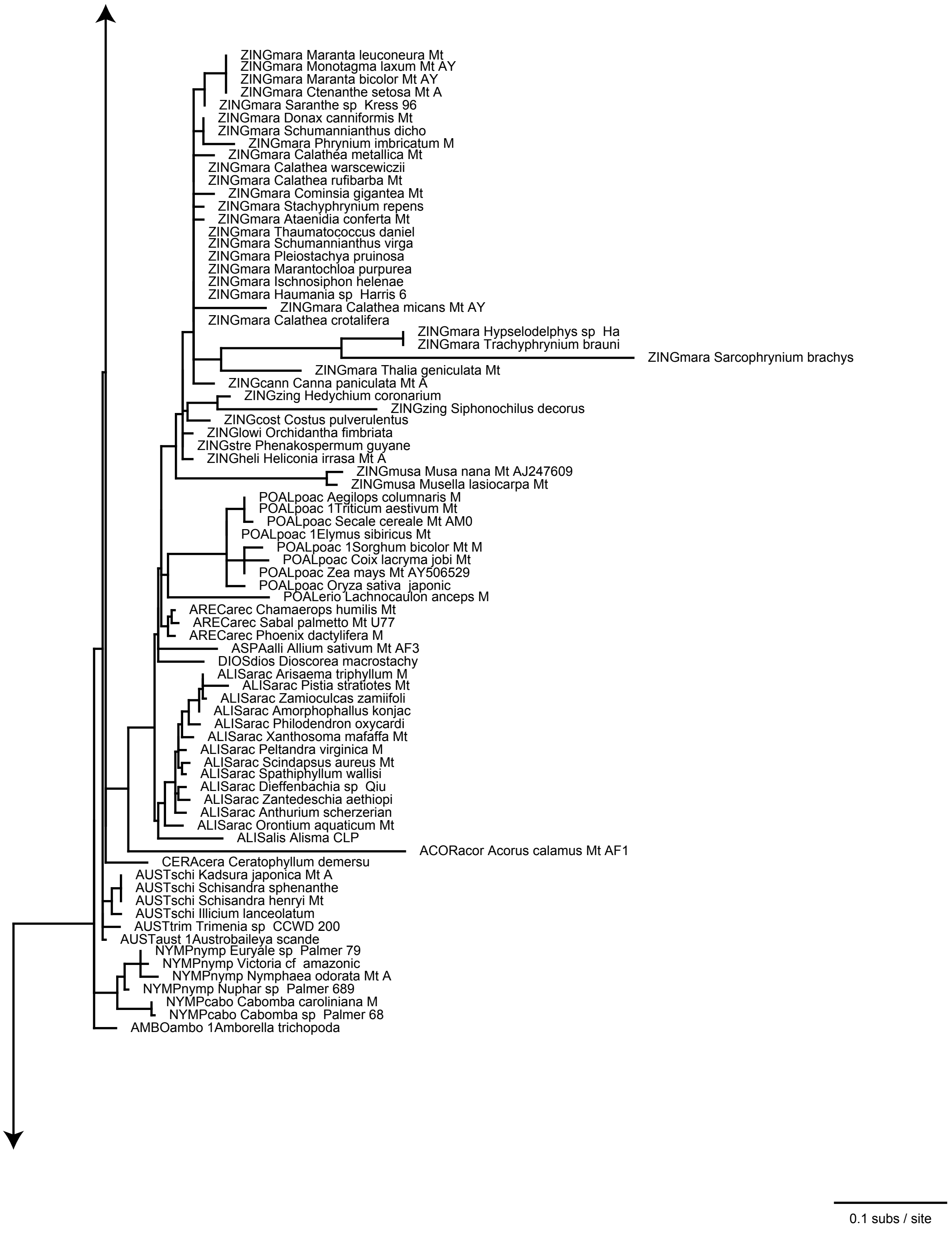
ZINGmara Maranta leuconeura Mt
ZINGmara Monotagma laxum Mt AY
ZINGmara Maranta bicolor Mt AY
ZINGmara Ctenanthe setosa Mt A
ZINGmara Saranthe sp Kress 96
ZINGmara Donax canniformis Mt
ZINGmara Schumannianthus dicho
ZINGmara Phrynium imbricatum M
ZINGmara Calathea metallica Mt
ZINGmara Calathea warscewiczii
ZINGmara Calathea rufibarba Mt
ZINGmara Cominsia gigantea Mt
ZINGmara Stachyphrynium repens
ZINGmara Ataenidia conferta Mt
ZINGmara Thaumatococcus daniel
ZINGmara Schumannianthus virga
ZINGmara Pleiostachya pruinosa
ZINGmara Marantochloa purpurea
ZINGmara Ischnosiphon helenae
ZINGmara Haumania sp Harris 6
ZINGmara Calathea micans Mt AY
ZINGmara Calathea crotalifera
ZINGmara Hypselodelphys sp Ha
ZINGmara Trachyphrynium brauni
ZINGmara Sarcophrynium brachys
ZINGmara Thalia geniculata Mt
ZINGcann Canna paniculata Mt A
ZINGzing Hedychium coronarium
ZINGzing Siphonochilus decorus
ZINGcost Costus pulverulentus
ZINGlowi Orchidantha fimbriata
ZINGstre Phenakospermum guyane
ZINGheli Heliconia irrasa Mt A
ZINGmusa Musa nana Mt AJ247609
ZINGmusa Musella lasiocarpa Mt
POALpoac Aegilops columnaris M
POALpoac 1Triticum aestivum Mt
POALpoac Secale cereale Mt AM0
POALpoac 1Elymus sibiricus Mt
POALpoac 1Sorghum bicolor Mt M
POALpoac Coix lacryma jobi Mt
POALpoac Zea mays Mt AY506529
POALpoac Oryza sativa japonic
POALerio Lachnocaulon anceps M
ARECarec Chamaerops humilis Mt
ARECarec Sabal palmetto Mt U77
ARECarec Phoenix dactylifera M
ASPAalli Allium sativum Mt AF3
DIOSdios Dioscorea macrostachy
ALISarac Arisaema triphyllum M
ALISarac Pistia stratiotes Mt
ALISarac Zamioculcas zamiifoli
ALISarac Amorphophallus konjac
ALISarac Philodendron oxycardi
ALISarac Xanthosoma mafaffa Mt
ALISarac Peltandra virginica M
ALISarac Scindapsus aureus Mt
ALISarac Spathiphyllum wallisi
ALISarac Dieffenbachia sp Qiu
ALISarac Zantedeschia aethiopi
ALISarac Anthurium scherzerian
ALISarac Orontium aquaticum Mt
ALISalis Alisma CLP
ACORacor Acorus calamus Mt AF1
CERAcera Ceratophyllum demersu
AUSTschi Kadsura japonica Mt A
AUSTschi Schisandra sphenanthe
AUSTschi Schisandra henryi Mt
AUSTschi Illicium lanceolatum
AUSTtrim Trimenia sp CCWD 200
AUSTaust 1Austrobaileya scande
NYMPnymp Euryale sp Palmer 79
NYMPnymp Victoria cf amazonic
NYMPnymp Nymphaea odorata Mt A
NYMPnymp Nuphar sp Palmer 689
NYMPcabo Cabomba caroliniana M
NYMPcabo Cabomba sp Palmer 68
AMBOambo 1Amborella trichopoda
0.1 subs / site

Supp. Fig. 5d

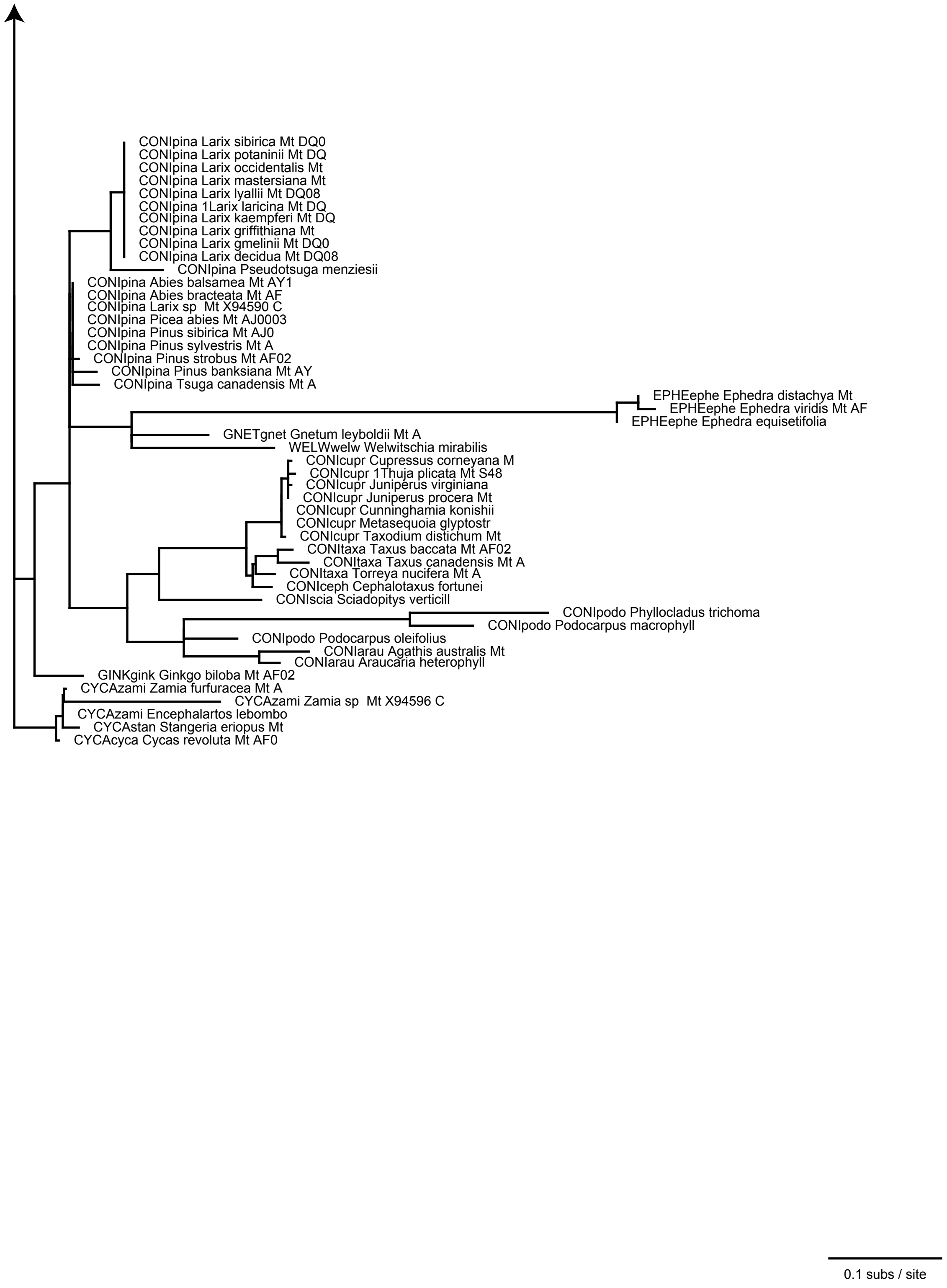
CONIpina Larix sibirica Mt DQ0
CONIpina Larix potaninii Mt DQ
CONIpina Larix occidentalis Mt
CONIpina Larix mastersiana Mt
CONIpina Larix lyallii Mt DQ08
CONIpina 1Larix laricina Mt DQ
CONIpina Larix kaempferi Mt DQ
CONIpina Larix griffithiana Mt
CONIpina Larix gmelinii Mt DQ0
CONIpina Larix decidua Mt DQ08
CONIpina Pseudotsuga menziesii
CONIpina Abies balsamea Mt AY1
CONIpina Abies bracteata Mt AF
CONIpina Larix sp Mt X94590 C
CONIpina Picea abies Mt AJ0003
CONIpina Pinus sibirica Mt AJ0
CONIpina Pinus sylvestris Mt A
CONIpina Pinus strobus Mt AF02
CONIpina Pinus banksiana Mt AY
CONIpina Tsuga canadensis Mt A
EPHEephe Ephedra distachya Mt
EPHEephe Ephedra viridis Mt AF
EPHEephe Ephedra equisetifolia
GNETgnet Gnetum leyboldii Mt A
WELWwelw Welwitschia mirabilis
CONIcupr Cupressus corneyana M
CONIcupr 1Thuja plicata Mt S48
CONIcupr Juniperus virginiana
CONIcupr Juniperus procera Mt
CONIcupr Cunninghamia konishii
CONIcupr Metasequoia glyptostr
CONIcupr Taxodium distichum Mt
CONItaxa Taxus baccata Mt AF02
CONItaxa Taxus canadensis Mt A
CONItaxa Torreya nucifera Mt A
CONIceph Cephalotaxus fortunei
CONIscia Sciadopitys verticill
CONIpodo Phyllocladus trichoma
CONIpodo Podocarpus macrophyll
CONIpodo Podocarpus oleifolius
CONIarau Agathis australis Mt
CONIarau Araucaria heterophyll
GINKgink Ginkgo biloba Mt AF02
CYCAzami Zamia furfuracea Mt A
CYCAzami Zamia sp Mt X94596 C
CYCAzami Encephalartos lebombo
CYCAstan Stangeria eriopus Mt
CYCAcyca Cycas revoluta Mt AF0
0.1 subs / site

Supp. Fig. 6a

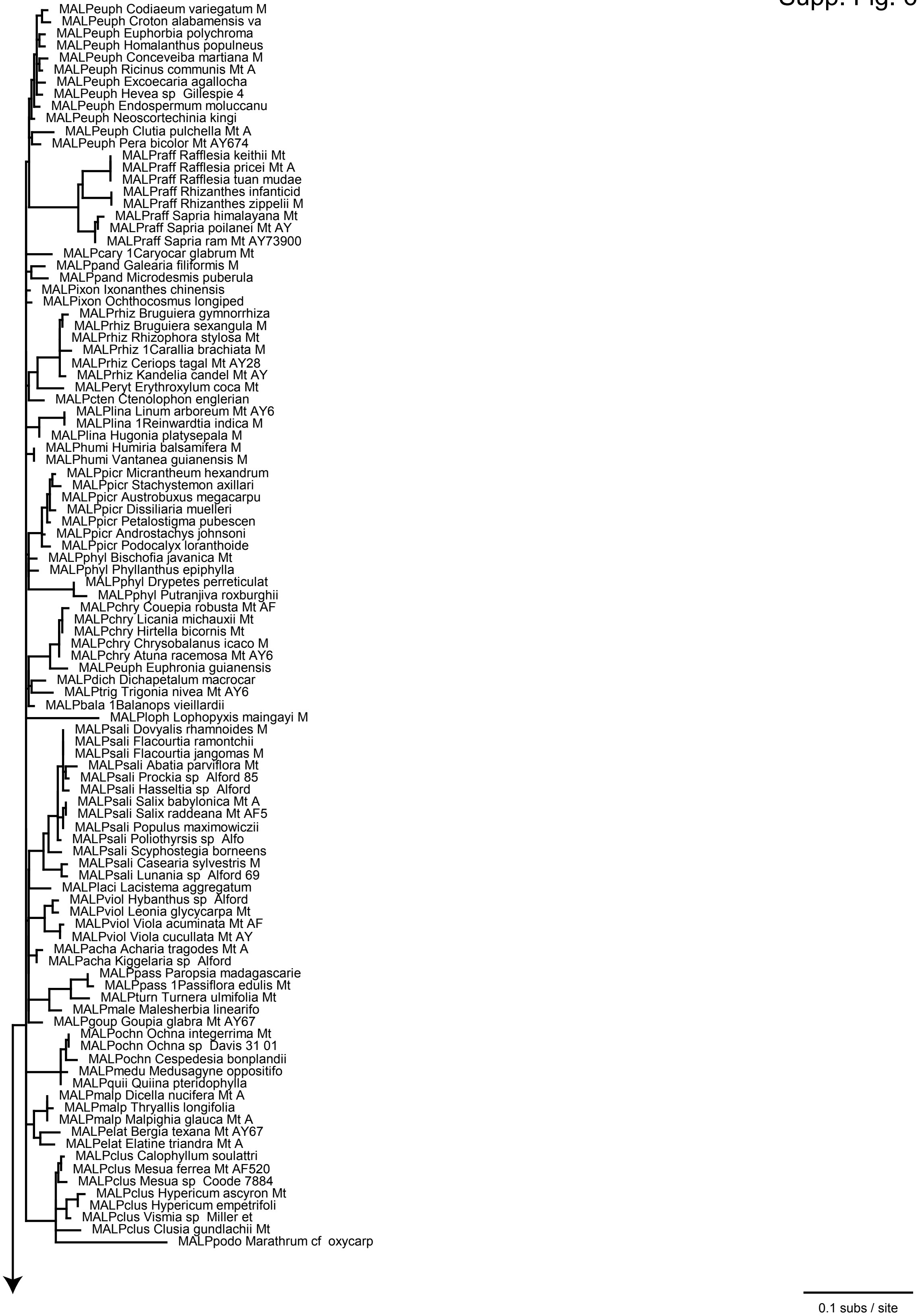
MALPeuph Codiaeum variegatum M
MALPeuph Croton alabamensis va
MALPeuph Euphorbia polychroma
MALPeuph Homalanthus populneus
MALPeuph Conceveiba martiana M
MALPeuph Ricinus communis Mt A
MALPeuph Excoecaria agallocha
MALPeuph Hevea sp Gillespie 4
MALPeuph Endospermum moluccanu
MALPeuph Neoscortechinia kingi
MALPeuph Clutia pulchella Mt A
MALPeuph Pera bicolor Mt AY674
MALPraff Rafflesia keithii Mt
MALPraff Rafflesia pricei Mt A
MALPraff Rafflesia tuan mudae
MALPraff Rhizanthes infanticid
MALPraff Rhizanthes zippelii M
MALPraff Sapria himalayana Mt
MALPraff Sapria poilanei Mt AY
MALPraff Sapria ram Mt AY73900
MALPcary 1Caryocar glabrum Mt
MALPpand Galearia filiformis M
MALPpand Microdesmis puberula
MALPixon Ixonanthes chinensis
MALPixon Ochthocosmus longiped
MALPrhiz Bruguiera gymnorrhiza
MALPrhiz Bruguiera sexangula M
MALPrhiz Rhizophora stylosa Mt
MALPrhiz 1Carallia brachiata M
MALPrhiz Ceriops tagal Mt AY28
MALPrhiz Kandelia candel Mt AY
MALPeryt Erythroxylum coca Mt
MALPcten Ctenolophon englerian
MALPlina Linum arboreum Mt AY6
MALPlina 1Reinwardtia indica M
MALPlina Hugonia platysepala M
MALPhumi Humiria balsamifera M
MALPhumi Vantanea guianensis M
MALPpicr Micrantheum hexandrum
MALPpicr Stachystemon axillari
MALPpicr Austrobuxus megacarpu
MALPpicr Dissiliaria muelleri
MALPpicr Petalostigma pubescen
MALPpicr Androstachys johnsoni
MALPpicr Podocalyx loranthoide
MALPphyl Bischofia javanica Mt
MALPphyl Phyllanthus epiphylla
MALPphyl Drypetes perreticulat
MALPphyl Putranjiva roxburghii
MALPchry Couepia robusta Mt AF
MALPchry Licania michauxii Mt
MALPchry Hirtella bicornis Mt
MALPchry Chrysobalanus icaco M
MALPchry Atuna racemosa Mt AY6
MALPeuph Euphronia guianensis
MALPdich Dichapetalum macrocar
MALPtrig Trigonia nivea Mt AY6
MALPbala 1Balanops vieillardii
MALPloph Lophopyxis maingayi M
MALPsali Dovyalis rhamnoides M
MALPsali Flacourtia ramontchii
MALPsali Flacourtia jangomas M
MALPsali Abatia parviflora Mt
MALPsali Prockia sp Alford 85
MALPsali Hasseltia sp Alford
MALPsali Salix babylonica Mt A
MALPsali Salix raddeana Mt AF5
MALPsali Populus maximowiczii
MALPsali Poliothyrsis sp Alfo
MALPsali Scyphostegia borneens
MALPsali Casearia sylvestris M
MALPsali Lunania sp Alford 69
MALPlaci Lacistema aggregatum
MALPviol Hybanthus sp Alford
MALPviol Leonia glycycarpa Mt
MALPviol Viola acuminata Mt AF
MALPviol Viola cucullata Mt AY
MALPacha Acharia tragodes Mt A
MALPacha Kiggelaria sp Alford
MALPpass Paropsia madagascarie
MALPpass 1Passiflora edulis Mt
MALPturn Turnera ulmifolia Mt
MALPmale Malesherbia linearifo
MALPgoup Goupia glabra Mt AY67
MALPochn Ochna integerrima Mt
MALPochn Ochna sp Davis 31 01
MALPochn Cespedesia bonplandii
MALPmedu Medusagyne oppositifo
MALPquii Quiina pteridophylla
MALPmalp Dicella nucifera Mt A
MALPmalp Thryallis longifolia
MALPmalp Malpighia glauca Mt A
MALPelat Bergia texana Mt AY67
MALPelat Elatine triandra Mt A
MALPclus Calophyllum soulattri
MALPclus Mesua ferrea Mt AF520
MALPclus Mesua sp Coode 7884
MALPclus Hypericum ascyron Mt
MALPclus Hypericum empetrifoli
MALPclus Vismia sp Miller et
MALPclus Clusia gundlachii Mt
MALPpodo Marathrum cf oxycarp
0.1 subs / site

Supp. Fig. 6b

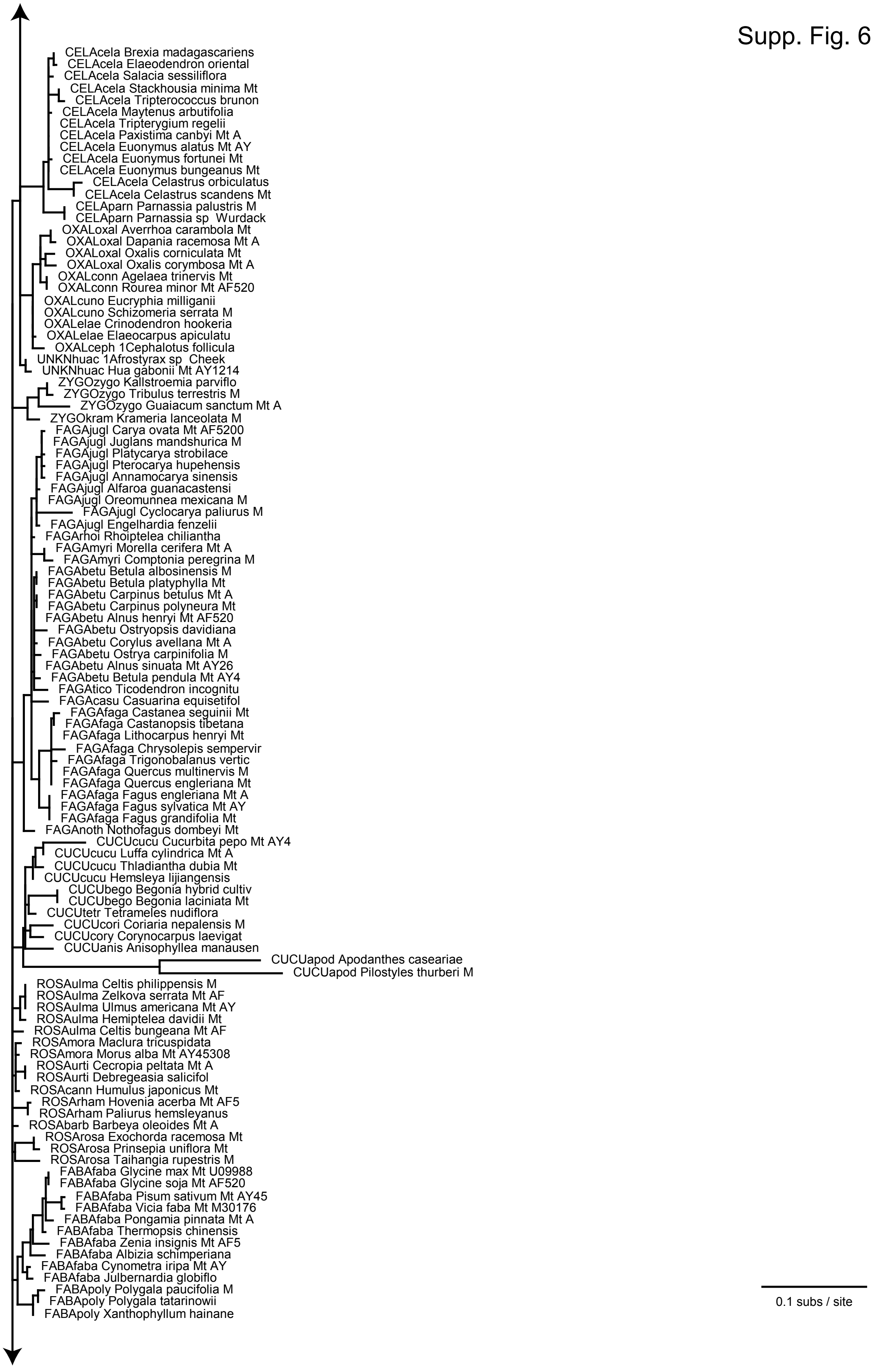
CELAcela Brexia madagascariens
CELAcela Elaeodendron oriental
CELAcela Salacia sessiliflora
CELAcela Stackhousia minima Mt
CELAcela Tripterococcus brunon
CELAcela Maytenus arbutifolia
CELAcela Tripterygium regelii
CELAcela Paxistima canbyi Mt A
CELAcela Euonymus alatus Mt AY
CELAcela Euonymus fortunei Mt
CELAcela Euonymus bungeanus Mt
CELAcela Celastrus orbiculatus
CELAcela Celastrus scandens Mt
CELAparn Parnassia palustris M
CELAparn Parnassia sp Wurdack
OXALoxal Averrhoa carambola Mt
OXALoxal Dapania racemosa Mt A
OXALoxal Oxalis corniculata Mt
OXALoxal Oxalis corymbosa Mt A
OXALconn Agelaea trinervis Mt
OXALconn Rourea minor Mt AF520
OXALcuno Eucryphia milliganii
OXALcuno Schizomeria serrata M
OXALelae Crinodendron hookeria
OXALelae Elaeocarpus apiculatu
OXALceph 1Cephalotus follicula
UNKNhuac 1Afrostyrax sp Cheek
UNKNhuac Hua gabonii Mt AY1214
ZYGOzygo Kallstroemia parviflo
ZYGOzygo Tribulus terrestris M
ZYGOzygo Guaiacum sanctum Mt A
ZYGOkram Krameria lanceolata M
FAGAjugl Carya ovata Mt AF5200
FAGAjugl Juglans mandshurica M
FAGAjugl Platycarya strobilace
FAGAjugl Pterocarya hupehensis
FAGAjugl Annamocarya sinensis
FAGAjugl Alfaroa guanacastensi
FAGAjugl Oreomunnea mexicana M
FAGAjugl Cyclocarya paliurus M
FAGAjugl Engelhardia fenzelii
FAGArhoi Rhoiptelea chiliantha
FAGAmyri Morella cerifera Mt A
FAGAmyri Comptonia peregrina M
FAGAbetu Betula albosinensis M
FAGAbetu Betula platyphylla Mt
FAGAbetu Carpinus betulus Mt A
FAGAbetu Carpinus polyneura Mt
FAGAbetu Alnus henryi Mt AF520
FAGAbetu Ostryopsis davidiana
FAGAbetu Corylus avellana Mt A
FAGAbetu Ostrya carpinifolia M
FAGAbetu Alnus sinuata Mt AY26
FAGAbetu Betula pendula Mt AY4
FAGAtico Ticodendron incognitu
FAGAcasu Casuarina equisetifol
FAGAfaga Castanea seguinii Mt
FAGAfaga Castanopsis tibetana
FAGAfaga Lithocarpus henryi Mt
FAGAfaga Chrysolepis sempervir
FAGAfaga Trigonobalanus vertic
FAGAfaga Quercus multinervis M
FAGAfaga Quercus engleriana Mt
FAGAfaga Fagus engleriana Mt A
FAGAfaga Fagus sylvatica Mt AY
FAGAfaga Fagus grandifolia Mt
FAGAnoth Nothofagus dombeyi Mt
CUCUcucu Cucurbita pepo Mt AY4
CUCUcucu Luffa cylindrica Mt A
CUCUcucu Thladiantha dubia Mt
CUCUcucu Hemsleya lijiangensis
CUCUbego Begonia hybrid cultiv
CUCUbego Begonia laciniata Mt
CUCUtetr Tetrameles nudiflora
CUCUcori Coriaria nepalensis M
CUCUcory Corynocarpus laevigat
CUCUanis Anisophyllea manausen
CUCUapod Apodanthes caseariae
CUCUapod Pilostyles thurberi M
ROSAulma Celtis philippensis M
ROSAulma Zelkova serrata Mt AF
ROSAulma Ulmus americana Mt AY
ROSAulma Hemiptelea davidii Mt
ROSAulma Celtis bungeana Mt AF
ROSAmora Maclura tricuspidata
ROSAmora Morus alba Mt AY45308
ROSAurti Cecropia peltata Mt A
ROSAurti Debregeasia salicifol
ROSAcann Humulus japonicus Mt
ROSArham Hovenia acerba Mt AF5
ROSArham Paliurus hemsleyanus
ROSAbarb Barbeya oleoides Mt A
ROSArosa Exochorda racemosa Mt
ROSArosa Prinsepia uniflora Mt
ROSArosa Taihangia rupestris M
FABAfaba Glycine max Mt U09988
FABAfaba Glycine soja Mt AF520
FABAfaba Pisum sativum Mt AY45
FABAfaba Vicia faba Mt M30176
FABAfaba Pongamia pinnata Mt A
FABAfaba Thermopsis chinensis
FABAfaba Zenia insignis Mt AF5
FABAfaba Albizia schimperiana
FABAfaba Cynometra iripa Mt AY
FABAfaba Julbernardia globiflo
FABApoly Polygala paucifolia M
FABApoly Polygala tatarinowii
FABApoly Xanthophyllum hainane
0.1 subs / site

Supp. Fig. 6c

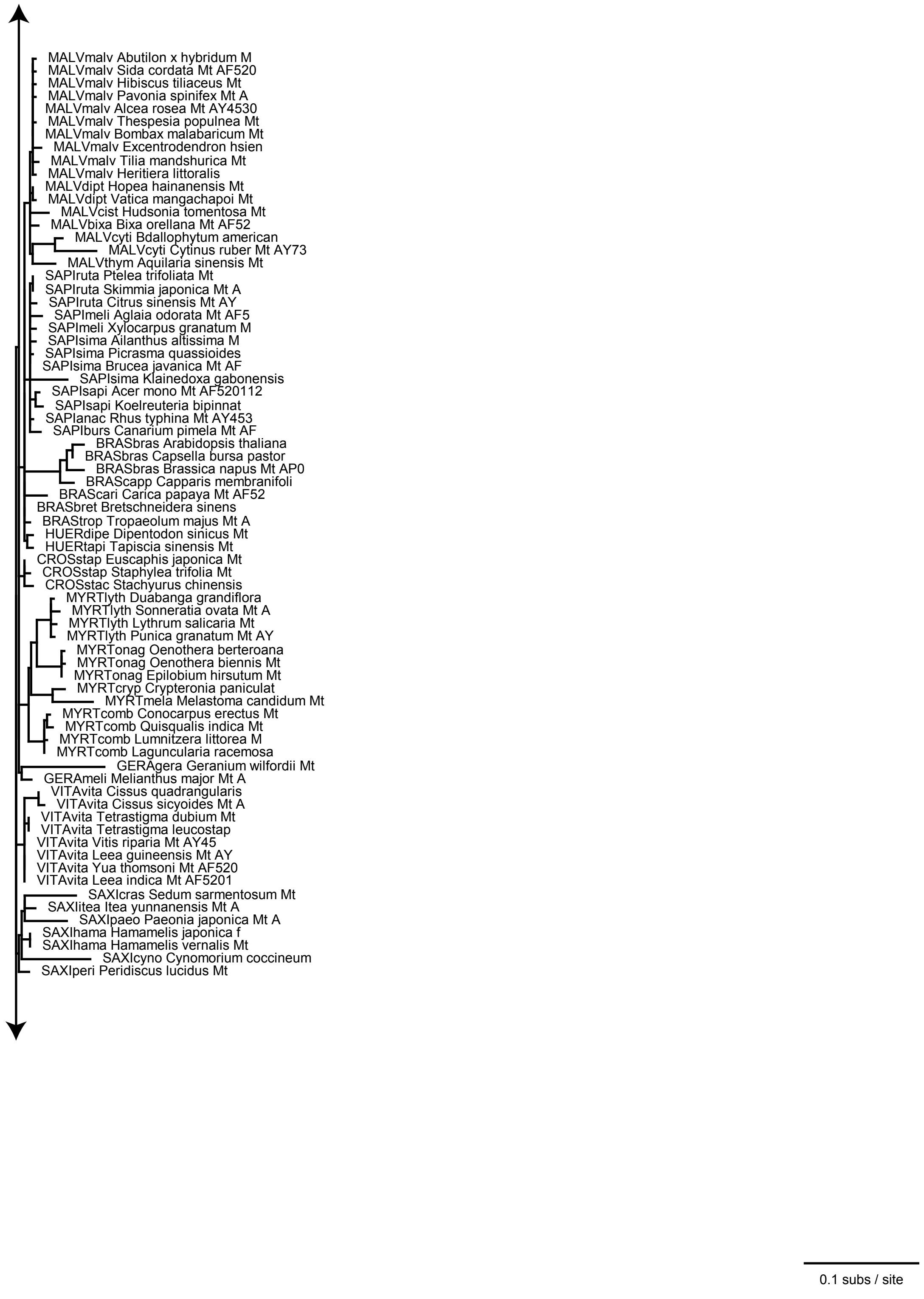
MALVmalv Abutilon x hybridum M
MALVmalv Sida cordata Mt AF520
MALVmalv Hibiscus tiliaceus Mt
MALVmalv Pavonia spinifex Mt A
MALVmalv Alcea rosea Mt AY4530
MALVmalv Thespesia populnea Mt
MALVmalv Bombax malabaricum Mt
MALVmalv Excentrodendron hsien
MALVmalv Tilia mandshurica Mt
MALVmalv Heritiera littoralis
MALVdipt Hopea hainanensis Mt
MALVdipt Vatica mangachapoi Mt
MALVcist Hudsonia tomentosa Mt
MALVbixa Bixa orellana Mt AF52
MALVcyti Bdallophytum american
MALVcyti Cytinus ruber Mt AY73
MALVthym Aquilaria sinensis Mt
SAPIruta Ptelea trifoliata Mt
SAPIruta Skimmia japonica Mt A
SAPIruta Citrus sinensis Mt AY
SAPImeli Aglaia odorata Mt AF5
SAPImeli Xylocarpus granatum M
SAPIsima Ailanthus altissima M
SAPIsima Picrasma quassioides
SAPIsima Brucea javanica Mt AF
SAPIsima Klainedoxa gabonensis
SAPIsapi Acer mono Mt AF520112
SAPIsapi Koelreuteria bipinnat
SAPIanac Rhus typhina Mt AY453
SAPIburs Canarium pimela Mt AF
BRASbras Arabidopsis thaliana
BRASbras Capsella bursa pastor
BRASbras Brassica napus Mt AP0
BRAScapp Capparis membranifoli
BRAScari Carica papaya Mt AF52
BRASbret Bretschneidera sinens
BRAStrop Tropaeolum majus Mt A
HUERdipe Dipentodon sinicus Mt
HUERtapi Tapiscia sinensis Mt
CROSstap Euscaphis japonica Mt
CROSstap Staphylea trifolia Mt
CROSstac Stachyurus chinensis
MYRTlyth Duabanga grandiflora
MYRTlyth Sonneratia ovata Mt A
MYRTlyth Lythrum salicaria Mt
MYRTlyth Punica granatum Mt AY
MYRTonag Oenothera berteroana
MYRTonag Oenothera biennis Mt
MYRTonag Epilobium hirsutum Mt
MYRTcryp Crypteronia paniculat
MYRTmela Melastoma candidum Mt
MYRTcomb Conocarpus erectus Mt
MYRTcomb Quisqualis indica Mt
MYRTcomb Lumnitzera littorea M
MYRTcomb Laguncularia racemosa
GERAgera Geranium wilfordii Mt
GERAmeli Melianthus major Mt A
VITAvita Cissus quadrangularis
VITAvita Cissus sicyoides Mt A
VITAvita Tetrastigma dubium Mt
VITAvita Tetrastigma leucostap
VITAvita Vitis riparia Mt AY45
VITAvita Leea guineensis Mt AY
VITAvita Yua thomsoni Mt AF520
VITAvita Leea indica Mt AF5201
SAXIcras Sedum sarmentosum Mt
SAXIitea Itea yunnanensis Mt A
SAXIpaeo Paeonia japonica Mt A
SAXIhama Hamamelis japonica f
SAXIhama Hamamelis vernalis Mt
SAXIcyno Cynomorium coccineum
SAXIperi Peridiscus lucidus Mt
0.1 subs / site

Supp. Fig. 6d

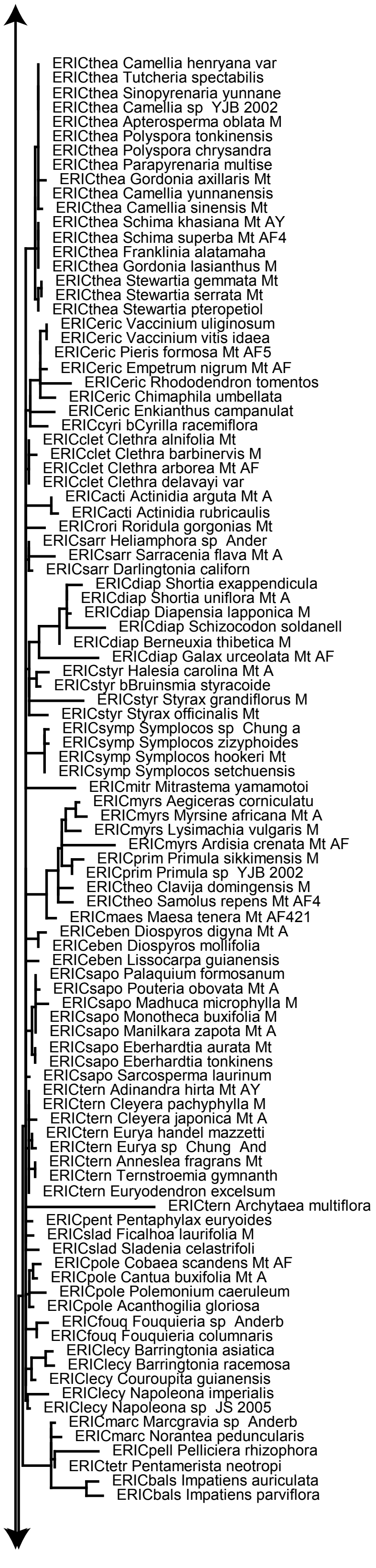
ERICthea Camellia henryana var
ERICthea Tutcheria spectabilis
ERICthea Sinopyrenaria yunnane
ERICthea Camellia sp YJB 2002
ERICthea Apterosperma oblata M
ERICthea Polyspora tonkinensis
ERICthea Polyspora chrysandra
ERICthea Parapyrenaria multise
ERICthea Gordonia axillaris Mt
ERICthea Camellia yunnanensis
ERICthea Camellia sinensis Mt
ERICthea Schima khasiana Mt AY
ERICthea Schima superba Mt AF4
ERICthea Franklinia alatamaha
ERICthea Gordonia lasianthus M
ERICthea Stewartia gemmata Mt
ERICthea Stewartia serrata Mt
ERICthea Stewartia pteropetiol
ERICeric Vaccinium uliginosum
ERICeric Vaccinium vitis idaea
ERICeric Pieris formosa Mt AF5
ERICeric Empetrum nigrum Mt AF
ERICeric Rhododendron tomentos
ERICeric Chimaphila umbellata
ERICeric Enkianthus campanulat
ERICcyri bCyrilla racemiflora
ERICclet Clethra alnifolia Mt
ERICclet Clethra barbinervis M
ERICclet Clethra arborea Mt AF
ERICclet Clethra delavayi var
ERICacti Actinidia arguta Mt A
ERICacti Actinidia rubricaulis
ERICrori Roridula gorgonias Mt
ERICsarr Heliamphora sp Ander
ERICsarr Sarracenia flava Mt A
ERICsarr Darlingtonia californ
ERICdiap Shortia exappendicula
ERICdiap Shortia uniflora Mt A
ERICdiap Diapensia lapponica M
ERICdiap Schizocodon soldanell
ERICdiap Berneuxia thibetica M
ERICdiap Galax urceolata Mt AF
ERICstyr Halesia carolina Mt A
ERICstyr bBruinsmia styracoide
ERICstyr Styrax grandiflorus M
ERICstyr Styrax officinalis Mt
ERICsymp Symplocos sp Chung a
ERICsymp Symplocos zizyphoides
ERICsymp Symplocos hookeri Mt
ERICsymp Symplocos setchuensis
ERICmitr Mitrastema yamamotoi
ERICmyrs Aegiceras corniculatu
ERICmyrs Myrsine africana Mt A
ERICmyrs Lysimachia vulgaris M
ERICmyrs Ardisia crenata Mt AF
ERICprim Primula sikkimensis M
ERICprim Primula sp YJB 2002
ERICtheo Clavija domingensis M
ERICtheo Samolus repens Mt AF4
ERICmaes Maesa tenera Mt AF421
ERICeben Diospyros digyna Mt A
ERICeben Diospyros mollifolia
ERICeben Lissocarpa guianensis
ERICsapo Palaquium formosanum
ERICsapo Pouteria obovata Mt A
ERICsapo Madhuca microphylla M
ERICsapo Monotheca buxifolia M
ERICsapo Manilkara zapota Mt A
ERICsapo Eberhardtia aurata Mt
ERICsapo Eberhardtia tonkinens
ERICsapo Sarcosperma laurinum
ERICtern Adinandra hirta Mt AY
ERICtern Cleyera pachyphylla M
ERICtern Cleyera japonica Mt A
ERICtern Eurya handel mazzetti
ERICtern Eurya sp Chung And
ERICtern Anneslea fragrans Mt
ERICtern Ternstroemia gymnanth
ERICtern Euryodendron excelsum
ERICtern Archytaea multiflora
ERICpent Pentaphylax euryoides
ERICslad Ficalhoa laurifolia M
ERICslad Sladenia celastrifoli
ERICpole Cobaea scandens Mt AF
ERICpole Cantua buxifolia Mt A
ERICpole Polemonium caeruleum
ERICpole Acanthogilia gloriosa
ERICfouq Fouquieria sp Anderb
ERICfouq Fouquieria columnaris
ERIClecy Barringtonia asiatica
ERIClecy Barringtonia racemosa
ERIClecy Couroupita guianensis
ERIClecy Napoleona imperialis
ERIClecy Napoleona sp JS 2005
ERICmarc Marcgravia sp Anderb
ERICmarc Norantea peduncularis
ERICpell Pelliciera rhizophora
ERICtetr Pentamerista neotropi
ERICbals Impatiens auriculata
ERICbals Impatiens parviflora
0.1 subs / site

Supp. Fig. 6e

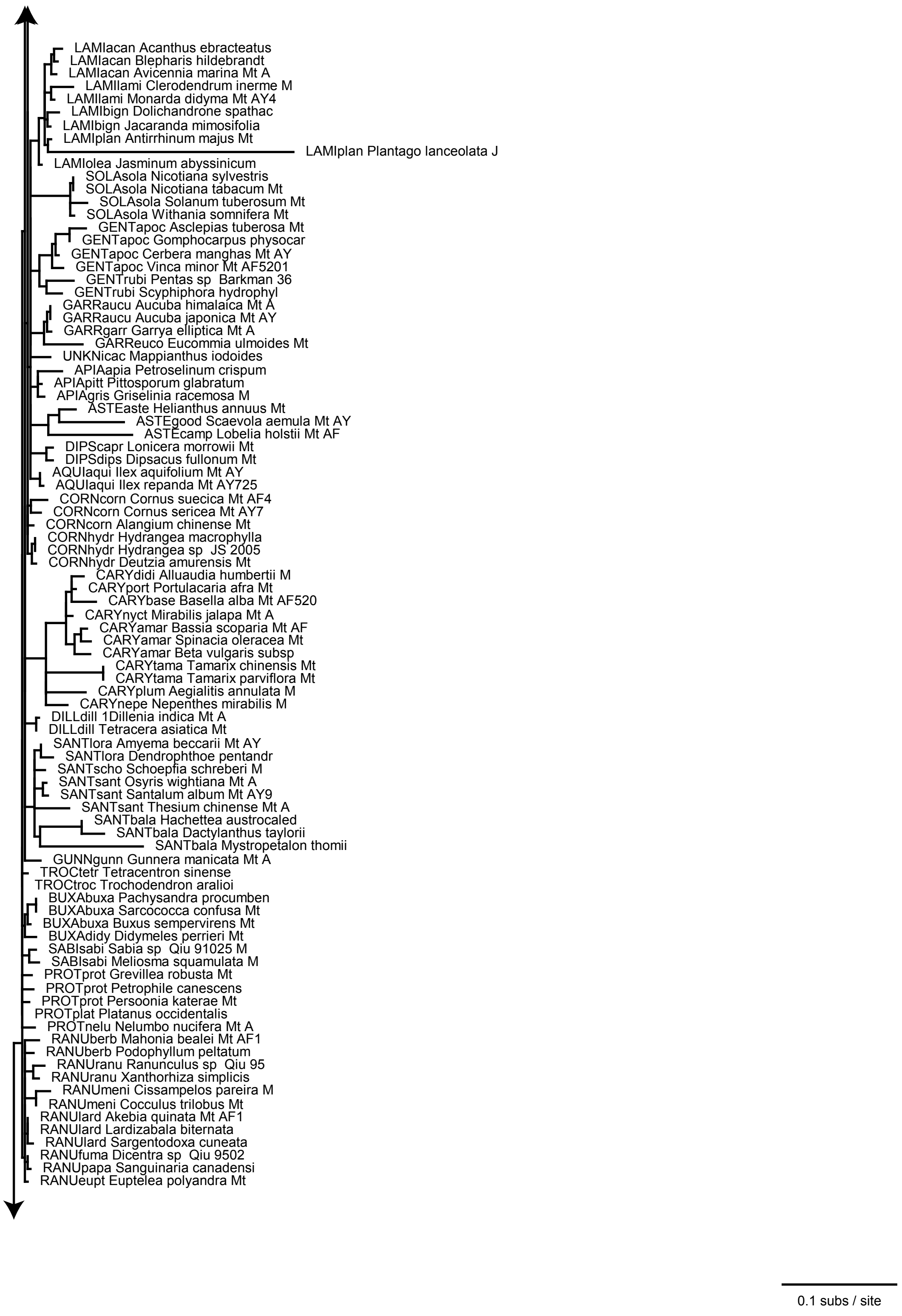
LAMIacan Acanthus ebracteatus
LAMIacan Blepharis hildebrandt
LAMIacan Avicennia marina Mt A
LAMIlami Clerodendrum inerme M
LAMIlami Monarda didyma Mt AY4
LAMIbign Dolichandrone spathac
LAMIbign Jacaranda mimosifolia
LAMIplan Antirrhinum majus Mt
LAMIplan Plantago lanceolata J
LAMIolea Jasminum abyssinicum
SOLAsola Nicotiana sylvestris
SOLAsola Nicotiana tabacum Mt
SOLAsola Solanum tuberosum Mt
SOLAsola Withania somnifera Mt
GENTapoc Asclepias tuberosa Mt
GENTapoc Gomphocarpus physocar
GENTapoc Cerbera manghas Mt AY
GENTapoc Vinca minor Mt AF5201
GENTrubi Pentas sp Barkman 36
GENTrubi Scyphiphora hydrophyl
GARRaucu Aucuba himalaica Mt A
GARRaucu Aucuba japonica Mt AY
GARRgarr Garrya elliptica Mt A
GARReuco Eucommia ulmoides Mt
UNKNicac Mappianthus iodoides
APIAapia Petroselinum crispum
APIApitt Pittosporum glabratum
APIAgris Griselinia racemosa M
ASTEaste Helianthus annuus Mt
ASTEgood Scaevola aemula Mt AY
ASTEcamp Lobelia holstii Mt AF
DIPScapr Lonicera morrowii Mt
DIPSdips Dipsacus fullonum Mt
AQUIaqui Ilex aquifolium Mt AY
AQUIaqui Ilex repanda Mt AY725
CORNcorn Cornus suecica Mt AF4
CORNcorn Cornus sericea Mt AY7
CORNcorn Alangium chinense Mt
CORNhydr Hydrangea macrophylla
CORNhydr Hydrangea sp JS 2005
CORNhydr Deutzia amurensis Mt
CARYdidi Alluaudia humbertii M
CARYport Portulacaria afra Mt
CARYbase Basella alba Mt AF520
CARYnyct Mirabilis jalapa Mt A
CARYamar Bassia scoparia Mt AF
CARYamar Spinacia oleracea Mt
CARYamar Beta vulgaris subsp
CARYtama Tamarix chinensis Mt
CARYtama Tamarix parviflora Mt
CARYplum Aegialitis annulata M
CARYnepe Nepenthes mirabilis M
DILLdill 1Dillenia indica Mt A
DILLdill Tetracera asiatica Mt
SANTlora Amyema beccarii Mt AY
SANTlora Dendrophthoe pentandr
SANTscho Schoepfia schreberi M
SANTsant Osyris wightiana Mt A
SANTsant Santalum album Mt AY9
SANTsant Thesium chinense Mt A
SANTbala Hachettea austrocaled
SANTbala Dactylanthus taylorii
SANTbala Mystropetalon thomii
GUNNgunn Gunnera manicata Mt A
TROCtetr Tetracentron sinense
TROCtroc Trochodendron aralioi
BUXAbuxa Pachysandra procumben
BUXAbuxa Sarcococca confusa Mt
BUXAbuxa Buxus sempervirens Mt
BUXAdidy Didymeles perrieri Mt
SABIsabi Sabia sp Qiu 91025 M
SABIsabi Meliosma squamulata M
PROTprot Grevillea robusta Mt
PROTprot Petrophile canescens
PROTprot Persoonia katerae Mt
PROTplat Platanus occidentalis
PROTnelu Nelumbo nucifera Mt A
RANUberb Mahonia bealei Mt AF1
RANUberb Podophyllum peltatum
RANUranu Ranunculus sp Qiu 95
RANUranu Xanthorhiza simplicis
RANUmeni Cissampelos pareira M
RANUmeni Cocculus trilobus Mt
RANUlard Akebia quinata Mt AF1
RANUlard Lardizabala biternata
RANUlard Sargentodoxa cuneata
RANUfuma Dicentra sp Qiu 9502
RANUpapa Sanguinaria canadensi
RANUeupt Euptelea polyandra Mt
0.1 subs / site

Supp. Fig. 6f

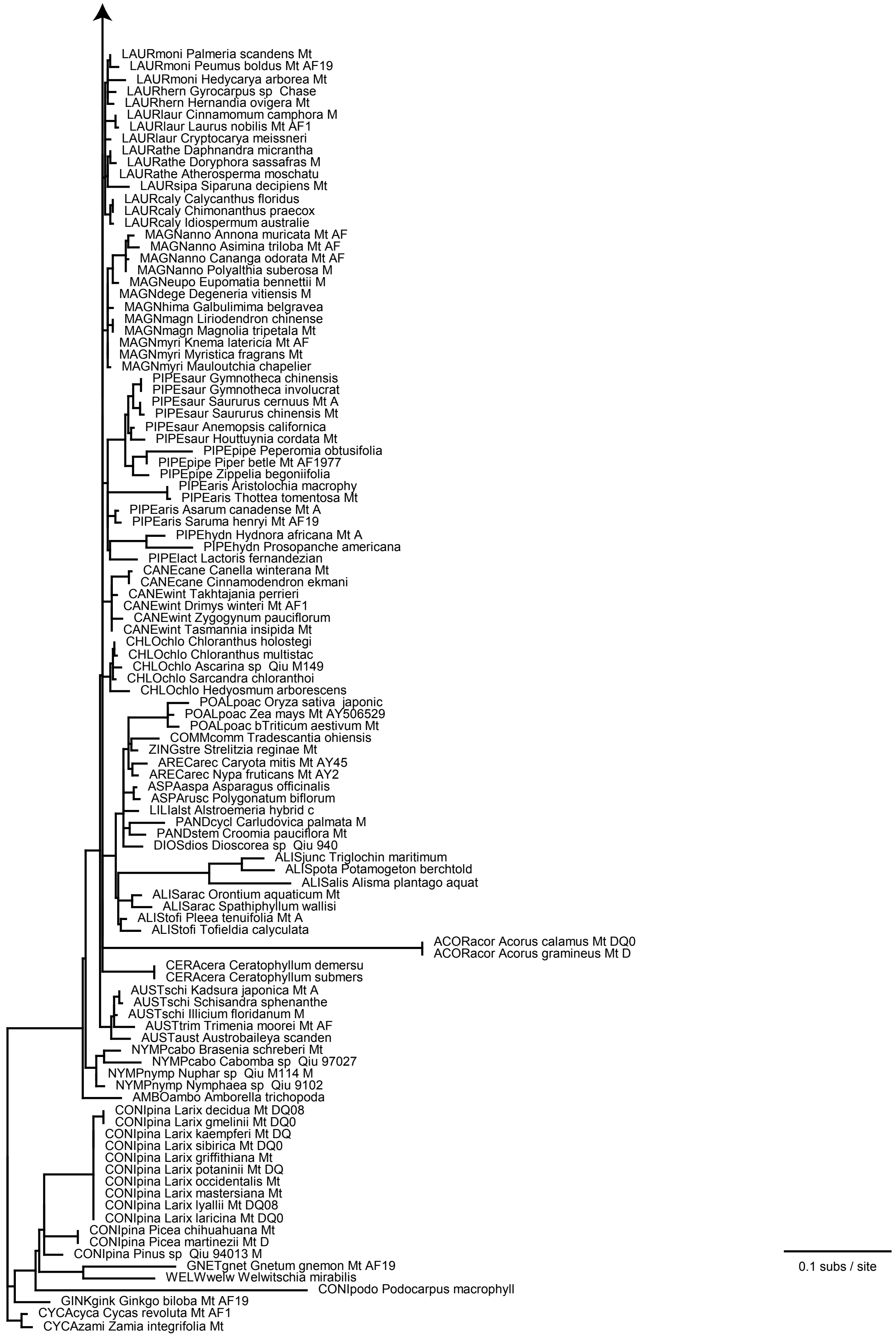
LAURmoni Palmeria scandens Mt
LAURmoni Peumus boldus Mt AF19
LAURmoni Hedycarya arborea Mt
LAURhern Gyrocarpus sp Chase
LAURhern Hernandia ovigera Mt
LAURlaur Cinnamomum camphora M
LAURlaur Laurus nobilis Mt AF1
LAURlaur Cryptocarya meissneri
LAURathe Daphnandra micrantha
LAURathe Doryphora sassafras M
LAURathe Atherosperma moschatu
LAURsipa Siparuna decipiens Mt
LAURcaly Calycanthus floridus
LAURcaly Chimonanthus praecox
LAURcaly Idiospermum australie
MAGNanno Annona muricata Mt AF
MAGNanno Asimina triloba Mt AF
MAGNanno Cananga odorata Mt AF
MAGNanno Polyalthia suberosa M
MAGNeupo Eupomatia bennettii M
MAGNdege Degeneria vitiensis M
MAGNhima Galbulimima belgravea
MAGNmagn Liriodendron chinense
MAGNmagn Magnolia tripetala Mt
MAGNmyri Knema latericia Mt AF
MAGNmyri Myristica fragrans Mt
MAGNmyri Mauloutchia chapelier
PIPEsaur Gymnotheca chinensis
PIPEsaur Gymnotheca involucrat
PIPEsaur Saururus cernuus Mt A
PIPEsaur Saururus chinensis Mt
PIPEsaur Anemopsis californica
PIPEsaur Houttuynia cordata Mt
PIPEpipe Peperomia obtusifolia
PIPEpipe Piper betle Mt AF1977
PIPEpipe Zippelia begoniifolia
PIPEaris Aristolochia macrophy
PIPEaris Thottea tomentosa Mt
PIPEaris Asarum canadense Mt A
PIPEaris Saruma henryi Mt AF19
PIPEhydn Hydnora africana Mt A
PIPEhydn Prosopanche americana
PIPElact Lactoris fernandezian
CANEcane Canella winterana Mt
CANEcane Cinnamodendron ekmani
CANEwint Takhtajania perrieri
CANEwint Drimys winteri Mt AF1
CANEwint Zygogynum pauciflorum
CANEwint Tasmannia insipida Mt
CHLOchlo Chloranthus holostegi
CHLOchlo Chloranthus multistac
CHLOchlo Ascarina sp Qiu M149
CHLOchlo Sarcandra chloranthoi
CHLOchlo Hedyosmum arborescens
POALpoac Oryza sativa japonic
POALpoac Zea mays Mt AY506529
POALpoac bTriticum aestivum Mt
COMMcomm Tradescantia ohiensis
ZINGstre Strelitzia reginae Mt
ARECarec Caryota mitis Mt AY45
ARECarec Nypa fruticans Mt AY2
ASPAaspa Asparagus officinalis
ASPArusc Polygonatum biflorum
LILIalst Alstroemeria hybrid c
PANDcycl Carludovica palmata M
PANDstem Croomia pauciflora Mt
DIOSdios Dioscorea sp Qiu 940
ALISjunc Triglochin maritimum
ALISpota Potamogeton berchtold
ALISalis Alisma plantago aquat
ALISarac Orontium aquaticum Mt
ALISarac Spathiphyllum wallisi
ALIStofi Pleea tenuifolia Mt A
ALIStofi Tofieldia calyculata
ACORacor Acorus calamus Mt DQ0
ACORacor Acorus gramineus Mt D
CERAcera Ceratophyllum demersu
CERAcera Ceratophyllum submers
AUSTschi Kadsura japonica Mt A
AUSTschi Schisandra sphenanthe
AUSTschi Illicium floridanum M
AUSTtrim Trimenia moorei Mt AF
AUSTaust Austrobaileya scanden
NYMPcabo Brasenia schreberi Mt
NYMPcabo Cabomba sp Qiu 97027
NYMPnymp Nuphar sp Qiu M114 M
NYMPnymp Nymphaea sp Qiu 9102
AMBOambo Amborella trichopoda
CONIpina Larix decidua Mt DQ08
CONIpina Larix gmelinii Mt DQ0
CONIpina Larix kaempferi Mt DQ
CONIpina Larix sibirica Mt DQ0
CONIpina Larix griffithiana Mt
CONIpina Larix potaninii Mt DQ
CONIpina Larix occidentalis Mt
CONIpina Larix mastersiana Mt
CONIpina Larix lyallii Mt DQ08
CONIpina Larix laricina Mt DQ0
CONIpina Picea chihuahuana Mt
CONIpina Picea martinezii Mt D
CONIpina Pinus sp Qiu 94013 M
GNETgnet Gnetum gnemon Mt AF19
WELWwelw Welwitschia mirabilis
CONIpodo Podocarpus macrophyll
GINKgink Ginkgo biloba Mt AF19
CYCAcyca Cycas revoluta Mt AF1
CYCAzami Zamia integrifolia Mt
0.1 subs / site
