# Supplementary material for: Extensive variation in synonymous substitution rates in mitochondrial genes of seed plants
Source: BMC Evol Biol. 2007 Aug 9;7:135. doi: 10.1186/1471-2148-7-135 (PMC1973135; doi:10.1186/1471-2148-7-135)
Supplement: Additional file 2 — Supplementary tables. Supplementary Table 1 lists the primers used in this study. Supplementary Tables 2-7 list the taxon names and GenBank accession numbers of all sequence used in this study. [file 1471-2148-7-135-S2.pdf]

Table S1. PCR and sequencing primers used in this study

Forward Primers		Reverse Primers	
atp1-F1	ACACGAATTTTCAAGTGGATGAGA	atp1-R1	ACCTCTATTGAGTAATGCCTGAGT
atp1-F4	TGTCTATGTAGCGRTTGGACAG	atp1-R3	TCTAGTGGCATTCGATCACAGAA
		atp1-R4	GTSGCTGCTACAAGAATGGAAT
cob-UB1	ACTATAAGRAACCAACGRYTM	cob-UB3	GATCCATATTGATGCAATGCG
cob-UB2	AGGCGCCAGTCTTCTTGATCTGG	cob-UB4	AATTCCTCTTCSAACTCGTCC
cob-F1	AGCATTTGATAGATTATCCAACC	cob-R717	GATGCCCCAAAACATTAGGA
cob-F362	TTGGGGTCAGATGAGCTTTT	cob-R1084	ATTCTTCTTCCAACTCGTCC
cox1-UB1	AYGAMAAATCYGGTYCGATGG	cox1-UB4	ACCGRATCCAGGCAGAATGRG
cox1-UB3	CATCTCTTTYTGTTCTTCGGT	cox1-UB6	AGCTGGAAGTTCTCCAAAAGT
cox1-F1	GGAGCAGTTGATTTAGCCAT	cox1-R663	CCCAGAATTTGCCAGGACTA
cox1-F473	TTGATACCCGCGCTTACTTC	cox1-R1077	CCATTCCAGTGTGGGTGAAT
cox2-UB1	TTGTGATGCWGCGGAACCRTG	cox2-UB7	ACHGCTTCTACGACGATAGR
cox2-UB2	TGAGTATTCDGACTATAACAR		
cox3-UB1	ATGATTGAATCTCARAGGCAY	cox3-UB2	TCATATACCTCCCCACCAA
LSU-UB1	GTTAGTAGCGGCGAGCGAGAG	LSU-UB6	TTCGGGTCAAATAGGAAGAAC
LSU-UB2	CACTCACTCTAACGGCGTACC	LSU-UB7	TTCCACTGGCAGGCGATCGTG
LSU-UB3	TGGAGATATCAGAAGTGAGAA	LSU-UB8	TYSTTGACTATGACAASAGTC
LSU-UB4	CCTRCCATAGTCGCGAGKMT	LSU-UB9	AGAGTGGTCTTTCAGGATTGG
matK-F1	GTACTCGGCTTTTAAGTGCG	matK-R1	GCACACGGCTTTCCCTATG
matK-F2	CCTTGTTTTGACTGTATCGCMC	matK-R2	CCAGACCGACTTACTAATAGGATG
matK-F2b	AACTAGAGGGTTCTTGGCAAC	matK-R3	CATCTCTTACCCAGTACCGAAG
matK-F3	GCCCATCTAGAAATCTTGGTTC	matK-R3b	GCGAATAAAAATTGYGTTTGTGC
matK-F3b	AATTTYCATGTATGTGAATACGAATC	matK-R4	TGCGATACAGTCAAAACARGG
matK-F4	CAATTTTGTAACCGATTAGGACATC	matK-R4b	GGTAAATTCATCARCGGACTCTC
SSU-UB1	GAGTTTGATCCTGGCTCAGAA	SSU-UB5	TTCCACTCTCCTCTGTCTCAC
SSU-UB2	AGGGCACGTAGGCGGTGAATC	SSU-UB6	CATGTCTSAGCAACACAARAC
SSU-UB3	TCGTCAGCTCGTGTCGTGAGA	SSU-UB7	CCACCTTCCTCCMGTATMTCA

Table S2. Taxon information for figure 1

Asterids			
Apiales	Apiaceae	Daucus_carota	AF301604
Apiales	Araliaceae	Panax_ginseng	AF034118
Aquifoliales	Aquifoliaceae	Ilex_repanda	AY725899
Aquifoliales	Aquifoliaceae	Ilex_verticillata	AY741812
Asterales	Asteraceae	Helianthus_annuus	X52838
Asterales	Campanulaceae	Campanula_garganica	AY741815
Asterales	Goodeniaceae	Goodenia_ovata	AY818934
Cornales	Cornaceae	Cornus_sericea	AY725897
Cornales	Cornaceae	Cornus_suecica	AF420915
Cornales	Hydrangeaceae	Hydrangea_sp._JS-2005	AY725896
Dipsacales	Adoxaceae	Sambucus_sieboldiana	AY741813
Dipsacales	Dipsacaceae	Dipsacus_fullonum	AY741814
Ericales	Actinidiaceae	Actinidia_arguta	AF420916
Ericales	Balsaminaceae	Impatiens_parviflora	AF420933
Ericales	Clethraceae	Clethra_arborea	AF420919
Ericales	Clethraceae	Clethra_barbinervis	AF420920
Ericales	Cyrillaceae	Cyrilla_racemiflora	AF420922
Ericales	Diapensiaceae	Diapensia_lapponica	AF420923
Ericales	Diapensiaceae	Galax_urceolata	AF420929
Ericales	Diapensiaceae	Schizocodon_soldanelloides	AF420949
Ericales	Diapensiaceae	Shortia_uniflora	AY725915
Ericales	Ebenaceae	Diospyros_digyna	AF420924
Ericales	Ebenaceae	Lissocarpa_guianensis	AF420934
Ericales	Ericaceae	Chimaphila_umbellata	AF420917
Ericales	Ericaceae	Empetrum_nigrum	AF420925
Ericales	Ericaceae	Enkianthus_campanulatus	AF420926
Ericales	Ericaceae	Rhododendron_impeditum	AY725911
Ericales	Ericaceae	Vaccinium_uliginosum	AF420953
Ericales	Fouquieriaceae	Fouquieria_columnaris	AY725902
Ericales	Fouquieriaceae	Fouquieria_fasciculata	AY725903
Ericales	Fouquieriaceae	Fouquieria_sp._Anderberg_s.n.	AF420928
Ericales	Lecythidaceae	Barringtonia_asiatica	AY725906
Ericales	Lecythidaceae	Couroupita_guianensis	AY725907
Ericales	Lecythidaceae	Napoleona_imperialis	AF420960
Ericales	Maesaceae	Maesa_tenera	AF420937
Ericales	Marcgraviaceae	Marcgravia_sp._Anderberg_s.n.	AF420939
Ericales	Marcgraviaceae	Norantea_peduncularis	AF420941
Ericales	Mitrastemonaceae	Mitrastema_yamamotoi	AY739068
Ericales	Myrsinaceae	Lysimachia_vulgaris	AF420935
Ericales	Myrsinaceae	Myrsine_africana	AF420940
Ericales	Pellicieraceae	Pelliciera rhizophorae	AY725900
Ericales	Pentaphylacaceae	Pentaphylax_euryoides	AF419242
Ericales	Polemoniaceae	Acanthogilia_gloriosa	AY725904
Ericales	Polemoniaceae	Cantua_buxifolia	AY725905
Ericales	Polemoniaceae	Cobaea_scandens	AF420921
Ericales	Polemoniaceae	Polemonium_caeruleum	AF420944
Ericales	Sapotaceae	Madhuca_microphylla	AF420936
Ericales	Sapotaceae	Manilkara_zapota	AF420938
Ericales	Sapotaceae	Palaquium_formosanum	AF420942
Ericales	Sapotaceae	Planchonella_obovata	AF420945
Ericales	Sapotaceae	Roridula_gorgonias	AY725910
Ericales	Sapotaceae	Sarcosperma_laurinum	AF420956
Ericales	Sarraceniaceae	Heliamphora_sp._Anderberg_s.n.	AF420932
Ericales	Sarraceniaceae	Sarracenia_flava	AF420947
Ericales	Sladeniaceae	Ficalhoa_laurifolia	AF420955
Ericales	Sladeniaceae	Sladenia_celastrifolia	AF420959

Ericales	Styracaceae	Bruinsmia_styracoides	AY725914
Ericales	Styracaceae	Halesia_carolina	AF420931
Ericales	Styracaceae	Styrax_officinalis	AF420950
Ericales	Symplocaceae	Symplocos_bogotensis	AF420951
Ericales	Symplocaceae	Symplocos_sp._Chung_and_Anderberg_1351	AF420954
Ericales	Symplocaceae	Symplocos_zizyphoides	AY725913
Ericales	Ternstroemiaceae	Cleyera_japonica	AY725908
Ericales	Ternstroemiaceae	Eurya_sp._Chung_&_Anderberg_1406	AF420927
Ericales	Ternstroemiaceae	Ternstroemia_stahlii	AY725909
Ericales	Tetrameristaceae	Pentamerista_neotropica	AY725901
Ericales	Tetrameristaceae	Tetramerista_sp._Coode_7925	AF420958
Ericales	Theaceae	Camellia_sinensis	AF420952
Ericales	Theaceae	Gordonia_axillaris	AF420930
Ericales	Theaceae	Schima_sp._JS-2005	AY725912
Ericales	Theaceae	Schima_superba	AF420948
Ericales	Theophrastaceae	Clavija_domingensis	AF420918
Ericales	Theophrastaceae	Samolus_repens	AF420946
Garryales	Aucubaceae	Aucuba_japonica	AY725898
Gentianales	Gelsemiaceae	Gelsemium_sempervirens	AY741816
Gentianales	Gentianaceae	Gentiana_procera	AY741817
Gentianales	Loganiaceae	Strychnos_spinosa	AY741818
Lamiales	Acanthaceae	Justicia_carnea	AY741824
Lamiales	Acanthaceae	Strobilanthes_dyeriana	AY741825
Lamiales	Bignoniaceae	Campsis_radicans	AY741839
Lamiales	Bignoniaceae	Catalpa_bignonioides	AY741840
Lamiales	Calceolariaceae	Jovellana_sp._JPM-2004	AY741822
Lamiales	Gesneriaceae	Streptocarpus_holstii	AY741823
Lamiales	Lamiaceae	Ajuga_reptans	AY818931
Lamiales	Lamiaceae	Mentha_spicata	AY741835
Lamiales	Lamiaceae	Stachys_officinalis	AY741836
Lamiales	Oleaceae	Syringa_vulgaris	AY741821
Lamiales	Orobanchaceae	Bartsia_inaequalis	AY741832
Lamiales	Orobanchaceae	Bartsia_laticrenata	AY741833
Lamiales	Orobanchaceae	Lamourouxia_viscosa	AY741830
Lamiales	Orobanchaceae	Lindenbergia_urticifolia	AY741829
Lamiales	Orobanchaceae	Orobanche_fasciculata	AY741831
Lamiales	Orobanchaceae	Parentucellia_viscosa	AY741834
Lamiales	Paulowniaceae	Paulownia_tomentosa	AY741826
Lamiales	Pedaliaceae	Sesamum_indicum	AY741827
Lamiales	Plantaginaceae	Digitalis_purpurea	AY741841
Lamiales	Plantaginaceae	Globularia_punctata	AY741842
Lamiales	Plantaginaceae	Plantago_atrata	AY818936
Lamiales	Plantaginaceae	Plantago_australis	AY741847
Lamiales	Plantaginaceae	Plantago_coronopus	AY741843
Lamiales	Plantaginaceae	Plantago_crassifolia	AY741844
Lamiales	Plantaginaceae	Plantago_lanceolata	AY818937
Lamiales	Plantaginaceae	Plantago_macrorhiza	AY741845
Lamiales	Plantaginaceae	Plantago_media	AY818938
Lamiales	Plantaginaceae	Plantago_rigida	AY741848
Lamiales	Plantaginaceae	Plantago_rugelii	AY818939
Lamiales	Plantaginaceae	Plantago_sempervirens	AY818940
Lamiales	Plantaginaceae	Plantago_sericea	AY818941
Lamiales	Plantaginaceae	Plantago_subspathulata	AY741846
Lamiales	Plantaginaceae	Plantago_tubulosa	AY741849
Lamiales	Plantaginaceae	Veronica_incana	AY818943
Lamiales	Scrophulariaceae	Alonsoa_sp._JPM-2004	AY741837
Lamiales	Scrophulariaceae	Myoporum_sandwicense	AY741838
Lamiales	Verbenaceae	Verbena_bonariensis	AY741828

Solanales	Convolvulaceae	Astripomoea_malvacea	AY596673
Solanales	Convolvulaceae	Bonamia_media	AY596689
Solanales	Convolvulaceae	Convolvulus_assyricus	AY596678
Solanales	Convolvulaceae	Cuscuta_campestris	AY940733
Solanales	Convolvulaceae	Cuscuta_europaea	AY596701
Solanales	Convolvulaceae	Cuscuta_japonica	AY596702
Solanales	Convolvulaceae	Cuscuta_sandwichiana	AY741820
Solanales	Convolvulaceae	Dicranostyles_ampla	AY596694
Solanales	Convolvulaceae	Dinetus_truncatus	AY596699
Solanales	Convolvulaceae	Erycibe_glomerata	AY596697
Solanales	Convolvulaceae	Erycibe_hellwigii	AY596696
Solanales	Convolvulaceae	Evolvulus_glomeratus	AY596684
Solanales	Convolvulaceae	Falkia_repens	AY596687
Solanales	Convolvulaceae	Hildebrandtia_valo	AY596682
Solanales	Convolvulaceae	Humbertia_madagascariensis	AY741819
Solanales	Convolvulaceae	Ipomoea_batatas	AY596672
Solanales	Convolvulaceae	Ipomoea_pes-tigridis	AY596675
Solanales	Convolvulaceae	Iseia_luxurians	AY596679
Solanales	Convolvulaceae	Jacquemontia_blanchetii	AY596693
Solanales	Convolvulaceae	Jacquemontia_tamnifolia	AY596692
Solanales	Convolvulaceae	Lepistemon_owariensis	AY596674
Solanales	Convolvulaceae	Maripa_repens	AY596695
Solanales	Convolvulaceae	Merremia_peltata	AY596677
Solanales	Convolvulaceae	Merremia_vitifolia	AY596676
Solanales	Convolvulaceae	Neuropeltis_acuminata	AY596690
Solanales	Convolvulaceae	Odonellia_hirtiflora	AY596680
Solanales	Convolvulaceae	Porana_commixta	AY596700
Solanales	Convolvulaceae	Porana_velutina	AY596688
Solanales	Convolvulaceae	Poranopsis_paniculata	AY596698
Solanales	Convolvulaceae	Rapona_tiliifolia	AY596691
Solanales	Convolvulaceae	Seddera_hirsuta	AY596683
Solanales	Convolvulaceae	Stylisma_patens	AY596685
Solanales	Convolvulaceae	Tetralocularia_pennellii	AY596681
Solanales	Convolvulaceae	Wilsonia_backhousei	AY596686
Solanales	Montiniaceae	Montinia_caryophyllacea	AY596706
Solanales	Solanaceae	Nicotiana_plumbaginifolia	X07745
Solanales	Solanaceae	Nicotiana_repanda	AF056245
Solanales	Solanaceae	Nicotiana_tabacum	BA000042
Solanales	Solanaceae	Petunia_axillaris_subsp._parodii	U61391
Solanales	Solanaceae	Schizanthus_pinnatus	AY596705
Caryophyllids			
Caryophyllales	Amaranthaceae	Beta_vulgaris_subsp._vulgaris	BA000009
Caryophyllales	Caryophyllaceae	Silene_acaulis	EF547203
Caryophyllales	Caryophyllaceae	Silene_latifolia	EF547204
Caryophyllales	Caryophyllaceae	Silene_noctiflora	EF547205
Caryophyllales	Caryophyllaceae	Silene_uniflora	DQ841782
Caryophyllales	Caryophyllaceae	Silene_vulgaris	DQ841789
Caryophyllales	Caryophyllaceae	Stellaria_sp._Qiu	EF547206
Caryophyllales	Nepenthaceae	Nepenthes_sp._Kosobe	EF547202
Caryophyllales	Nyctaginaceae	Bougainvillea_glabra	AY818932
Rosids			
Brassicales	Brassicaceae	Arabidopsis_thaliana	Y08501
Brassicales	Brassicaceae	Brassica_juncea	AY211266
Brassicales	Brassicaceae	Brassica_napus	AP006444
Brassicales	Brassicaceae	Brassica_rapa	AF076166
Cucurbitales	Apodanthaceae	Apodanthes_caseariae	AY739071
Cucurbitales	Apodanthaceae	Berlinianche_aethiopica	AY739072
Cucurbitales	Apodanthaceae	Pilostyles_thurberi	AY739073

Fabales	Fabaceae	Glycine_max	Z14031
Fabales	Fabaceae	Phaseolus_vulgaris	M64246
Fabales	Fabaceae	Pisum_sativum	X05366
Fabales	Fabaceae	Vigna_radiata	AF071550
Geraniales	Geraniaceae	Erodium_pelargoniflorum	DQ317061
Geraniales	Geraniaceae	Pelargonium_x_hortorum	DQ317063
Geraniales	Geraniaceae	Sarcocaulon_vanderietiae	DQ317062
Geraniales	Hypseocharitaceae	Hypseocharis_pimpinellifolia	DQ317060
Malpighiales	Rafflesiaceae	Rafflesia_pricei	AY739075
Malpighiales	Rafflesiaceae	Rafflesia_tuan-mudae	AY739074
Malpighiales	Rafflesiaceae	Rhizanthes_infanticida	AY739079
Malpighiales	Rafflesiaceae	Sapria_himalayana	AY739076
Malpighiales	Rafflesiaceae	Sapria_poilanei	AY739077
Malpighiales	Rafflesiaceae	Sapria_ram	AY739078
Malvales	Cytinaceae	Bdallophytum_americanum	AY739070
Malvales	Cytinaceae	Cytinus_ruber	AY739069
Malvales	Malvaceae	Abutilon_x_hybridum	AY739066
Malvales	Malvaceae	Hibiscus_rosa-sinensis	AY818935
Malvales	Malvaceae	Pavonia_spinifex	AY739067
Myrtales	Onagraceae	Oenothera_berteroana	M24235
Myrtales	Onagraceae	Oenothera_biennis	X04023
Rosales	Moraceae	Ficus_pumila	AY818933
Rosales	Rosaceae	Rubus_sp._JPM-2004	AY818942
Sapindales	Sapindaceae	Aesculus_californica	AY818930
Saxifragales	Cercidiphyllaceae	Cercidiphyllum_japonicum	AY299744
Stem Eudicots			
Buxales	Buxaceae	Buxus_sempervirens	AF197636
Buxales	Buxaceae	Pachysandra_procumbens	AF197634
Buxales	Buxaceae	Sarcococca_confusa	AF197635
Buxales	Didymelaceae	Didymeles_perrieri	AF197637
Proteales	Nelumbonaceae	Nelumbo_lutea	AY009420
Proteales	Nelumbonaceae	Nelumbo_nucifera	AF197654
Proteales	Platanaceae	Platanus_occidentalis	AY009423
Proteales	Proteaceae	Grevillea_robusta	AY009422
Proteales	Proteaceae	Persoonia_katerae	AF197652
Proteales	Proteaceae	Petrophile_canescens	AF197653
Ranunculales	Berberidaceae	Epimedium_grandiflorum	AY299765
Ranunculales	Berberidaceae	Mahonia_bealei	AF197659
Ranunculales	Berberidaceae	Podophyllum_peltatum	AF197660
Ranunculales	Eupteleaceae	Euptelea_polyandra	AF197650
Ranunculales	Fumariaceae	Dicentra_sp._Qiu_95026	AF197649
Ranunculales	Fumariaceae	Pseudofumaria lutea	AY009416
Ranunculales	Lardizabalaceae	Akebia_quinata	AF197642
Ranunculales	Lardizabalaceae	Lardizabala_biternata	AF197643
Ranunculales	Lardizabalaceae	Sargentodoxa_cuneata	AF197644
Ranunculales	Lardizabalaceae	Stauntonia_hexaphylla	AY299841
Ranunculales	Menispermaceae	Cissampelos_pareira	AF197645
Ranunculales	Menispermaceae	Cocculus_trilobus	AF197646
Ranunculales	Papaveraceae	Sanguinaria_canadensis	AF197651
Ranunculales	Ranunculaceae	Aquilegia_canadensis	AY394727
Ranunculales	Ranunculaceae	Ranunculus_sp._Qiu_95024	AF197714
Ranunculales	Ranunculaceae	Xanthorhiza_simplicissima	AF197658
Sabiales	Sabiaceae	Meliosma_squamulata	AF197656
Sabiales	Sabiaceae	Sabia_sp._Qiu_91025	AF197657
Trochodendrales	Trochodendraceae	Tetracentron_sinense	AF197647
Trochodendrales	Trochodendraceae	Trochodendron_aralioides	AF197648
Magnoliids			
Canellales	Canellaceae	Canella_winterana	AF197676

Canellales	Canellaceae	Cinnamodendron_ekmanii	AF197677
Canellales	Winteraceae	Drimys_winteri	AF197673
Canellales	Winteraceae	Pseudowintera_axillaris	AY394731
Canellales	Winteraceae	Takhtajania_perrieri	DQ007416
Canellales	Winteraceae	Tasmannia_insipida	AF197674
Canellales	Winteraceae	Tasmannia_lanceolata	AY299847
Canellales	Winteraceae	Zygogynum_pauciflorum	AF197675
Laurales	Atherospermataceae	Atherosperma_moschatum	AF197683
Laurales	Atherospermataceae	Daphnandra_micrantha	AF197684
Laurales	Atherospermataceae	Doryphora_sassafras	AF197688
Laurales	Calycanthaceae	Calycanthus_floridus	AF197678
Laurales	Calycanthaceae	Calycanthus_occidentalis	AY299739
Laurales	Calycanthaceae	Chimonanthus_praecox	AF197679
Laurales	Calycanthaceae	Idiospermum_australiense	AF197680
Laurales	Hernandiaceae	Gyrocarpus_americanus	AY299773
Laurales	Hernandiaceae	Gyrocarpus_sp._Chase_317	AF197701
Laurales	Hernandiaceae	Hernandia_ovigera	DQ007413
Laurales	Lauraceae	Cinnamomum_camphora	AF197681
Laurales	Lauraceae	Cinnamomum_verum	AY009415
Laurales	Lauraceae	Cryptocarya_meissneriana	AF197702
Laurales	Lauraceae	Laurus_nobilis	AF197682
Laurales	Lauraceae	Neolitsea_cassia	AY299811
Laurales	Monimiaceae	Hedycarya_arborea	AF197689
Laurales	Monimiaceae	Hortonia_floribunda	DQ007414
Laurales	Monimiaceae	Palmeria_scandens	AF197685
Laurales	Monimiaceae	Peumus_boldus	AF197686
Laurales	Siparunaceae	Siparuna_decipiens	AF197687
Magnoliales	Annonaceae	Annona_muricata	AF197695
Magnoliales	Annonaceae	Asimina_triloba	AF197696
Magnoliales	Annonaceae	Cananga_odorata	AF197700
Magnoliales	Annonaceae	Polyalthia_suberosa	AF197694
Magnoliales	Degeneriaceae	Degeneria_vitiensis	AF293752
Magnoliales	Eupomatiaceae	Eupomatia_bennettii	AF197692
Magnoliales	Eupomatiaceae	Eupomatia_laurina	AY299767
Magnoliales	Himantandraceae	Galbulimima_belgraveana	AF197693
Magnoliales	Magnoliaceae	Liriodendron_chinense	AF197690
Magnoliales	Magnoliaceae	Liriodendron_tulipifera	AY394730
Magnoliales	Magnoliaceae	Magnolia_grandiflora	AF209100
Magnoliales	Magnoliaceae	Magnolia_tripetala	AF197691
Magnoliales	Magnoliaceae	Michelia_figo	AY299802
Magnoliales	Myristicaceae	Knema_kinabaluensis	AY009419
Magnoliales	Myristicaceae	Knema_latericia	AF197697
Magnoliales	Myristicaceae	Mauloutchia_chapelieri	AF197699
Magnoliales	Myristicaceae	Myristica_fragrans	AF197698
Piperales	Aristolochiaceae	Aristolochia_elegans	AY009408
Piperales	Aristolochiaceae	Aristolochia_gigantea	AY299718
Piperales	Aristolochiaceae	Aristolochia_macrophylla	AF197669
Piperales	Aristolochiaceae	Asarum_canadense	AF197671
Piperales	Aristolochiaceae	Saruma_henryi	AF197672
Piperales	Aristolochiaceae	Thottea_tomentosa	AF197670
Piperales	Hydnoraceae	Hydnora_africana	AF503356
Piperales	Hydnoraceae	Prosopanche_americana	AF503357
Piperales	Lactoridaceae	Lactoris_fernandeziana	AF197710
Piperales	Piperaceae	Macropiper_excelsum	AY299799
Piperales	Piperaceae	Peperomia_obtusifolia	AF197629
Piperales	Piperaceae	Peperomia_polybotrya	AY299819
Piperales	Piperaceae	Piper_betle	AF197630
Piperales	Piperaceae	Piper_bicolor	AY009421

	Piperales	Piperaceae	Piper_nigrum	AF039243
	Piperales	Saururaceae	Anemopsis_californica	AF197631
	Piperales	Saururaceae	Houttuynia_cordata	AF197632
	Piperales	Saururaceae	Saururus_cernuus	AF197633
	Piperales	Saururaceae	Saururus_chinensis	AY009424
Chloranthales				
	Chloranthales	Chloranthaceae	Ascarina_sp._Qiu-M149	AF197667
	Chloranthales	Chloranthaceae	Chloranthus_multistachys	AF197665
	Chloranthales	Chloranthaceae	Chloranthus_spicatus	AY299746
	Chloranthales	Chloranthaceae	Hedyosmum_arborescens	AF197668
	Chloranthales	Chloranthaceae	Hedyosmum_sp._CCWD-2000	AY009414
	Chloranthales	Chloranthaceae	Hedyosmum_sp._Stevenson_1188	AY299777
	Chloranthales	Chloranthaceae	Sarcandra_chloranthoides	AF197666
Monocots				
	Acorales	Acoraceae	Acorus_calamus	AF039256
	Acorales	Acoraceae	Acorus_gramineus	AF197622
	Acorales	Acoraceae	Acorus_tatarinowii	AY299700
	Alismatales	Alismataceae	Alisma_plantago-aquatica	AF197717
	Alismatales	Alismataceae	Caldesia_oligococca	AY277800
	Alismatales	Alismataceae	Sagittaria_latifolia	AY394732
	Alismatales	Araceae	Arisaema_triphyllum	AY299717
	Alismatales	Araceae	Gymnostachys_anceps	AF039244
	Alismatales	Araceae	Orontium_aquaticum	AF197705
	Alismatales	Araceae	Spathiphyllum_wallisii	AF197706
	Alismatales	Araceae	Symplocarpus_foetidus	AF039245
	Alismatales	Butomaceae	Butomus_umbellatus	AY299733
	Alismatales	Cymodoceaceae	Cymodocea_serrulata	AY277801
	Alismatales	Hydrocharitaceae	Ottelia_ovalifolia	AY277802
	Alismatales	Juncaginaceae	Triglochin_maritima	AF197716
	Alismatales	Potamogetonaceae	Potamogeton_berchtoldii	AF197715
	Alismatales	Potamogetonaceae	Potamogeton_natans	AY299829
	Alismatales	Scheuchzeriaceae	Scheuchzeria_palustris	AY277803
	Alismatales	Tofieldiaceae	Pleea_tenuifolia	AF197703
	Alismatales	Tofieldiaceae	Tofieldia_calyculata	AF197704
	Arecales	Arecaceae	Calamus_caryotoides	AY299734
	Arecales	Arecaceae	Calamus_usitatus	U58832
	Arecales	Arecaceae	Euterpe_oleracea	AY299769
	Arecales	Arecaceae	Nypa_fruticans	U58833
	Arecales	Arecaceae	Phoenix_reclinata	U58831
	Arecales	Arecaceae	Phytelephas_aequatorialis	AY299825
	Arecales	Arecaceae	Plectocomia_elongata	AY299826
	Arecales	Arecaceae	Trithrinax_acanthocoma	AY299853
	Asparagales	Agapanthaceae	Agapanthus_africanus	AY299701
	Asparagales	Agavaceae	Agave_bracteosa	AY299702
	Asparagales	Agavaceae	Agave_ghiesbreghtii	AY299703
	Asparagales	Agavaceae	Anemarrhena_asphodeloides	AY299711
	Asparagales	Agavaceae	Anthericum_sp._Weigend_et_Weigend_2000/154	AY299713
	Asparagales	Agavaceae	Behnia_reticulata	AY299726
	Asparagales	Agavaceae	Herreria_montevidensis	AY299781
	Asparagales	Agavaceae	Sowerbaea_laxiflora	AY299838
	Asparagales	Alliaceae	Allium_altaicum	AY299707
	Asparagales	Alliaceae	Ipheion_uniflorum	AY299787
	Asparagales	Amaryllidaceae	Clivia_nobilis	AY299749
	Asparagales	Aphyllanthaceae	Aphyllanthes_monspeliensis	AY299714
	Asparagales	Asparagaceae	Asparagus_officinalis	AF197713
	Asparagales	Asphodelaceae	Asphodelus_aestivus	AY299721
	Asparagales	Asphodelaceae	Kniphofia_uvaria	AY299792
	Asparagales	Asteliaceae	Astelia_sp._Grimes_3525	AY299722

Asparagales	Asteliaceae	Neoastelia_spectabilis	AY299810
Asparagales	Blandfordiaceae	Blandfordia_grandiflora	AY299727
Asparagales	Boryaceae	Alania_endlicheri	AY299705
Asparagales	Boryaceae	Borya_aff._sphaerocephala_Conran_et_al._944	AY299728
Asparagales	Doryanthaceae	Doryanthes_excelsa	AY299760
Asparagales	Hemerocallidaceae	Dianella_caerulea	AY299756
Asparagales	Hemerocallidaceae	Johnsonia_lupulina	AY299791
Asparagales	Hyacinthaceae	Muscari_neglectum	AY299807
Asparagales	Hyacinthaceae	Scilla_libanotica	AY299836
Asparagales	Hypoxidaceae	Curculigo_capitulata	AF039249
Asparagales	Hypoxidaceae	Hypoxis_occidentalis	AY299784
Asparagales	Iridaceae	Neomarica_northiana	AY299812
Asparagales	Iridaceae	Sisyrinchium_angustifolium	AY299837
Asparagales	Ixiolirionaceae	Ixiolirion_tataricum	AY299789
Asparagales	Lanariaceae	Lanaria_lanata	AY299796
Asparagales	Laxmanniaceae	Arthropodium_cirrhatum	AY299719
Asparagales	Laxmanniaceae	Thysanotus_thyrsoideus	AY299850
Asparagales	Orchidaceae	Calopogon_tuberosus	AY299738
Asparagales	Orchidaceae	Cypripedium_calceolus	AY299755
Asparagales	Orchidaceae	Epipactis_helleborine	AY299766
Asparagales	Orchidaceae	Isotria_verticillata	AY299788
Asparagales	Orchidaceae	Neuwiedia_veratrifolia	AY299813
Asparagales	Ruscaceae	Convallaria_keiskei	AY299752
Asparagales	Tecophilaeaceae	Tecophilaea_cyanocrocus	AY299848
Asparagales	Themidaceae	Brodiaea_californica	AY299730
Asparagales	Xanthorrhoeaceae	Xanthorrhoea_australis	AF039250
Asparagales	Xeronemataceae	Xeronema_callistemon	AY299857
Commelinales	Commelinaceae	Callisia_warszewicziana	AY299736
Commelinales	Commelinaceae	Cochliostema_odoratissimum	AY299750
Commelinales	Commelinaceae	Commelina_communis	AY299751
Commelinales	Commelinaceae	Dichorisandra_thyrsiflora	AY299757
Commelinales	Commelinaceae	Murdannia_sp._BH_75-650	AY299805
Commelinales	Commelinaceae	Palisota_bracteosa	AY299817
Commelinales	Haemodoraceae	Anigozanthos_flavidus	AF039246
Commelinales	Haemodoraceae	Haemodorum_simulans	AY299774
Commelinales	Haemodoraceae	Xiphidium_caeruleum	AY299858
Commelinales	Hanguanaceae	Hanguana_malayana	AY299775
Commelinales	Philydraceae	Helmholtzia_glaberrima	AY299779
Commelinales	Philydraceae	Philydrella_pygmaea	AY299823
Commelinales	Philydraceae	Philydrum_lanuginosum	AY299824
Commelinales	Pontederiaceae	Eichhornia_azurea	AY299762
Commelinales	Pontederiaceae	Eichhornia_paniculata	AY299763
Commelinales	Pontederiaceae	Heteranthera_rotundifolia	AY299782
Commelinales	Pontederiaceae	Hydrothrix_gardneri	AY299783
Commelinales	Pontederiaceae	Monochoria_korsakowii	AY299803
Commelinales	Pontederiaceae	Pontederia_cordata	AY299828
Dasypogonales	Dasypogonaceae	Baxteria_australis	AY124504
Dasypogonales	Dasypogonaceae	Calectasia_cyanea	AY124505
Dasypogonales	Dasypogonaceae	Dasypogon_hookeri	AY124503
Dasypogonales	Dasypogonaceae	Kingia_australis	AY124506
Dioscoreales	Burmanniaceae	Burmannia_lutescens	AY299732
Dioscoreales	Burmanniaceae	Thismia_rodwayi	AY299849
Dioscoreales	Dioscoreaceae	Dioscorea_communis	AY277804
Dioscoreales	Dioscoreaceae	Dioscorea_mexicana	AY009417
Dioscoreales	Dioscoreaceae	Dioscorea_retusa	AY299759
Dioscoreales	Dioscoreaceae	Dioscorea_sp._Qiu_94044	AF197709
Dioscoreales	Dioscoreaceae	Tacca_leontopetaloides	AF039252
Dioscoreales	Dioscoreaceae	Tacca_parkeri	AY299845

Dioscoreales	Dioscoreaceae	Trichopus_sempervirens	AY299724
Dioscoreales	Dioscoreaceae	Trichopus_zeylanicus	AY277805
Dioscoreales	Nartheciaceae	Aletris_farinosa	AY299706
Dioscoreales	Nartheciaceae	Narthecium_ossifragum	AY299809
Liliales	Alstroemeriaceae	Alstroemeria_caryophyllaea	AF039254
Liliales	Campynemataceae	Campynema_lineare	AY299740
Liliales	Colchicaceae	Burchardia_multiflora	AY299731
Liliales	Colchicaceae	Clintonia_borealis	AY299748
Liliales	Colchicaceae	Schelhammera_multiflora	AY299834
Liliales	Colchicaceae	Wurmbea_sp._Conran_et_al._899	AY299856
Liliales	Corsiaceae	Arachnitis_uniflora	AY299715
Liliales	Liliaceae	Calochortus_minimus	AY299737
Liliales	Liliaceae	Lilium_superbum	AY299797
Liliales	Liliaceae	Lilium_tigrinum	AY394729
Liliales	Luzuriagaceae	Luzuriaga_radicans	AY299798
Liliales	Melanthiaceae	Amianthium_muscitoxicum	AY299709
Liliales	Melanthiaceae	Chamaelirium_luteum	AY299745
Liliales	Melanthiaceae	Trillium_grandiflorum	AF039253
Liliales	Melanthiaceae	Veratrum_viride	AF039255
Liliales	Petermanniaceae	Petermannia_cirrosa	AY299820
Liliales	Philesiaceae	Eustrephus_latifolius	AY299768
Liliales	Philesiaceae	Philesia_magellanica	AY299822
Liliales	Ripogonaceae	Ripogonum_discolor	AY299831
Liliales	Smilacaceae	Geitonoplesium_cymosum	AY299771
Liliales	Smilacaceae	Smilax_rotundifolia	AF039251
Pandanales	Cyclanthaceae	Carludovica_palmata	AF197707
Pandanales	Cyclanthaceae	Chorigyne_cylindrica	AY299747
Pandanales	Cyclanthaceae	Cyclanthus_bipartitus	AY299754
Pandanales	Cyclanthaceae	Sphaeradenia_stenosperma	AY299840
Pandanales	Pandanaceae	Freycinetia_multiflora	AY299770
Pandanales	Pandanaceae	Pandanus_copelandii	AY299818
Pandanales	Stemonaceae	Croomia_pauciflora	AF197708
Pandanales	Stemonaceae	Stemona_javanica	AY299842
Pandanales	Triuridaceae	Lacandonia_schismatica	AY299794
Pandanales	Triuridaceae	Sciaphila_albescens	AY299835
Pandanales	Triuridaceae	Triuris_sp._Vergara_Silva_s.n.	AY299854
Pandanales	Velloziaceae	Acanthochlamys_bracteata	AY299698
Pandanales	Velloziaceae	Barbaceniopsis_sp._Weigend_et_Weigend_2000-318	AY299725
Pandanales	Velloziaceae	Vellozia_elegans	AF039247
Petrosaviales	Petrosaviaceae	Japonolirion_osense	AY299790
Petrosaviales	Petrosaviaceae	Petrosavia_stellaris	AY299821
Poales	Anarthriaceae	Anarthria_prolifera	AY124513
Poales	Bromeliaceae	Ananas_comosus	AY299710
Poales	Bromeliaceae	Brocchinia_reducta	AY299729
Poales	Bromeliaceae	Catopsis_nutans	AF039257
Poales	Bromeliaceae	Hechtia_texensis	AY299776
Poales	Bromeliaceae	Puya_berteroniana	AY124508
Poales	Bromeliaceae	Tillandsia_usneoides	AY124507
Poales	Cyperaceae	aff._Cladium_sp._JID-2002	AY124515
Poales	Cyperaceae	Carex_interior	AY124514
Poales	Ecdeiocoleaceae	Ecdeiocolea_monostachya	AY124516
Poales	Eriocaulaceae	Eriocaulon_aquaticum	AF039258
Poales	Eriocaulaceae	Eriocaulon_humboldtii	AY124517
Poales	Eriocaulaceae	Lachnocaulon_anceps	AY299795
Poales	Eriocaulaceae	Syngonanthus_flavidulus	AY299844
Poales	Eriocaulaceae	Tonina_fluviatilis	AY124518
Poales	Flagellariaceae	Flagellaria_indica	AF039248
Poales	Joinvilleaceae	Joinvillea_ascendens	AY124519

Poales	Joinvilleaceae	Joinvillea_plicata	AY394728
Poales	Juncaceae	Juncus_sp._JID-2002	AY124520
Poales	Juncaceae	Luzula_acuminata	AY124521
Poales	Mayacaceae	Mayaca_sellowiana	AY124522
Poales	Poaceae	Anomochloa_marantoidea	AY124526
Poales	Poaceae	Bambusa_multiplex	AY124525
Poales	Poaceae	Elymus_sibiricus	AJ291297
Poales	Poaceae	Oryza_sativa	BA000029
Poales	Poaceae	Pennisetum_glaucum	AF511560
Poales	Poaceae	Pharus_latifolius	AY124524
Poales	Poaceae	Secale_cereale	X99020
Poales	Poaceae	Secale_strictum_subsp._africanum	DQ401027
Poales	Poaceae	Streptochaeta_angustifolia	AY124523
Poales	Poaceae	Triticum_aestivum	AP008982
Poales	Poaceae	Triticum_turgidum_subsp._durum_x_Triticosecale	X80469
Poales	Poaceae	Zea_mays	AY506529
Poales	Rapateaceae	Cephalostemon_flavus	AY299742
Poales	Rapateaceae	Epidryos_allenii	AY299764
Poales	Rapateaceae	Kunhardtia_radiata	AY299793
Poales	Rapateaceae	Rapatea_xiphoides	AY124511
Poales	Rapateaceae	Schoenocephalium_cucullatum	AY124512
Poales	Rapateaceae	Spathanthus_bicolor	AY299839
Poales	Rapateaceae	Stegolepis_parvipetala	AY124535
Poales	Restionaceae	Baloskion_tetraphyllum	AY124529
Poales	Restionaceae	Elegia_fenestrata	AY124530
Poales	Restionaceae	Lepyrodia_scariosa	AY124528
Poales	Restionaceae	Thamnochortus_cinereus	AY124531
Poales	Sparganiaceae	Sparganium_eurycarpum	AY124509
Poales	Thurniaceae	Prionium_serratum	AY124527
Poales	Thurniaceae	Thurnia_polycephala	AY124532
Poales	Typhaceae	Typha_latifolia	AY124510
Poales	Xyridaceae	Abolboda_macrostachya	AY124533
Poales	Xyridaceae	Aratitiyopea_lopezii	AY299716
Poales	Xyridaceae	Orectanthe_sceptrum	AY124534
Poales	Xyridaceae	Xyris_bicephala	AY124536
Poales	Xyridaceae	Xyris_jupicai	AY299859
Zingiberales	Cannaceae	Canna_indica	AF039259
Zingiberales	Costaceae	Costus_lateriflorus	AY299753
Zingiberales	Costaceae	Dimerocostus_argenteus	AY299758
Zingiberales	Costaceae	Monocostus_uniflorus	AY299804
Zingiberales	Costaceae	Tapeinochilos_sp._L-86.0039	AY299846
Zingiberales	Heliconiaceae	Heliconia_rostrata	AY299778
Zingiberales	Lowiaceae	Orchidantha_maxillarioides	AY299815
Zingiberales	Marantaceae	Calathea_loeseneri	AY299735
Zingiberales	Marantaceae	Maranta_leuconeura	AY299801
Zingiberales	Musaceae	Musa_textilis	AY299806
Zingiberales	Strelitziaceae	Ravenala_madagascariensis	AY299830
Zingiberales	Strelitziaceae	Strelitzia_nicolai	AY299843
Zingiberales	Zingiberaceae	Alpinia_purpurata	AY299708
Zingiberales	Zingiberaceae	Globba_winitii	AY299772
Ceratophyllales			
Ceratophyllales	Ceratophyllaceae	Ceratophyllum_demersum	AF197627
Ceratophyllales	Ceratophyllaceae	Ceratophyllum_submersum	AF197628
Stem Angiosperms			
Amborellales	Amborellaceae	Amborella_trichopoda	AY009407
Austrobaileyales	Austrobaileyaceae	Austrobaileya_scandens	AF197664
Austrobaileyales	Schisandraceae	Illicium_anisatum	AY299786
Austrobaileyales	Schisandraceae	Illicium_floridanum	AF197663

Austrobaileyales	Schisandraceae	Illicium_lanceolatum	AF209101
Austrobaileyales	Schisandraceae	Kadsura_japonica	AF197661
Austrobaileyales	Schisandraceae	Schisandra_henryi	AY009425
Austrobaileyales	Schisandraceae	Schisandra_sphenanthera	AF197662
Austrobaileyales	Trimeniaceae	Trimenia_moorei	DQ007415
Austrobaileyales	Trimeniaceae	Trimenia_sp._CCWD-2000	AY009428
Nymphaeales	Cabombaceae	Brasenia_schreberi	AF197640
Nymphaeales	Cabombaceae	Cabomba_sp._Qiu_97027	AF197641
Nymphaeales	Nymphaeaceae	Nuphar_sp._Qiu_M114	AF197638
Nymphaeales	Nymphaeaceae	Nymphaea_odorata	AY299814
Nymphaeales	Nymphaeaceae	Nymphaea_sp._Qiu_91029	AF197639
Nymphaeales	Nymphaeaceae	Victoria_cruziana	AY299855
Gymnosperms			
Coniferales	Araucariaceae	Agathis_australis	AF209103
Coniferales	Araucariaceae	Araucaria_heterophylla	AF209104
Coniferales	Cephalotaxaceae	Cephalotaxus_harringtonia	DQ646222
Coniferales	Cupressaceae	Juniperus_virginiana	AF209106
Coniferales	Cupressaceae	Metasequoia_glyptostroboides	AF197619
Coniferales	Pinaceae	Abies_homolepis	DQ646224
Coniferales	Pinaceae	Cedrus_deodara	DQ646223
Coniferales	Pinaceae	Pinus_sp._Qiu_94013	AF197626
Coniferales	Pinaceae	Pinus_strobus	AF209108
Coniferales	Podocarpaceae	Phyllocladus_aspleniifolius	DQ646219
Coniferales	Podocarpaceae	Podocarpus_macrophyllus	AF209105
Coniferales	Sciadopityaceae	Sciadopitys_verticillata	DQ646220
Coniferales	Taxaceae	Taxus_x_media	DQ646221
Cycadales	Cycadaceae	Cycas_revoluta	AF197623
Cycadales	Stangeriaceae	Stangeria_eriopus	DQ646218
Cycadales	Zamiaceae	Zamia_furfuracea	AF209111
Cycadales	Zamiaceae	Zamia_integrifolia	AF197624
Ephedrales	Ephedraceae	Ephedra_distachya	DQ646225
Ginkgoales	Ginkgoaceae	Ginkgo_biloba	AF209110
Gnetales	Gnetaceae	Gnetum_gnemon	AF197617
Gnetales	Gnetaceae	Gnetum_ula	AF209109
Welwitschiales	Welwitschiaceae	Welwitschia_mirabilis	AF197618

Table S3. Taxon information for figure 2

Asterids			
Apiales	Apiaceae	Daucus_carota	AY820131
Apiales	Araliaceae	Hydrocotyle_rotundifolia	AJ223424
Aquifoliales	Aquifoliaceae	Ilex_sp._Qiu_94038	AJ223429
Asterales	Goodeniaceae	Goodenia_ovata	AY053575
Dipsacales	Adoxaceae	Sambucus_canadensis	AF193965
Ericales	Ebenaceae	Diospyros_virginiana	AJ223417
Ericales	Ericaceae	Pyrola_secunda	AJ247582
Ericales	Lecythidaceae	Barringtonia_asiatica	AJ247581
Ericales	Polemoniaceae	Phlox_paniculata	AY053576
Ericales	Sarraceniaceae	Heliamphora_heterodoxa	AY600113
Ericales	Symplocaceae	Symplocos_paniculata	AJ223435
Gentianales	Apocynaceae	Catharanthus_roseus	AJ223423
Gentianales	Apocynaceae	Hoya_lanceolata	AJ247588
Gentianales	Apocynaceae	Nerium_oleander	AJ223431
Gentianales	Loganiaceae	Strychnos_spinosa	AJ247596
Gentianales	Rubiaceae	Coffea_arabica	AJ247586
Gentianales	Rubiaceae	Ixora_sp._Qiu95051	AJ247587
Lamiales	Acanthaceae	Barleria_prionitis	AJ247601
Lamiales	Acanthaceae	Justicia_americana	AJ247602
Lamiales	Acanthaceae	Sanchezia_nobilis	AJ223437
Lamiales	Acanthaceae	Thunbergia_erecta	AJ247603
Lamiales	Bignoniaceae	Catalpa_fargesii	AJ223411
Lamiales	Byblidaceae	Byblis_liniflora	AY600112
Lamiales	Calceolariaceae	Calceolaria_sp._IUGH	AJ247585
Lamiales	Gesneriaceae	Columnea_sp._Lindqvist_and_Albert_30	AF482513
Lamiales	Gesneriaceae	Drymonia_serrulata	AJ247579
Lamiales	Gesneriaceae	Nematanthus_hirsutus	AJ247578
Lamiales	Lamiaceae	Ajuga_reptans	AJ247595
Lamiales	Lamiaceae	Clerodendrum_trichotomum	AJ223414
Lamiales	Lamiaceae	Lamium_sp._Qiu_95019	AJ223428
Lamiales	Lamiaceae	Physostegia_virginiana	AJ247594
Lamiales	Lamiaceae	Scutellaria_mociniana	AJ247593
Lamiales	Lentibulariaceae	Genlisea_aurea	AY600088
Lamiales	Lentibulariaceae	Genlisea_hispidula	AY600089
Lamiales	Lentibulariaceae	Genlisea_violacea	AY600090
Lamiales	Lentibulariaceae	Pinguicula_ehlersiae	AF482514
Lamiales	Lentibulariaceae	Pinguicula_gracilis	AF482515
Lamiales	Lentibulariaceae	Pinguicula_grandiflora	AF482516
Lamiales	Lentibulariaceae	Pinguicula_gypsicola	AF482517
Lamiales	Lentibulariaceae	Pinguicula_sp._Jobson_240	AY600087
Lamiales	Lentibulariaceae	Utricularia_adpressa	AF482518
Lamiales	Lentibulariaceae	Utricularia_alpina	AF482519
Lamiales	Lentibulariaceae	Utricularia_asplundii	AY600091
Lamiales	Lentibulariaceae	Utricularia_biloba	AY600092
Lamiales	Lentibulariaceae	Utricularia_caerulea	AY600093
Lamiales	Lentibulariaceae	Utricularia_costata	AY600094
Lamiales	Lentibulariaceae	Utricularia_dichotoma	AY600095
Lamiales	Lentibulariaceae	Utricularia_flaccida	AY600096
Lamiales	Lentibulariaceae	Utricularia_geminiscapa	AF482520
Lamiales	Lentibulariaceae	Utricularia_gibba	AY600097
Lamiales	Lentibulariaceae	Utricularia_longifolia	AY600098
Lamiales	Lentibulariaceae	Utricularia_meyeri	AY600099
Lamiales	Lentibulariaceae	Utricularia_multifida	AY600100
Lamiales	Lentibulariaceae	Utricularia_myriocista	AY600101
Lamiales	Lentibulariaceae	Utricularia_neottioides	AY600102
Lamiales	Lentibulariaceae	Utricularia_olivacea	AY600103

Lamiales	Lentibulariaceae	Utricularia_pubescens	AY128568
Lamiales	Lentibulariaceae	Utricularia_striatula	AY600104
Lamiales	Lentibulariaceae	Utricularia_subulata	AY600105
Lamiales	Lentibulariaceae	Utricularia_triloba	AF482521
Lamiales	Lentibulariaceae	Utricularia_violacea	AY600106
Lamiales	Oleaceae	Jasminum_polyanthum	AJ247607
Lamiales	Oleaceae	Olea_europaea	AF288707
Lamiales	Paulowniaceae	Paulownia_tomentosa	AJ247592
Lamiales	Pedaliaceae	Sesamum_indicum	AJ247598
Lamiales	Plantaginaceae	Callitriche_heterophylla	AJ247577
Lamiales	Plantaginaceae	Digitalis_purpurea	AJ223415
Lamiales	Plantaginaceae	Hebe_subalpina	AJ223419
Lamiales	Plantaginaceae	Plantago_atrata	AJ389610
Lamiales	Plantaginaceae	Plantago_australis	AJ389608
Lamiales	Plantaginaceae	Plantago_coronopus	AJ389609
Lamiales	Plantaginaceae	Plantago_cynops	AJ389612
Lamiales	Plantaginaceae	Plantago_lanceolata	AJ389611
Lamiales	Plantaginaceae	Plantago_media	AJ389605
Lamiales	Plantaginaceae	Plantago_rigida	AJ389607
Lamiales	Plantaginaceae	Plantago_rugelii	AJ389606
Lamiales	Plantaginaceae	Plantago_sericea	AJ389613
Lamiales	Plantaginaceae	Veronica_catenata	AJ223427
Lamiales	Scrophulariaceae	Celsia_arturus	AJ247590
Lamiales	Scrophulariaceae	Scrophularia_nodosa	AJ247591
Lamiales	Unplaced	Rehmannia_glutinosa	AJ247589
Solanales	Solanaceae	Lycopersicon_esculentum	X54738
Solanales	Solanaceae	Nicotiana_tabacum	BA000042
Solanales	Solanaceae	Solanum_commersonii	AJ582177
Solanales	Solanaceae	Solanum_tuberosum	X83206
Unplaced	Boraginaceae	Ehretia_anacua	AJ247606
Unplaced	Boraginaceae	Heliotropium_arborescens	AJ223425
Caryophyllids			
Caryophyllales	Amaranthaceae	Beta_vulgaris_subsp._vulgaris	X57693
Caryophyllales	Amaranthaceae	Spinacia_sp._CLP-2006	DQ317033
Caryophyllales	Caryophyllaceae	Gypsophila_repens	EF547217
Caryophyllales	Caryophyllaceae	Silene_acaulis	EF547218
Caryophyllales	Caryophyllaceae	Silene_latifolia	EF547221
Caryophyllales	Caryophyllaceae	Silene_noctiflora	EF547222
Caryophyllales	Caryophyllaceae	Stellaria_sp._Qiu	EF547228
Caryophyllales	Droseraceae	Aldrovanda_vesiculosa	AY600107
Caryophyllales	Droseraceae	Dionaea_muscipula	AY600108
Caryophyllales	Droseraceae	Drosera_capillaris	AY600109
Caryophyllales	Nepenthaceae	Nepenthes_sp._Jobson_1049	AY600110
Caryophyllales	Plumbaginaceae	Plumbago_sp._CLP-2006	DQ317032
Rosids			
Brassicales	Brassicaceae	Arabidopsis_thaliana	X94583
Brassicales	Brassicaceae	Brassica_juncea	AY300014
Brassicales	Brassicaceae	Brassica_napus	AP006444
Brassicales	Brassicaceae	Raphanus_sativus	X57692
Celastrales	Celastraceae	Brexia_madagascariensis	AJ223413
Crossosomatales	Crossosomataceae	Crossosoma_bigelovii	DQ317034
Cucurbitales	Cucurbitaceae	Cucumis_sativus	AJ223416
Fabales	Fabaceae	Glycine_max	M16884
Fabales	Fabaceae	Pisum_sativum	X14409
Fabales	Fabaceae	Vigna_radiata	AF338446
Fagales	Betulaceae	Betula_papyrifera	U77620
Geraniales	Geraniaceae	Erodium_chrysanthum	DQ317038
Geraniales	Geraniaceae	Erodium_pelargoniflorum	DQ317039

Geraniales	Geraniaceae	Erodium_trifolium	DQ317040
Geraniales	Geraniaceae	Geranium_himalayense	AF193968
Geraniales	Geraniaceae	Geranium_macrorrhizum	DQ317035
Geraniales	Geraniaceae	Monsonia_emarginata	DQ317036
Geraniales	Geraniaceae	Pelargonium_alchemilloides	DQ317046
Geraniales	Geraniaceae	Pelargonium_candicans	DQ317043
Geraniales	Geraniaceae	Pelargonium_capitatum	DQ317044
Geraniales	Geraniaceae	Pelargonium_cotyledonis	DQ317041
Geraniales	Geraniaceae	Pelargonium_quercetorum	DQ317045
Geraniales	Geraniaceae	Pelargonium_reniforme	DQ317042
Geraniales	Geraniaceae	Pelargonium_x_hortorum	DQ317047
Geraniales	Geraniaceae	Sarcocaulon_vanderietiae	DQ317037
Geraniales	Hypseocharitaceae	Hypseocharis_pimpinellifolia	AF193967
Malpighiales	Euphorbiaceae	Acalypha_sp._Qiu95079	AJ247597
Malpighiales	Euphorbiaceae	Croton_sp._Qiu_94027	AJ247608
Malpighiales	Euphorbiaceae	Euphorbia_milii	AJ223418
Malpighiales	Euphorbiaceae	Hevea_brasiliensis	AJ223436
Malpighiales	Euphorbiaceae	Hura_crepitans	AJ247584
Malpighiales	Linaceae	Linum_sp._Qiu96175	AJ247604
Malpighiales	Malpighiaceae	Malpighia_glabra	AJ223433
Malpighiales	Phyllanthaceae	Breynia_nivosa	AJ247605
Malpighiales	Salicaceae	Populus_tremuloides	U77623
Malpighiales	Violaceae	Viola_sp._Qiu95018	AJ247600
Malvales	Dipterocarpaceae	Shorea_talura	AJ247599
Myrtales	Onagraceae	Oenothera_berteriana	X05465
Myrtales	Onagraceae	Oenothera_biennis	AF020571
Oxalidales	Cephalotaceae	Cephalotus_follicularis	AY600111
Rosales	Rhamnaceae	Hovenia_dulcis	AJ247583
Rosales	Rhamnaceae	Rhamnus_cathartica	AJ223422
Rosales	Urticaceae	Pilea_fontana	AJ247580
Sapindales	Burseraceae	Bursera_sp._Qiu_94206	AJ223412
Sapindales	Meliaceae	Melia_toosendan	AJ223420
Stem Eudicots			
Buxales	Buxaceae	Buxus_sp._Qiu_94069	AF193962
Proteales	Nelumbonaceae	Nelumbo_lutea	AY009447
Proteales	Nelumbonaceae	Nelumbo_nucifera	AF193950
Proteales	Platanaceae	Platanus_occidentalis	AY009450
Proteales	Proteaceae	Grevillea_robusta	AY009449
Ranunculales	Eupteleaceae	Euptelea_polyandra	AF193963
Ranunculales	Fumariaceae	Pseudofumaria_lutea	AY009441
Ranunculales	Lardizabalaceae	Akebia_quinata	AY009429
Ranunculales	Ranunculaceae	Clematis_sp._Qiu_95085	AF193960
Ranunculales	Ranunculaceae	Ranunculus_carolinianus	AY009451
Ranunculales	Ranunculaceae	Ranunculus_sp._Qiu_95024	DQ317030
Santalales	Opiliaceae	Lepionurus_sylvestris	AJ223439
Trochodendrales	Tetracentraceae	Tetracentron_sinense	AY009455
Trochodendrales	Trochodendraceae	Trochodendron_aralioides	AF020581
Magnoliids			
Canellales	Canellaceae	Canella_winterana	AY009437
Canellales	Winteraceae	Drimys_winteri	AY009443
Laurales	Calycanthaceae	Calycanthus_floridus	AY009436
Laurales	Lauraceae	Cinnamomum_verum	AY009440
Laurales	Lauraceae	Laurus_nobilis	AF193956
Magnoliales	Annonaceae	Asimina_triloba	AY009433
Magnoliales	Annonaceae	Polyalthia_suberosa	AF193957
Magnoliales	Eupomatiaceae	Eupomatia_laurina	AY009444
Magnoliales	Magnoliaceae	Liriodendron_tulipifera	AF193959
Magnoliales	Magnoliaceae	Magnolia_grandiflora	AF020568

Magnoliales	Magnoliaceae	Magnolia_stellata	U77622
Magnoliales	Myristicaceae	Knema_latericia	AJ223430
Magnoliales	Myristicaceae	Myristica_fragrans	AJ223434
Piperales	Aristolochiaceae	Aristolochia_elegans	AY009431
Piperales	Aristolochiaceae	Asarum_canadense	AY009432
Piperales	Lactoridaceae	Lactoris_fernandeziana	AY009446
Piperales	Piperaceae	Peperomia_cubensis	AF029783
Piperales	Piperaceae	Peperomia_polybotrya	X94594
Piperales	Piperaceae	Piper_bicolor	AY009448
Piperales	Saururaceae	Saururus_chinensis	AY009452
Chloranthales			
Chloranthales	Chloranthaceae	Chloranthus_spicatus	AY009439
Chloranthales	Chloranthaceae	Sarcandra_grandifolia	AF193958
Monocots			
Acorales	Acoraceae	Acorus_calamus	AF193944
Alismatales	Alismataceae	Alisma_sp. (CL Parkinson)	unpublished
Alismatales	Araceae	Amorphophallus_konjac	AJ007548
Alismatales	Araceae	Anthurium_scherzerianum	AJ007551
Alismatales	Araceae	Arisaema_triphyllum	AY009454
Alismatales	Araceae	Dieffenbachia_sp._Qiu_96007	AJ007549
Alismatales	Araceae	Orontium_aquaticum	AJ007554
Alismatales	Araceae	Peltandra_virginica	AJ007550
Alismatales	Araceae	Philodendron_oxycardium	AJ223438
Alismatales	Araceae	Pistia_stratiotes	AJ007546
Alismatales	Araceae	Scindapsus_aureus	AJ007552
Alismatales	Araceae	Spathiphyllum_wallisii	AJ007553
Alismatales	Araceae	Xanthosoma_mafaffa	AJ223807
Alismatales	Araceae	Zamioculcas_zamiifolia	AJ007547
Alismatales	Araceae	Zantedeschia_aethiopica	AJ007555
Arecales	Arecaceae	Chamaerops_humilis	U77621
Arecales	Arecaceae	Phoenix_dactylifera	AY166800
Arecales	Arecaceae	Sabal_palmetto	U77624
Asparagales	Alliaceae	Allium_sativum	AF356823
Dioscoreales	Dioscoreaceae	Dioscorea_mexicana	AY009442
Poales	Eriocaulaceae	Lachnocaulon_anceps	AY053574
Poales	Poaceae	Aegilops_columnaris	U46764
Poales	Poaceae	Coix_lacryma-jobi	DQ646384
Poales	Poaceae	Elymus_sibiricus	AJ296026
Poales	Poaceae	Oryza_sativa	BA000029
Poales	Poaceae	Secale_cereale	AM050161
Poales	Poaceae	Sorghum_bicolor	M14453
Poales	Poaceae	Triticum_aestivum	AP008982
Poales	Poaceae	Zea_mays	AY506529
Zingiberales	Cannaceae	Canna_paniculata	AY673037
Zingiberales	Costaceae	Costus_pulverulentus	AY673038
Zingiberales	Heliconiaceae	Heliconia_irrasa	AY673039
Zingiberales	Lowiaceae	Orchidantha_fimbriata	AY673041
Zingiberales	Marantaceae	Ataenidia_conferta	AY673012
Zingiberales	Marantaceae	Calathea_crotalifera	AY673013
Zingiberales	Marantaceae	Calathea_metallica	AY673014
Zingiberales	Marantaceae	Calathea_micans	AY673015
Zingiberales	Marantaceae	Calathea_rufibarba	AY673016
Zingiberales	Marantaceae	Calathea_warscewiczii	AY673017
Zingiberales	Marantaceae	Cominsia_gigantea	AY673018
Zingiberales	Marantaceae	Ctenanthe_setosa	AY673019
Zingiberales	Marantaceae	Donax_canniformis	AY673020
Zingiberales	Marantaceae	Haumania_sp._Harris_6672	AY673021
Zingiberales	Marantaceae	Hypselodelphys_sp._Harris_6670	AY673022

Zingiberales	Marantaceae	Ischnosiphon_helenae	AY673023
Zingiberales	Marantaceae	Maranta_bicolor	AY673024
Zingiberales	Marantaceae	Maranta_leuconeura	AJ223432
Zingiberales	Marantaceae	Marantochloa_purpurea	AY673025
Zingiberales	Marantaceae	Monotagma_laxum	AY673026
Zingiberales	Marantaceae	Phrynium_imbricatum	AY673027
Zingiberales	Marantaceae	Pleiostachya_pruinosa	AY673029
Zingiberales	Marantaceae	Saranthe_sp._Kress_96-5737	AY673030
Zingiberales	Marantaceae	Sarcophrynium_brachystachys	AY673031
Zingiberales	Marantaceae	Schumannianthus_dichotomus	AY673032
Zingiberales	Marantaceae	Schumannianthus_virgatus	AY673033
Zingiberales	Marantaceae	Stachyphrynium_repens	AY673028
Zingiberales	Marantaceae	Thalia_geniculata	AY673034
Zingiberales	Marantaceae	Thaumatococcus_daniellii	AY673035
Zingiberales	Marantaceae	Trachyphrynium_braunianum	AY673036
Zingiberales	Musaceae	Musa_nana	AJ247609
Zingiberales	Musaceae	Musella_lasiocarpa	AY673040
Zingiberales	Strelitziaceae	Phenakospermum_guyanense	AY673042
Zingiberales	Zingiberaceae	Hedychium_coronarium	AJ223426
Zingiberales	Zingiberaceae	Siphonochilus_decorus	AY673043
Ceratophyllales			
Ceratophyllales	Ceratophyllaceae	Ceratophyllum_demersum	AY009438
Stem Angiosperms			
Amborellales	Amborellaceae	Amborella_trichopoda	AY009430
Austrobaileyales	Austrobaileyaceae	Austrobaileya_scandens	AY009434
Austrobaileyales	Schisandraceae	Illicium_lanceolatum	AY009445
Austrobaileyales	Schisandraceae	Kadsura_japonica	AF193952
Austrobaileyales	Schisandraceae	Schisandra_henryi	AY009453
Austrobaileyales	Schisandraceae	Schisandra_sphenanthera	AF193951
Austrobaileyales	Trimeniaceae	Trimenia_sp._CCWD-2000	AY009456
Nymphaeales	Cabombaceae	Cabomba_caroliniana	AY009435
Nymphaeales	Cabombaceae	Cabomba_sp._Palmer_688	AF193949
Nymphaeales	Nymphaeaceae	Euryale_sp._Palmer_790	AF193947
Nymphaeales	Nymphaeaceae	Nuphar_sp._Palmer_689	AF193948
Nymphaeales	Nymphaeaceae	Nymphaea_odorata	AF020570
Nymphaeales	Nymphaeaceae	Victoria_cf._amazonica	AF193946
Gymnosperms			
Coniferales	Araucariaceae	Agathis_australis	AF020557
Coniferales	Araucariaceae	Araucaria_heterophylla	AF020558
Coniferales	Cephalotaxaceae	Cephalotaxus_fortunei	AF020559
Coniferales	Cupressaceae	Cunninghamia_konishii	AF020560
Coniferales	Cupressaceae	Cupressus_corneyana	AF020561
Coniferales	Cupressaceae	Juniperus_procera	AJ000357
Coniferales	Cupressaceae	Juniperus_virginiana	AF020567
Coniferales	Cupressaceae	Metasequoia_glyptostroboides	AF020569
Coniferales	Cupressaceae	Taxodium_distichum	AF020578
Coniferales	Cupressaceae	Thuja_plicata	S48153
Coniferales	Pinaceae	Abies_balsamea	AY159838
Coniferales	Pinaceae	Abies_bracteata	AF020556
Coniferales	Pinaceae	Larix_decidua	DQ087872
Coniferales	Pinaceae	Larix_gmelinii	DQ087877
Coniferales	Pinaceae	Larix_griffithiana	DQ087879
Coniferales	Pinaceae	Larix_kaempferi	DQ087873
Coniferales	Pinaceae	Larix_laricina	DQ087871
Coniferales	Pinaceae	Larix_lyallii	DQ087875
Coniferales	Pinaceae	Larix_mastersiana	DQ087878
Coniferales	Pinaceae	Larix_occidentalis	DQ087876
Coniferales	Pinaceae	Larix_potaninii	DQ087880

Coniferales	Pinaceae	Larix_sibirica	DQ087874
Coniferales	Pinaceae	Larix_sp.	X94590
Coniferales	Pinaceae	Picea_abies	AJ000356
Coniferales	Pinaceae	Pinus_banksiana	AY159844
Coniferales	Pinaceae	Pinus_sibirica	AJ000355
Coniferales	Pinaceae	Pinus_strobus	AF020574
Coniferales	Pinaceae	Pinus_sylvestris	AJ000354
Coniferales	Pinaceae	Pseudotsuga_menziesii	AY159841
Coniferales	Pinaceae	Tsuga_canadensis	AF020582
Coniferales	Podocarpaceae	Phyllocladus_trichomanoides	AF020573
Coniferales	Podocarpaceae	Podocarpus_macrophyllus	AF020575
Coniferales	Podocarpaceae	Podocarpus_oleifolius	AY159842
Coniferales	Sciadopityaceae	Sciadopitys_verticillata	AF020576
Coniferales	Taxaceae	Taxus_baccata	AF020579
Coniferales	Taxaceae	Taxus_canadensis	AY159840
Coniferales	Taxaceae	Torreya_nucifera	AF020580
Cycadales	Cycadaceae	Cycas_revoluta	AF020562
Cycadales	Stangeriaceae	Stangeria_eriopus	AF020577
Cycadales	Zamiaceae	Encephalartos_lebomboensis	AF020563
Cycadales	Zamiaceae	Zamia_furfuracea	AF020583
Cycadales	Zamiaceae	Zamia_sp.	X94596
Ephedrales	Ephedraceae	Ephedra_distachya	AF020585
Ephedrales	Ephedraceae	Ephedra_equisetifolia	X94586
Ephedrales	Ephedraceae	Ephedra_viridis	AF020564
Ginkgoales	Ginkgoaceae	Ginkgo_biloba	AF020565
Gnetales	Gnetaceae	Gnetum_leyboldii	AF020566
Welwitschiales	Welwitschiaceae	Welwitschia_mirabilis	AF020584

Table S4. Taxon information for figure 3

Asterids			
Apiales	Apiaceae	Petroselinum_crispum	AY453081
Apiales	Griseliniaceae	Griselinia_racemosa	AY453096
Apiales	Pittosporaceae	Pittosporum_glabratum	AF520127
Aquifoliales	Aquifoliaceae	Ilex_aquifolium	AY453090
Aquifoliales	Aquifoliaceae	Ilex_repanda	AY725885
Asterales	Asteraceae	Helianthus_annuus	AY453114
Asterales	Campanulaceae	Lobelia_holstii	AF520151
Asterales	Goodeniaceae	Scaevola_aemula	AY453118
Cornales	Cornaceae	Alangium_chinense	AF520122
Cornales	Cornaceae	Cornus_sericea	AY725883
Cornales	Cornaceae	Cornus_suecica	AF420990
Cornales	Hydrangeaceae	Deutzia_amurensis	AF520126
Cornales	Hydrangeaceae	Hydrangea_macrophylla	AY453091
Cornales	Hydrangeaceae	Hydrangea_sp._JS-2005	AY725882
Dipsacales	Caprifoliaceae	Lonicera_morrowii	AY453088
Dipsacales	Dipsacaceae	Dipsacus_fullonum	AY453093
Ericales	Actinidiaceae	Actinidia_arguta	AF420991
Ericales	Actinidiaceae	Actinidia_rubricaulis	AY163745
Ericales	Balsaminaceae	Impatiens_auriculata	AF520117
Ericales	Balsaminaceae	Impatiens_parviflora	AF421011
Ericales	Clethraceae	Clethra_alnifolia	AF520204
Ericales	Clethraceae	Clethra_arborea	AF420996
Ericales	Clethraceae	Clethra_barbinervis	AF420997
Ericales	Clethraceae	Clethra_delavayi_var._yuiana	AY163746
Ericales	Cyrillaceae	Cyrilla_racemiflora	AY725892
Ericales	Diapensiaceae	Berneuxia_thibetica	AY049792
Ericales	Diapensiaceae	Diapensia_lapponica	AF421001
Ericales	Diapensiaceae	Galax_urceolata	AF421007
Ericales	Diapensiaceae	Schizocodon_soldanelloides	AF421030
Ericales	Diapensiaceae	Shortia_exappendiculata	AF421031
Ericales	Diapensiaceae	Shortia_uniflora	AY725895
Ericales	Ebenaceae	Diospyros_digyna	AF421002
Ericales	Ebenaceae	Diospyros_mollifolia	AF520202
Ericales	Ebenaceae	Lissocarpa_guianensis	AF421012
Ericales	Ericaceae	Chimaphila_umbellata	AF420994
Ericales	Ericaceae	Empetrum_nigrum	AF520113
Ericales	Ericaceae	Enkianthus_campanulatus	AF421004
Ericales	Ericaceae	Pieris_formosa	AF520123
Ericales	Ericaceae	Rhododendron_tomentosum	AF421026
Ericales	Ericaceae	Vaccinium_uliginosum	AF421035
Ericales	Ericaceae	Vaccinium_vitis-idaea	AF520124
Ericales	Fouquieriaceae	Fouquieria_columnaris	AY725887
Ericales	Fouquieriaceae	Fouquieria_sp._Anderberg_s.n.	AF421006
Ericales	Lecythidaceae	Barringtonia_asiatica	AF420992
Ericales	Lecythidaceae	Barringtonia_racemosa	AY289672
Ericales	Lecythidaceae	Couroupita_guianensis	AY725890
Ericales	Lecythidaceae	Napoleona_imperialis	AF421041
Ericales	Lecythidaceae	Napoleona_sp._JS-2005	AY725891
Ericales	Maesaceae	Maesa_tenera	AF421015
Ericales	Marcgraviaceae	Marcgravia_sp._Anderberg_s.n.	AF421017
Ericales	Marcgraviaceae	Norantea_peduncularis	AF421020
Ericales	Mitrastemonaceae	Mitrastema_yamamotoi	AY739011
Ericales	Myrsinaceae	Aegiceras_corniculatum	AY289673
Ericales	Myrsinaceae	Ardisia_crenata	AF520206
Ericales	Myrsinaceae	Lysimachia_vulgaris	AF421013
Ericales	Myrsinaceae	Myrsine_africana	AF421019

Ericales	Pellicieraceae	Pelliciera_rhizophorae	AF421022
Ericales	Pentaphylacaceae	Pentaphylax_euryoides	AF520096
Ericales	Polemoniaceae	Acanthogilia_gloriosa	AY725888
Ericales	Polemoniaceae	Cantua_buxifolia	AY725889
Ericales	Polemoniaceae	Cobaea_scandens	AF420999
Ericales	Polemoniaceae	Polemonium_caeruleum	AF421023
Ericales	Primulaceae	Primula_sikkimensis	AF421025
Ericales	Primulaceae	Primula_sp._YJB-2002	AF520114
Ericales	Roridulaceae	Roridula_gorgonias	AF421036
Ericales	Sapotaceae	Eberhardtia_aurata	AF520116
Ericales	Sapotaceae	Eberhardtia_tonkinensis	AY163750
Ericales	Sapotaceae	Madhuca_microphylla	AF421014
Ericales	Sapotaceae	Manilkara_zapota	AF421016
Ericales	Sapotaceae	Monotheca_buxifolia	AF421018
Ericales	Sapotaceae	Palaquium_formosanum	AF421021
Ericales	Sapotaceae	Planchonella_obovata	AF421024
Ericales	Sapotaceae	Sarcosperma_laurinum	AF421039
Ericales	Sarraceniaceae	Darlingtonia_californica	AY163751
Ericales	Sarraceniaceae	Heliamphora_sp._Anderberg_s.n.	AF421010
Ericales	Sarraceniaceae	Sarracenia_flava	AF421028
Ericales	Sladeniaceae	Ficalhoa_laurifolia	AF421037
Ericales	Sladeniaceae	Sladenia_celastrifolia	AF520115
Ericales	Styracaceae	Bruinsmia_styracoides	AY725894
Ericales	Styracaceae	Halesia_carolina	AF421009
Ericales	Styracaceae	Styrax_grandiflorus	AF520205
Ericales	Styracaceae	Styrax_officinalis	AF421032
Ericales	Symplocaceae	Symplocos_hookeri	AY163753
Ericales	Symplocaceae	Symplocos_setchuensis	AF520111
Ericales	Symplocaceae	Symplocos_sp._Chung_and_Anderberg_1351	AF421038
Ericales	Symplocaceae	Symplocos_zizyphoides	AY725893
Ericales	Ternstroemiaceae	Adinandra_hirta	AY163739
Ericales	Ternstroemiaceae	Anneslea_fragrans	AY163734
Ericales	Ternstroemiaceae	Archytaea_multiflora	AY674475
Ericales	Ternstroemiaceae	Cleyera_japonica	AF420998
Ericales	Ternstroemiaceae	Cleyera_pachyphylla	AY163737
Ericales	Ternstroemiaceae	Eurya_handel-mazzettii	AY163748
Ericales	Ternstroemiaceae	Eurya_sp._Chung_&_Anderberg_1406	AF421005
Ericales	Ternstroemiaceae	Euryodendron_excelsum	AY163733
Ericales	Ternstroemiaceae	Ternstroemia_gymnanthera	AY163754
Ericales	Tetrameristaceae	Pentamerista_neotropica	AY725886
Ericales	Theaceae	Apterosperma_oblata	AY163755
Ericales	Theaceae	Camellia_henryana_var._trichocarpa	AY163729
Ericales	Theaceae	Camellia_sinensis	AF421034
Ericales	Theaceae	Camellia_sp._YJB-2002	AF520203
Ericales	Theaceae	Camellia_yunnanensis_var._camellioides	AY163744
Ericales	Theaceae	Franklinia_alatamaha	AY163731
Ericales	Theaceae	Gordonia_axillaris	AF421008
Ericales	Theaceae	Gordonia_lasianthus	AY163735
Ericales	Theaceae	Parapyrenaria_multisepala	AY163742
Ericales	Theaceae	Polyspora_chrysandra	AY163741
Ericales	Theaceae	Polyspora_tonkinensis	AY163728
Ericales	Theaceae	Schima_khasiana	AY163740
Ericales	Theaceae	Schima_superba	AF421029
Ericales	Theaceae	Sinopyrenaria_yunnanensis	AY163730
Ericales	Theaceae	Stewartia_gemmata	AY163732
Ericales	Theaceae	Stewartia_pteropetiolata	AY163738
Ericales	Theaceae	Stewartia_serrata	AY163736
Ericales	Theaceae	Tutcheria_spectabilis	AY163743

Ericales	Theophrastaceae	Clavija_domingensis	AF420995
Ericales	Theophrastaceae	Samolus_repens	AF421027
Garryales	Aucubaceae	Aucuba_himalaica	AY453099
Garryales	Aucubaceae	Aucuba_japonica	AY725884
Garryales	Eucommiaceae	Eucommia_ulmoides	AF520125
Garryales	Garryaceae	Garrya_elliptica	AY453095
Gentianales	Apocynaceae	Asclepias_tuberosa	AY453103
Gentianales	Apocynaceae	Cerbera_manghas	AY289670
Gentianales	Apocynaceae	Gomphocarpus_physocarpus	AF520144
Gentianales	Apocynaceae	Vinca_minor	AF520143
Gentianales	Rubiaceae	Pentas_sp._Barkman_362	AY453079
Gentianales	Rubiaceae	Scyphiphora_hydrophyllacea	AY289671
Lamiales	Acanthaceae	Acanthus_ebracteatus	AY289667
Lamiales	Acanthaceae	Avicennia_marina	AY289666
Lamiales	Acanthaceae	Blepharis_hildebrandtii	AF520153
Lamiales	Bignoniaceae	Dolichandrone_spathacea	AY289668
Lamiales	Bignoniaceae	Jacaranda_mimosifolia	AF520145
Lamiales	Lamiaceae	Clerodendrum_inerme	AY289669
Lamiales	Lamiaceae	Monarda_didyma	AY453085
Lamiales	Oleaceae	Jasminum_abyssinicum	AF520152
Lamiales	Plantaginaceae	Antirrhinum_majus	AY453102
Lamiales	Plantaginaceae	Plantago_lanceolata	unpubl. (JPM)
Solanales	Solanaceae	Nicotiana_sylvestris	AY453113
Solanales	Solanaceae	Nicotiana_tabacum	BA000042
Solanales	Solanaceae	Solanum_tuberosum	AJ003130
Solanales	Solanaceae	Withania_somnifera	AF520149
Unplaced	Icacinaceae	Mappianthus_iodoides	AF520167
Caryophyllids			
Caryophyllales	Amaranthaceae	Bassia_scoparia	AF520130
Caryophyllales	Amaranthaceae	Beta_vulgaris_subsp._vulgaris	BA000009
Caryophyllales	Amaranthaceae	Spinacia_oleracea	AY453110
Caryophyllales	Basellaceae	Basella_alba	AF520150
Caryophyllales	Didiereaceae	Alluaudia_humbertii	AF520129
Caryophyllales	Nepenthaceae	Nepenthes_mirabilis	AF520128
Caryophyllales	Nyctaginaceae	Mirabilis_jalapa	AY453086
Caryophyllales	Plumbaginaceae	Aegialitis_annulata	AY289665
Caryophyllales	Portulacaceae	Portulacaria_afra	AF520131
Caryophyllales	Tamaricaceae	Tamarix_chinensis	AF520098
Caryophyllales	Tamaricaceae	Tamarix_parviflora	AY453117
Dilleniales	Dilleniaceae	Dillenia_indica	AF520095
Dilleniales	Dilleniaceae	Tetracera_asiatica	AF520094
Rosids			
Brassicales	Brassicaceae	Arabidopsis_thaliana	Y08501
Brassicales	Brassicaceae	Brassica_napus	AP006444
Brassicales	Brassicaceae	Capsella_bursa-pastoris	AF520121
Brassicales	Bretschneideraceae	Bretschneidera_sinensis	AF520118
Brassicales	Capparaceae	Capparis_membranifolia	AF520146
Brassicales	Caricaceae	Carica_papaya	AF520141
Brassicales	Tropaeolaceae	Tropaeolum_majus	AF520137
Celastrales	Celastraceae	Brexia_madagascariensis	AY674482
Celastrales	Celastraceae	Celastrus_orbiculatus	AY121493
Celastrales	Celastraceae	Celastrus_scandens	AY674488
Celastrales	Celastraceae	Elaeodendron_orientale	AY674506
Celastrales	Celastraceae	Euonymus_alatus	AY674511
Celastrales	Celastraceae	Euonymus_bungeanus	AY121492
Celastrales	Celastraceae	Euonymus_fortunei	AY453104
Celastrales	Celastraceae	Maytenus_arbutifolia	AY674538
Celastrales	Celastraceae	Paxistima_canbyi	AY674548

Celastrales	Celastraceae	Salacia_sessiliflora	AY121503
Celastrales	Celastraceae	Stackhousia_minima	AY121497
Celastrales	Celastraceae	Tripterococcus_brunonis	AY121496
Celastrales	Celastraceae	Tripterygium_regelii	AY674568
Celastrales	Parnassiaceae	Parnassia_palustris	AY121491
Celastrales	Parnassiaceae	Parnassia_sp._Wurdack_D795	AY674546
Crossosomatales	Stachyuraceae	Stachyurus_chinensis	AY121489
Crossosomatales	Staphyleaceae	Euscaphis_japonica	AF520104
Crossosomatales	Staphyleaceae	Staphylea_trifolia	AF520105
Cucurbitales	Anisophylleaceae	Anisophyllea_manausensis	AY121498
Cucurbitales	Apodanthaceae	Apodanthes_caseariae	AY739002
Cucurbitales	Apodanthaceae	Pilostyles_thurberi	AY739003
Cucurbitales	Begoniaceae	Begonia_hybrid_cultivar	AY453119
Cucurbitales	Begoniaceae	Begonia_laciniata	AY121500
Cucurbitales	Coriariaceae	Coriaria_nepalensis	AY121501
Cucurbitales	Corynocarpaceae	Corynocarpus_laevigata	AY121499
Cucurbitales	Cucurbitaceae	Cucurbita_pepo	AY453101
Cucurbitales	Cucurbitaceae	Hemsleya_lijiangensis	AF520174
Cucurbitales	Cucurbitaceae	Luffa_aegyptiaca	AF520173
Cucurbitales	Cucurbitaceae	Thladiantha_dubia	AF520175
Cucurbitales	Tetramelaceae	Tetrameles_nudiflora	AF520172
Fabales	Fabaceae	Albizia_schimperiana	AF520168
Fabales	Fabaceae	Cynometra_iripa	AY289656
Fabales	Fabaceae	Glycine_max	U09988
Fabales	Fabaceae	Glycine_soja	AF520177
Fabales	Fabaceae	Julbernardia_globiflora	AY739016
Fabales	Fabaceae	Pisum_sativum	AY453078
Fabales	Fabaceae	Pongamia_pinnata	AY289655
Fabales	Fabaceae	Thermopsis_chinensis	AF520176
Fabales	Fabaceae	Vicia_faba	M30176
Fabales	Fabaceae	Zenia_insignis	AF520161
Fabales	Polygalaceae	Polygala_paucifolia	AY453080
Fabales	Polygalaceae	Polygala_tatarinowii	AF520179
Fabales	Polygalaceae	Xanthophyllum_hainanense	AF520178
Fagales	Betulaceae	Alnus_henryi	AF520062
Fagales	Betulaceae	Alnus_sinuata	AY263907
Fagales	Betulaceae	Betula_albosinensis	AF520063
Fagales	Betulaceae	Betula_pendula	AY453121
Fagales	Betulaceae	Betula_platyphylla	AY263908
Fagales	Betulaceae	Carpinus_betulus	AY263910
Fagales	Betulaceae	Carpinus_polyneura	AF520065
Fagales	Betulaceae	Corylus_avellana	AF520067
Fagales	Betulaceae	Ostrya_carpinifolia	AF520064
Fagales	Betulaceae	Ostryopsis_davidiana	AF520066
Fagales	Casuarinaceae	Casuarina_equisetifolia	AF520069
Fagales	Fagaceae	Castanea_seguinii	AF520078
Fagales	Fagaceae	Castanopsis_tibetana	AY263912
Fagales	Fagaceae	Chrysolepis_sempervirens	AY147114
Fagales	Fagaceae	Fagus_engleriana	AF520082
Fagales	Fagaceae	Fagus_grandifolia	AY263909
Fagales	Fagaceae	Fagus_sylvatica	AY453092
Fagales	Fagaceae	Lithocarpus_henryi	AF520081
Fagales	Fagaceae	Quercus_engleriana	AF520079
Fagales	Fagaceae	Quercus_multinervis	AY263911
Fagales	Fagaceae	Trigonobalanus_verticillata	AF520080
Fagales	Juglandaceae	Alfaroa_guanacastensis	AF520076
Fagales	Juglandaceae	Annamocarya_sinensis	AY263913
Fagales	Juglandaceae	Carya_ovata	AF520070

Fagales	Juglandaceae	Cyclocarya_paliurus	AF520071
Fagales	Juglandaceae	Engelhardia_fenzelii	AF520072
Fagales	Juglandaceae	Juglans_mandshurica	AF520073
Fagales	Juglandaceae	Oreomunnea_mexicana	AF520083
Fagales	Juglandaceae	Platycarya_strobilacea	AF520074
Fagales	Juglandaceae	Pterocarya_hupehensis	AF520075
Fagales	Myricaceae	Comptonia_peregrina	AY147115
Fagales	Myricaceae	Morella_cerifera	AF520084
Fagales	Nothofagaceae	Nothofagus_dombeyi	AF520085
Fagales	Rhoipteleaceae	Rhoiptelea_chiliantha	AF520077
Fagales	Ticodendraceae	Ticodendron_incognitum	AF520068
Geraniales	Geraniaceae	Geranium_wilfordii	AY121488
Geraniales	Melianthaceae	Melianthus_major	AY453087
Huerteales	Dipentodontaceae	Dipentodon_sinicus	AY121494
Huerteales	Tapisciaceae	Tapiscia_sinensis	AF520103
Malpighiales	Achariaceae	Acharia_tragodes	AY674472
Malpighiales	Achariaceae	Kiggelaria_sp._Alford_51	AY674527
Malpighiales	Balanopaceae	Balanops_vieillardii	AF520180
Malpighiales	Caryocaraceae	Caryocar_glabrum	AF520181
Malpighiales	Chrysobalanaceae	Atuna_racemosa	AY674476
Malpighiales	Chrysobalanaceae	Chrysobalanus_icaco	AY674491
Malpighiales	Chrysobalanaceae	Couepia_robusta	AF520182
Malpighiales	Chrysobalanaceae	Hirtella_bicornis	AY674520
Malpighiales	Chrysobalanaceae	Licania_michauxii	AY674532
Malpighiales	Clusiaceae	Calophyllum_soulattri	AY674484
Malpighiales	Clusiaceae	Clusia_gundlachii	AY674493
Malpighiales	Clusiaceae	Hypericum_ascyron	AF520190
Malpighiales	Clusiaceae	Hypericum_empetrifolium	AY674525
Malpighiales	Clusiaceae	Mesua_ferrea	AF520183
Malpighiales	Clusiaceae	Mesua_sp._Coode_7884	AY674540
Malpighiales	Clusiaceae	Vismia_sp._Miller_et_al._9313	AY674571
Malpighiales	Ctenolophonaceae	Ctenolophon_englerianus	AY674499
Malpighiales	Dichapetalaceae	Dichapetalum_macrocarpum	AY674502
Malpighiales	Elatinaceae	Bergia_texana	AY674480
Malpighiales	Elatinaceae	Elatine_triandra	AY674507
Malpighiales	Erythroxylaceae	Erythroxylum_coca	AY674509
Malpighiales	Euphorbiaceae	Clutia_pulchella	AY674494
Malpighiales	Euphorbiaceae	Codiaeum_variegatum	AY674495
Malpighiales	Euphorbiaceae	Conceveiba_martiana	AY674496
Malpighiales	Euphorbiaceae	Croton_alabamensis_var._alabamensis	AY674498
Malpighiales	Euphorbiaceae	Endospermum_moluccanum	AY674508
Malpighiales	Euphorbiaceae	Euphorbia_polychroma	AY674512
Malpighiales	Euphorbiaceae	Excoecaria_agallocha	AY289654
Malpighiales	Euphorbiaceae	Hevea_sp._Gillespie_4272	AY674519
Malpighiales	Euphorbiaceae	Homalanthus_populneus	AY674521
Malpighiales	Euphorbiaceae	Neoscortechinia_kingii	AY674543
Malpighiales	Euphorbiaceae	Pera_bicolor	AY674549
Malpighiales	Euphorbiaceae	Ricinus_communis	AY674560
Malpighiales	Euphroniaceae	Euphronia_guianensis	AY674513
Malpighiales	Goupiaceae	Goupia_glabra	AY674516
Malpighiales	Humiriaceae	Humiria_balsamifera	AY674523
Malpighiales	Humiriaceae	Vantanea_guianensis	AY674570
Malpighiales	Ixonanthaceae	Ixonanthes_chinensis	AY674526
Malpighiales	Ixonanthaceae	Ochthocosmus_longipedicellatus	AY674545
Malpighiales	Lacistemataceae	Lacistema_aggregatum	AY674529
Malpighiales	Linaceae	Hugonia_platysepala	AY674522
Malpighiales	Linaceae	Linum_arboreum	AY674533
Malpighiales	Linaceae	Reinwardtia_indica	AF520164

Malpighiales	Lophopyxidaceae	Lophopyxis_maingayi	AY674534
Malpighiales	Malesherbiaceae	Malesherbia_linearifolia	AY674536
Malpighiales	Malpighiaceae	Dicella_nucifera	AY674501
Malpighiales	Malpighiaceae	Malpighia_glauca	AF520187
Malpighiales	Malpighiaceae	Thryallis_longifolia	AY674566
Malpighiales	Medusagynaceae	Medusagyne_oppositifolia	AY674539
Malpighiales	Ochnaceae	Cespedesia_bonplandii	AY674490
Malpighiales	Ochnaceae	Ochna_integerrima	AF520166
Malpighiales	Ochnaceae	Ochna_sp._Davis_31-01	AY674544
Malpighiales	Pandaceae	Galearia_filiformis	AY674515
Malpighiales	Pandaceae	Microdesmis_puberula	AY674542
Malpighiales	Passifloraceae	Paropsia_madagascariensis	AY674547
Malpighiales	Passifloraceae	Passiflora_edulis	AF520188
Malpighiales	Phyllanthaceae	Bischofia_javanica	AF520184
Malpighiales	Phyllanthaceae	Drypetes_perreticulata	AF520185
Malpighiales	Phyllanthaceae	Phyllanthus_epiphyllanthus	AY674552
Malpighiales	Phyllanthaceae	Putranjiva_roxburghii	AY674505
Malpighiales	Picrodendraceae	Androstachys_johnsonii	AY674474
Malpighiales	Picrodendraceae	Austrobuxus_megacarpus	AY674477
Malpighiales	Picrodendraceae	Dissiliaria_muelleri	AY674503
Malpighiales	Picrodendraceae	Micrantheum_hexandrum	AY674541
Malpighiales	Picrodendraceae	Petalostigma_pubescens	AY674551
Malpighiales	Picrodendraceae	Podocalyx_loranthoides	AY674553
Malpighiales	Picrodendraceae	Stachystemon_axillaris	AY674563
Malpighiales	Podostemaceae	Marathrum_cf._oxycarpum_da_Cachoeirc_9/1996	AY674537
Malpighiales	Quiinaceae	Quiina_pteridophylla	AY674558
Malpighiales	Rafflesiaceae	Rafflesia_keithii	AY739007
Malpighiales	Rafflesiaceae	Rafflesia_pricei	AY739008
Malpighiales	Rafflesiaceae	Rafflesia_tuan-mudae	AY739009
Malpighiales	Rafflesiaceae	Rhizanthes_infanticida	AY739010
Malpighiales	Rafflesiaceae	Rhizanthes_zippelii	AY453073
Malpighiales	Rafflesiaceae	Sapria_himalayana	AY739006
Malpighiales	Rafflesiaceae	Sapria_poilanei	AY739004
Malpighiales	Rafflesiaceae	Sapria_ram	AY739005
Malpighiales	Rhizophoraceae	Bruguiera_gymnorhiza	AY674483
Malpighiales	Rhizophoraceae	Bruguiera_sexangula	AY289652
Malpighiales	Rhizophoraceae	Carallia_brachiata	AF520189
Malpighiales	Rhizophoraceae	Ceriops_tagal	AY289650
Malpighiales	Rhizophoraceae	Kandelia_candel	AY289651
Malpighiales	Rhizophoraceae	Rhizophora_stylosa	AY289653
Malpighiales	Salicaceae	Abatia_parviflora	AY674471
Malpighiales	Salicaceae	Casearia_sylvestris	AY674487
Malpighiales	Salicaceae	Dovyalis_rhamnoides	AY674504
Malpighiales	Salicaceae	Flacourtia_jangomas	AY674514
Malpighiales	Salicaceae	Flacourtia_ramontchii	AF520186
Malpighiales	Salicaceae	Hasseltia_sp._Alford_28	AY674518
Malpighiales	Salicaceae	Lunania_sp._Alford_69	AY674535
Malpighiales	Salicaceae	Poliothyrsis_sp._Alford_44	AY674555
Malpighiales	Salicaceae	Populus_maximowiczii	AY674556
Malpighiales	Salicaceae	Prockia_sp._Alford_85	AY674557
Malpighiales	Salicaceae	Salix_babylonica	AY453072
Malpighiales	Salicaceae	Salix_raddeana	AF520191
Malpighiales	Salicaceae	Scyphostegia_borneensis	AY674562
Malpighiales	Trigoniaceae	Trigonia_nivea	AY674567
Malpighiales	Turneraceae	Turnera_ulmifolia	AY674569
Malpighiales	Violaceae	Hybanthus_sp._Alford_89	AY674524
Malpighiales	Violaceae	Leonia_glycycarpa	AY674531
Malpighiales	Violaceae	Viola_acuminata	AF520192

Malpighiales	Violaceae	Viola_cucullata	AY453070
Malvales	Bixaceae	Bixa_orellana	AF520136
Malvales	Cistaceae	Hudsonia_tomentosa	AY453097
Malvales	Cytinaceae	Bdallophytum_americanum	AY739012
Malvales	Cytinaceae	Cytinus_ruber	AY739013
Malvales	Dipterocarpaceae	Hopea_hainanensis	AF520138
Malvales	Dipterocarpaceae	Vatica_mangachapoi	AF520132
Malvales	Malvaceae	Abutilon_x_hybridum	AY739014
Malvales	Malvaceae	Alcea_rosea	AY453094
Malvales	Malvaceae	Bombax_malabaricum	AF520148
Malvales	Malvaceae	Excentrodendron_hsienmu	AF520158
Malvales	Malvaceae	Heritiera_littoralis	AY289659
Malvales	Malvaceae	Hibiscus_tiliaceus	AY289657
Malvales	Malvaceae	Pavonia_spinifex	AY739015
Malvales	Malvaceae	Sida_cordata	AF520139
Malvales	Malvaceae	Thespesia_populnea	AY289658
Malvales	Malvaceae	Tilia_mandshurica	AF520107
Malvales	Thymelaeaceae	Aquilaria_sinensis	AF520171
Myrtales	Combretaceae	Conocarpus_erectus	AY289662
Myrtales	Combretaceae	Laguncularia_racemosa	AY289661
Myrtales	Combretaceae	Lumnitzera_littorea	AY289663
Myrtales	Combretaceae	Quisqualis_indica	AF520133
Myrtales	Crypteroniaceae	Crypteronia_paniculata	AF520119
Myrtales	Lythraceae	Duabanga_grandiflora	AF520142
Myrtales	Lythraceae	Lythrum_salicaria	AF520110
Myrtales	Lythraceae	Punica_granatum	AY121502
Myrtales	Lythraceae	Sonneratia_ovata	AY289664
Myrtales	Melastomataceae	Melastoma_candidum	AF520134
Myrtales	Onagraceae	Epilobium_hirsutum	AF520108
Myrtales	Onagraceae	Oenothera_berteroana	M63034
Myrtales	Onagraceae	Oenothera_biennis	AY453083
Oxalidales	Cephalotaceae	Cephalotus_follicularis	AF520193
Oxalidales	Connaraceae	Agelaea_trinervis	AF520195
Oxalidales	Connaraceae	Rourea_minor	AF520194
Oxalidales	Cunoniaceae	Eucryphia_milliganii	AY674510
Oxalidales	Cunoniaceae	Schizomeria_serrata	AF520196
Oxalidales	Elaeocarpaceae	Crinodendron_hookerianum	AY674497
Oxalidales	Elaeocarpaceae	Elaeocarpus_apiculatus	AF520197
Oxalidales	Oxalidaceae	Averrhoa_carambola	AY674478
Oxalidales	Oxalidaceae	Dapania_racemosa	AY674500
Oxalidales	Oxalidaceae	Oxalis_corniculata	AY453111
Oxalidales	Oxalidaceae	Oxalis_corymbosa	AF520198
Rosales	Barbeyaceae	Barbeya_oleoides	AF520199
Rosales	Cannabaceae	Humulus_japonicus	AF520091
Rosales	Moraceae	Maclura_tricuspidata	AF520093
Rosales	Moraceae	Morus_alba	AY453084
Rosales	Rhamnaceae	Hovenia_acerba	AF520156
Rosales	Rhamnaceae	Paliurus_hemsleyanus	AF520200
Rosales	Rosaceae	Exochorda_racemosa	AF520159
Rosales	Rosaceae	Prinsepia_uniflora	AF520088
Rosales	Rosaceae	Taihangia_rupestris	AF520201
Rosales	Ulmaceae	Celtis_bungeana	AF520086
Rosales	Ulmaceae	Celtis_philippensis	AY263906
Rosales	Ulmaceae	Hemiptelea_davidii	AF520160
Rosales	Ulmaceae	Ulmus_americana	AY453107
Rosales	Ulmaceae	Zelkova_serrata	AF520089
Rosales	Urticaceae	Cecropia_peltata	AF520157
Rosales	Urticaceae	Debregeasia_salicifolia	AF520090

	Sapindales	Anacardiaceae	Rhus_typhina	AY453075
	Sapindales	Burseraceae	Canarium_tramdenum	AF520140
	Sapindales	Meliaceae	Aglaia_odorata	AF520147
	Sapindales	Meliaceae	Xylocarpus_granatum	AY289660
	Sapindales	Rutaceae	Citrus_sinensis	AY453100
	Sapindales	Rutaceae	Ptelea_trifoliata	AF520109
	Sapindales	Rutaceae	Skimmia_japonica	AF520162
	Sapindales	Sapindaceae	Acer_mono	AF520112
	Sapindales	Sapindaceae	Koelreuteria_bipinnata	AF520120
	Sapindales	Simaroubaceae	Ailanthus_altissima	AF520106
	Sapindales	Simaroubaceae	Brucea_javanica	AF520135
	Sapindales	Simaroubaceae	Klainedoxa_gabonensis	AY674528
	Sapindales	Simaroubaceae	Picrasma_quassioides	AF520154
	Saxifragales	Crassulaceae	Sedum_sarmentosum	AF520100
	Saxifragales	Cynomoriaceae	Cynomorium_coccineum	AY957446
	Saxifragales	Hamamelidaceae	Hamamelis_japonica_f._obtusata	AF520087
	Saxifragales	Hamamelidaceae	Hamamelis_vernalis	AY453082
	Saxifragales	Iteaceae	Itea_yunnanensis	AF520099
	Saxifragales	Paeoniaceae	Paeonia_japonica	AF520101
	Saxifragales	Peridiscaceae	Peridiscus_lucidus	AY674550
	Unplaced	Huaceae	Afrostyrax_sp._Cheek_5007	AY121495
	Unplaced	Huaceae	Hua_gabonii	AY121490
	Vitales	Vitaceae	Cissus_quadrangularis	AF520170
	Vitales	Vitaceae	Cissus_sicyoides	AY674492
	Vitales	Vitaceae	Leea_guineensis	AY674530
	Vitales	Vitaceae	Leea_indica	AF520165
	Vitales	Vitaceae	Tetrastigma_dubium	AY674565
	Vitales	Vitaceae	Tetrastigma_leucostaphylum	AY739017
	Vitales	Vitaceae	Vitis_riparia	AY453123
	Vitales	Vitaceae	Yua_thomsonii	AF520169
	Zygophyllales	Krameriaceae	Krameria_lanceolata	AY453089
	Zygophyllales	Zygophyllaceae	Guaiacum_sanctum	AY674517
	Zygophyllales	Zygophyllaceae	Kallstroemia_parviflora	AY453122
	Zygophyllales	Zygophyllaceae	Tribulus_terrestris	AF520097
Stem Eudicots				
	Buxales	Buxaceae	Buxus_sempervirens	AF197786
	Buxales	Buxaceae	Pachysandra_procumbens	AF197784
	Buxales	Buxaceae	Sarcococca_confusa	AF197785
	Buxales	Didymelaceae	Didymeles_perrieri	AF197811
	Gunnerales	Gunneraceae	Gunnera_manicata	AF520163
	Proteales	Nelumbonaceae	Nelumbo_nucifera	AF197795
	Proteales	Platanaceae	Platanus_occidentalis	AF197793
	Proteales	Proteaceae	Grevillea_robusta	AF197808
	Proteales	Proteaceae	Persoonia_katerae	AF197794
	Proteales	Proteaceae	Petrophile_canescens	AF197807
	Ranunculales	Berberidaceae	Mahonia_bealei	AF197761
	Ranunculales	Berberidaceae	Podophyllum_peltatum	AF197762
	Ranunculales	Eupteleaceae	Euptelea_polyandra	AF197787
	Ranunculales	Fumariaceae	Dicentra_sp._Qiu_95026	AF197796
	Ranunculales	Lardizabalaceae	Akebia_quinata	AF197810
	Ranunculales	Lardizabalaceae	Lardizabala_biternata	AF197789
	Ranunculales	Lardizabalaceae	Sargentodoxa_cuneata	AF197790
	Ranunculales	Menispermaceae	Cissampelos_pareira	AF197775
	Ranunculales	Menispermaceae	Cocculus_trilobus	AF197776
	Ranunculales	Papaveraceae	Sanguinaria_canadensis	AF197788
	Ranunculales	Ranunculaceae	Ranunculus_sp._Qiu_95024	AF197759
	Ranunculales	Ranunculaceae	Xanthorhiza_simplicissima	AF197760
	Sabiales	Sabiaceae	Meliosma_squamulata	DQ007426

Sabiales	Sabiaceae	Sabia_sp._Qiu_91025	AF197780
Santalales	Balanophoraceae	Dactylanthus_taylorii	AY957447
Santalales	Balanophoraceae	Hachettea_austrocaledonica	AY957448
Santalales	Balanophoraceae	Mystropetalon_thomii	AY957449
Santalales	Loranthaceae	Amyema_beccarii	AY453106
Santalales	Loranthaceae	Dendrophthoe_pentandra	AY453120
Santalales	Santalaceae	Osyris_wightiana	AF520155
Santalales	Santalaceae	Santalum_album	AY957450
Santalales	Santalaceae	Thesium_chinense	AF520102
Santalales	Schoepfiaceae	Schoepfia_schreberi	AY957451
Trochodendrales	Tetracentraceae	Tetracentron_sinense	AF197791
Trochodendrales	Trochodendraceae	Trochodendron_aralioides	AF197792
Magnoliids			
Canellales	Canellaceae	Canella_winterana	AF197757
Canellales	Canellaceae	Cinnamodendron_ekmanii	AF197758
Canellales	Winteraceae	Drimys_winteri	AF197781
Canellales	Winteraceae	Takhtajania_perrieri	DQ007427
Canellales	Winteraceae	Tasmannia_insipida	AF197782
Canellales	Winteraceae	Zygogynum_pauciflorum	AF197783
Laurales	Atherospermataceae	Atherosperma_moschatum	AF197799
Laurales	Atherospermataceae	Daphnandra_micrantha	AF197800
Laurales	Atherospermataceae	Doryphora_sassafras	AF197801
Laurales	Calycanthaceae	Calycanthus_floridus	AF197777
Laurales	Calycanthaceae	Chimonanthus_praecox	AF197778
Laurales	Calycanthaceae	Idiospermum_australiense	AF197779
Laurales	Hernandiaceae	Gyrocarpus_sp._Chase_317	AF197805
Laurales	Hernandiaceae	Hernandia_ovigera	DQ007424
Laurales	Lauraceae	Cinnamomum_camphora	AF197797
Laurales	Lauraceae	Cryptocarya_meissneriana	AF197804
Laurales	Lauraceae	Laurus_nobilis	AF197798
Laurales	Monimiaceae	Hedycarya_arborea	AF197806
Laurales	Monimiaceae	Palmeria_scandens	AF197802
Laurales	Monimiaceae	Peumus_boldus	AF197803
Laurales	Siparunaceae	Siparuna_decipiens	AF197809
Magnoliales	Annonaceae	Annona_muricata	AF197766
Magnoliales	Annonaceae	Asimina_triloba	AF197765
Magnoliales	Annonaceae	Cananga_odorata	AF197763
Magnoliales	Annonaceae	Polyalthia_suberosa	AF197764
Magnoliales	Degeneriaceae	Degeneria_vitiensis	AF197771
Magnoliales	Eupomatiaceae	Eupomatia_bennettii	AF197772
Magnoliales	Himantandraceae	Galbulimima_belgraveana	AF197773
Magnoliales	Magnoliaceae	Liriodendron_chinense	AF197774
Magnoliales	Magnoliaceae	Magnolia_tripetala	AF197770
Magnoliales	Myristicaceae	Knema_latericia	AF197767
Magnoliales	Myristicaceae	Mauloutchia_chapelieri	AF197769
Magnoliales	Myristicaceae	Myristica_fragrans	AF197768
Piperales	Aristolochiaceae	Aristolochia_macrophylla	AF197732
Piperales	Aristolochiaceae	Asarum_canadense	AF197751
Piperales	Aristolochiaceae	Saruma_henryi	AF197752
Piperales	Aristolochiaceae	Thottea_tomentosa	AF197733
Piperales	Hydnoraceae	Hydnora_africana	AF503358
Piperales	Hydnoraceae	Prosopanche_americana	AF503359
Piperales	Lactoridaceae	Lactoris_fernandeziana	AF197812
Piperales	Piperaceae	Peperomia_obtusifolia	AF197814
Piperales	Piperaceae	Piper_betle	AF197750
Piperales	Piperaceae	Zippelia_begoniifolia	AF332107
Piperales	Saururaceae	Anemopsis_californica	AF197747
Piperales	Saururaceae	Gymnotheca_chinensis	AF332103

	Piperales	Saururaceae	Gymnotheca_involucrata	AF332105
	Piperales	Saururaceae	Houttuynia_cordata	AF197749
	Piperales	Saururaceae	Saururus_cernuus	AF197748
	Piperales	Saururaceae	Saururus_chinensis	AF332106
Chloranthales				
	Chloranthales	Chloranthaceae	Ascarina_sp._Qiu-M149	AF197755
	Chloranthales	Chloranthaceae	Chloranthus_holostegius	AF332104
	Chloranthales	Chloranthaceae	Chloranthus_multistachys	AF197753
	Chloranthales	Chloranthaceae	Hedyosmum_arborescens	AF197756
	Chloranthales	Chloranthaceae	Sarcandra_chloranthoides	AF197754
Monocots				
	Acorales	Acoraceae	Acorus_calamus	DQ007422
	Acorales	Acoraceae	Acorus_gramineus	DQ007423
	Alismatales	Alismataceae	Alisma_plantago-aquatica	AF197815
	Alismatales	Araceae	Orontium_aquaticum	AF197745
	Alismatales	Araceae	Spathiphyllum_wallisii	AF197746
	Alismatales	Juncaginaceae	Triglochin_maritima	AF197725
	Alismatales	Potamogetonaceae	Potamogeton_berchtoldii	AF197724
	Alismatales	Tofieldiaceae	Pleea_tenuifolia	AF197743
	Alismatales	Tofieldiaceae	Tofieldia_calyculata	AF197744
	Arecales	Arecaceae	Caryota_mitis	AY453098
	Arecales	Arecaceae	Nypa_fruticans	AY289674
	Asparagales	Asparagaceae	Asparagus_officinalis	AF197736
	Asparagales	Ruscaceae	Polygonatum_biflorum	AY453077
	Commelinales	Commelinaceae	Tradescantia_ohiensis	AY453108
	Dioscoreales	Dioscoreaceae	Dioscorea_sp._Qiu_94044	AF197737
	Liliales	Alstroemeriaceae	Alstroemeria_hybrid_cultivar	AY453076
	Pandanales	Cyclanthaceae	Carludovica_palmata	AF197734
	Pandanales	Stemonaceae	Croomia_pauciflora	AF197735
	Poales	Poaceae	Oryza_sativa	BA000029
	Poales	Poaceae	Triticum_aestivum	AP008982
	Poales	Poaceae	Zea_mays	AY506529
	Zingiberales	Strelitziaceae	Strelitzia_reginae	AY453112
Ceratophyllales				
	Ceratophyllales	Ceratophyllaceae	Ceratophyllum_demersum	AF197730
	Ceratophyllales	Ceratophyllaceae	Ceratophyllum_submersum	AF197731
Stem Angiosperms				
	Amborellales	Amborellaceae	Amborella_trichopoda	AF197813
	Austrobaileyales	Austrobaileyaceae	Austrobaileya_scandens	AF197742
	Austrobaileyales	Schisandraceae	Illicium_floridanum	AF197740
	Austrobaileyales	Schisandraceae	Kadsura_japonica	AF197738
	Austrobaileyales	Schisandraceae	Schisandra_sphenanthera	AF197739
	Austrobaileyales	Trimeniaceae	Trimenia_moorei	AF197741
	Nymphaeales	Cabombaceae	Brasenia_schreberi	AF197728
	Nymphaeales	Cabombaceae	Cabomba_sp._Qiu_97027	AF197729
	Nymphaeales	Nymphaeaceae	Nuphar_sp._Qiu_M114	AF197726
	Nymphaeales	Nymphaeaceae	Nymphaea_sp._Qiu_91029	AF197727
Gymnosperms				
	Coniferales	Pinaceae	Larix_decidua	DQ087882
	Coniferales	Pinaceae	Larix_gmelinii	DQ087887
	Coniferales	Pinaceae	Larix_griffithiana	DQ087889
	Coniferales	Pinaceae	Larix_kaempferi	DQ087883
	Coniferales	Pinaceae	Larix_laricina	DQ087881
	Coniferales	Pinaceae	Larix_lyallii	DQ087885
	Coniferales	Pinaceae	Larix_mastersiana	DQ087888
	Coniferales	Pinaceae	Larix_occidentalis	DQ087886
	Coniferales	Pinaceae	Larix_potaninii	DQ087890
	Coniferales	Pinaceae	Larix_sibirica	DQ087884

Coniferales	Pinaceae	Picea_chihuahuana	DQ414430
Coniferales	Pinaceae	Picea_martinezii	DQ414431
Coniferales	Pinaceae	Pinus_sp._Qiu_94013	AF197723
Coniferales	Podocarpaceae	Podocarpus_macrophyllus	DQ007425
Cycadales	Cycadaceae	Cycas_revoluta	AF197720
Cycadales	Zamiaceae	Zamia_integrifolia	AF197721
Ginkgoales	Ginkgoaceae	Ginkgo_biloba	AF197722
Gnetales	Gnetaceae	Gnetum_gnemon	AF197718
Welwitschiales	Welwitschiaceae	Welwitschia_mirabilis	AF197719

Table S5. Taxon information for 5-gene analysis

Species	atp1	cob	cox1	cox2	cox3
Silene_acaulis	EF547203	AF530621	EF547218	EF547229	EF547232
Silene_dioica	–	EF547209	EF547219	–	–
Silene_douglasii	–	EF547210	EF547220	–	–
Silene_latifolia	EF547204	AF530630	EF547221	–	EF547233
Silene_noctiflora	EF547205	EF547211	EF547222	EF547230	EF547234
Silene_nutans	–	EF547212	EF547223	–	–
Silene_rupestris	–	EF547213	EF547224	–	–
Silene_scouleri	–	EF547214	EF547225	–	–
Silene_virginica	–	EF547215	EF547226	–	–
Silene_vulgaris	–	EF547216	EF547227	–	–
Gypsophila_repens	–	EF547207	EF547217	–	–
Stellaria_sp.	EF547206	AF530631	EF547228	–	–
Beta_vulgaris	BA000009	BA000009	X57693	BA000009	BA000009
Nepenthes_sp.	EF547202	EF547208	AY600110	–	EF547231
Plantago_lanceolata	AY818937	unpubl. (JPM)	AJ389611	unpubl. (JPM)	unpubl. (JPM)
Plantago_rugelii	AY818939	AY818946	AJ389606	AY818947	–
Nicotiana_tabacum	BA000042	BA000042	BA000042	BA000042	BA000042
Daucus_carota	AF301604	AY007816	AY820131	X63625	–
Sambucus_sp.	AY741813	DQ317064	AF193965	–	DQ317066
	(S._sieboldiana)	(S._canadensis)	(S._canadensis)		(S._canadensis)
Erodium_pelargoniflorum	DQ317061	–	DQ317039	–	–
Pelargonium_x_hortorum	DQ317063	DQ317065	DQ317047	DQ317069	DQ317067
Oenothera_sp.	X04023	X07126	X05465	X00212	X04764
	(O._biennis)	(O._sp.)	(O._berteriana)	(O._villaricae)	(O._berteriana)
Arabidopsis_thaliana	Y08501	Y08501	X94583	Y08501	Y08501
Pisum_sativum	X05366	AJ132231	X14409	AJ414385	–
Platanus_occidentalis	AY009423	AY832084	AY009450	–	AY832117
Mahonia_bealei	AF197659	AY832081	–	–	AY832113
Laurus_nobilis	AF197682	AY832079	AF193956	AY832094	AY832111
Liriodendron_tulipifera	AY394730	AY832080	AF193959	AY832090	AY832112
Piper_sp.	AF197630	AY832083	AY009448	AY832091	AY832116
	(P._betle)	(P._betle)	(P._bicolor)	(P._betle)	(P._betle)
Eichhornia_sp.	AY299762	AY832078	–	AY832093	AY832110
	(E._azurea)	(E._crassipes)		(E._crassipes)	(E._crassipes)
Oryza_sativa	BA000029	BA000029	BA000029	BA000029	BA000029
Phoenix_sp.	U58831	DQ916691	AY166800	–	AY166801
	(P._reclinata)	(P._dactylifera)	(P._dactylifera)		(P._dactylifera)
Philodendron_hederaceum	–	AY832082	AJ223438	AY832095	AY832114
Acorus_sp.	AF039256	DQ916645	AF193944	AJ006146	–
	(A._calamus)	(A._gramineus)	(A._calamus)	(A._calamus)	
Nymphaea_sp.	AY299814	DQ916611	AF020570	–	AY832120
	(N._odorata)	(N._odorata)	(N._odorata)		(N._sp.)
Amborella_trichopoda	AY009407	AY832075	AY009430	AY832089	AY832106
Araucaria_heterophylla	AF209104	–	AF020558	–	–
Podocarpus_macrophyllus	AF209105	–	AF020575	–	–
Juniperus_virginiana	AF209106	–	AF020567	–	–
Pinus_sp.	AF209108	–	AF020574	–	AY832115
	(P._strobus)		(P._strobus)		(P._thunbergii)
Gnetum_sp.	AF197617	–	AF020566	–	X92722
	(G._gnemon)		(G._leyboldii)		(G._gnemon)
Ephedra_sp.	DQ646225	–	AF020564	–	X92735
	(E._distachya)		(E._viridis)		(E._gerardiana)
Ginkgo_biloba	AF209110	–	AF020565	AJ874265	X76280
Cycas_revoluta	AF197623	–	AF020562	DQ677484	X76279, X93553
Zamia_sp.	AF209111	–	AF020583	–	AY832118
	(Z._furfuracea)		(Z._furfuracea)		(Z._integrifolia)

Table S6. Taxon information for rDNA genes

Species	SSU rDNA	LSU rDNA
Silene_acaulis	EF547249	EF547246
Silene_latifolia	EF547250	EF547247
Silene_noctiflora	EF547251	EF547248
Beta_vulgaris	BA000009	BA000009
Plantago_lanceolata	AJ389619	unpublished (JPM)
Plantago_rugelii	AJ389615	AY818948
Nicotiana_tabacum	BA000042	BA000042
Sambucus_canadensis	AF194000	–
Erodium_pelargoniflorum	DQ317006	–
Pelargonium_x_hortorum	DQ317013	DQ317059
Oenothera_berteriana	X61277	X02559
Arabidopsis_thaliana	Y08501	Y08501
Platanus_occidentalis	AF161090	DQ008752
Mahonia_bealei	DQ008682	DQ008754
Laurus_nobilis	AF193990	DQ008773
Liriodendron_sp.	AF193993	DQ008786
	(L._tulipifera)	(L._chinense)
Piper_betle	AF161088	DQ008795
Oryza_sativa	BA000029	BA000029
Spathiphyllum_wallisii	DQ008673	DQ008814
Acorus_calamus	AF193976	DQ008817
Nymphaea_sp._Qiu_91029	AF161091	DQ008828
Amborella_trichopoda	AF193987	DQ008832
Araucaria_sp.	AB029364	DQ647874
	(A._excelsa)	(A._araucana)
Podocarpus_sp.	AB029369	DQ008837
	(P._costalis)	(P._macrophyllus)
Juniperus_chinensis	AB029368	–
Pinus_sp.	AF058659	DQ008835
	(P._strobus)	(P._sp._Qiu_94013)
Gnetum_gnemon	DQ008701	DQ008833
Ephedra_antisyphilitica	AF161084	–
Ginkgo_biloba	AB029355	DQ008838
Cycas_revoluta	AB029356	DQ008840
Zamia_integrifolia	AB029357	DQ008839

Table S7. Taxon information for Supplementary Figure S1

Species	matK
Silene_diclinis	EF547236
Silene_latifolia	EF547239
Silene_dioica	EF547237
Silene_vulgaris	EF547245
Silene_douglasii	EF547238
Silene_virginica	EF547244
Silene_scouleri	EF547243
Silene_noctiflora	EF547240
Silene_nutans	EF547241
Silene_rupestris	EF547242
Silene_acaulis	EF547235
Petrocoptis_pyrenaica	AY936314
Agrostemma_githago	AY042539
Cerastium_fontanum	AY936296
Stellaria_media	AY936299
Dianthus_seguieri	AY936321
Gypsophila_altissima	AY042597
Saponaria_officinalis	AY936325
Moehringia_trinervia	AY042615
Scleranthus_perennis	AY514847
Drypis_spinosa	AY936293
Illecebrum_verticillatum	AY514849
Polycarpon_tetraphyllum	AY936287
Herniaria_baetica	AY936283
Paronychia_kapela	AY936284
Corrigiola_litoralis	AY936331
Telephium_oligospermum	AY042664
Froelichia_floridana	AY514799
Amaranthus_greggii	AY514808
Bosea_yervamora	AY514810
Chenopodium_acuminatum	AY514836
Spinacia_oleracea	AJ400848
Beta_vulgaris	DQ116790
Achatocarpus_praecox	AY514845
Phaulothamnus_spinescens	AY514846
Mammillaria_haageana	AY015289
Portulaca_oleracea	AY875349
Anacampseros_vulcanensis	AY514851
Halophytum_ameghinoi	AY514852
Rivina_humilis	AY514850
